# Exploration of *Canarium odontophyllum* fruit phytoconstituents as potential candidates against epilepsy using in silico studies

**DOI:** 10.1016/j.jgeb.2025.100561

**Published:** 2025-08-26

**Authors:** Lim Joe Siang, Kamini Vijeepallam, Arunachalam Muthuraman, Parasuraman Pavadai, Thiruventhan Karunakaran, Veerasamy Ravichandran

**Affiliations:** a Research Scholar, Faculty of Pharmacy, AIMST University, Semeling-08100, Bedong, Kedah, Malaysia; b Faculty of Pharmacy, AIMST University, Semeling-08100, Bedong, Kedah, Malaysia; c Department of Pharmaceutical Chemistry, Faculty of Pharmacy, M S Ramaiah University of Applied Sciences, Bengaluru, Karnataka 560054, India; d Centre for Drug Research, Universiti Sains Malaysia, 11800 USM, Pulau Pinang, Malaysia; e Saveetha Dental College and Hospitals, Saveetha Institute of Medical and Technical Sciences, Chennai, Tamil Nadu, India

**Keywords:** Epilepsy, *Canarium odontophyllum*, Molecular docking, Pharmacokinetics, Drug-likeness profiles, Molecular dynamics

## Abstract

Epilepsy is the third most common neurological illness worldwide, affecting over 65 million people. Current antiepileptic drugs (AEDs) are ineffective for about 30 % of epileptic patients. AED use is extremely expensive and associated with adverse effects such as ataxia, nystagmus, and central nervous system depression. Serious quality-of-life changes are another aspect of managing epileptic conditions. A variety of therapeutic plant components, including flavonoids, phenolics, terpenoids, and alkaloids, exhibit a wide range of biological actions, including antibacterial, antioxidant, antiulcer, antineoplastic, anti-inflammatory, antiepileptic, and immunostimulant properties. Because many of them are being researched in modern research to develop an active therapeutic candidate, we resorted to medicinal plants to investigate powerful antiepileptic components. So, in this in silico study, we aimed to identify potential antiepileptic agents from *Canarium odontophyllum* fruits using molecular docking, molecular dynamics, and ADMET prediction approach. In the present study, the virtual screening was performed on a set of 63 phytocompounds, identified from the hydroalcoholic (70 %) extract of *C. odontophyllum* fruit using LC-MS (LC-MS-QTOF) analysis, by using molecular docking simulation to examine their binding ability with seven proteins that are responsible for epilepsy. Further, the selected compounds with good binding affinity were subjected to ADMET screening to predict the pharmacokinetic properties, and molecular dynamics testing was done to confirm the outcomes. Our results revealed that *C. odontophyllum* phytoconstituents showed the strongest binding affinities against all tested enzymes. Moreover, kanzonol B could prominently interact with all tested proteins with moderate to good binding affinity (−6.70 to −10.65 kcal/mol). All top phytoconstituents obeyed Lipinski’s RO5 and drug-likeness properties. These computational studies suggested that the phytoconstituents of *C. odontophyllum* can serve as potent inhibitors of enzymes responsible for epilepsy. Thus, we recommend further experimental investigations to validate the promising results of the present in silico study.

## Highlights

• Sixty-three phytoconstituents were identified from *Canarium odontophyllum* fruits.
• Molecular docking studies indicate kanzonol B has a significant binding affinity towards enzymes responsible for epilepsy.
• Molecular dynamics studies confirm the stability of the protein-kanzonol B complex.
• Kanzonol B passes the Lipinski rule of five for oral bioavailability.
• In-silico toxicity prediction confirms that kanzonol B is free from toxicity.

## Graphical abstract

**Figure:**
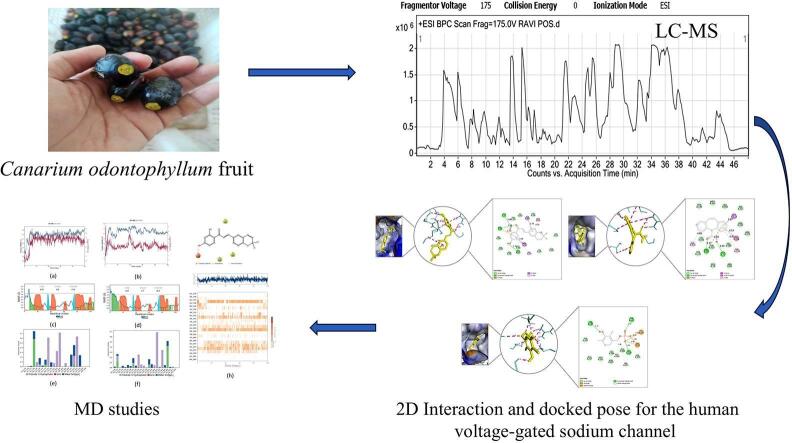

## 1 Introduction

The pathophysiologic basis of epilepsy is an altered equilibrium of the excitatory and inhibitory neurotransmission present in the central nervous system leading to coordinated firing and neuronal hyper-excitability. The malfunctioning of voltage-gated ion channels, particularly, calcium and sodium, excessive glutamate signaling and/or insufficient GABA repression often occur as the dysregulations. The goal of antiepileptic drugs (AEDs) is to restore this balance. They act in numerous ways, including a direct modulation of ion channels and stabilized of neuron membranes and inhibition of paroxysmal depolarization, a decreased glutamatergic excitation and an enhanced GABA-induced inhibition. The current pharmacological treatment consists of many medications that have varying efficacy in different types of seizures and epilepsy syndromes. These drugs can be divided into those which belong to the first line (phenytoin, carbamazepine, valproate) and more recent (lamotrigine, levetiracetam, lacosamide, perampanel). There are a lot of key factors contributing to a large and ongoing study in this setting. Most noteworthy, a good population of patients, about one-third or so, enjoys drug-resistant epilepsy, where the patient still suffers seizures despite various AED therapies. In addition, some of the existing drugs are associated with severe side effects that reduce the quality of life of a patient, including mood alterations, effects on cognition, as well as systemic toxicities. Moreover, epilepsy is the third most common neurological condition in the world, with over 65 million people having the disease, and in Malaysia alone, about 1 in every 100 individuals has epilepsy. The utilization of AEDs is also expensive. It is, therefore, important to design newer AEDs with multitargeting and effective agents with an acceptable profile to increase the control of seizures and hopefully cure them.^1^

Canarium species of the Burseraceae family have been associated with neuroprotective benefits on a range of neurological disorders, including Alzheimer's disease. In addition, *Canarium odontophyllum* Miq (*C. odontophyllum*) contains significant amounts of phenolic, flavonoid, tannin, and anthocyanin compounds, all of which are associated with its antioxidant and anti-epileptic activities.^2^, ^3^, ^4^ However, studies exploring the benefits of *C. odontophyllum* in these aspects are still lacking*.*

The time, expense, and effort needed to find new drugs can be decreased by using computer-assisted drug design to predict the pharmacokinetic, stability, and efficacy of candidate drugs before synthesis, as opposed to the conventional method (synthesis, in vitro, and in vivo studies). In the present study, we have used molecular docking studies to explore the antiepileptic potency of 63 phytoconstituents present in *C. odontophyllum* against epileptic target proteins. In order to assess the effectiveness and safety of the phytoconstituents of *C. odontophyllum*, we have also investigated the ADMET characteristics.

## 2 Materials and methods

### 2.1 Preparation of C. odontophyllum fruit extracts

**Fig. 1:**
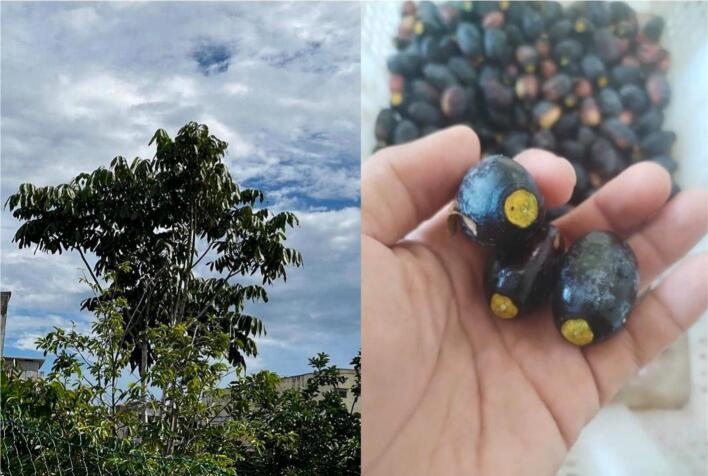
Plant and fruits of *Canarium odontophyllum* (Dabai).

*C. odontophyllum* (dabai) fruits were procured from a cultivator in Sarawak, Malaysia. The fruits were then identified and authenticated by a botanist at Universiti Sains Malaysia, USM, with herbarium number (USMP 12392). The fruit flesh, intact with skin, was separated from the seeds and cut into smaller pieces. After that, these pieces were left to dry for seven days at room temperature in the shade. After drying, the pieces were ground into coarse powder. The coarse fruit powder was then extracted with hydro-alcohol (ethanol:water 70 %:30 %) using the Soxhlet extraction method for 48 to 72 h at 70 °C. Because of its efficacy in extracting phenolic chemicals, a hydro-alcohol mixture was utilized for extraction rather than alcohol alone.^5^ After extraction, the extracts were concentrated at 50 °C using a rotary evaporator. The condensed extract of *C. odontophyllum* fruits (COFE) was then stored in a desiccator for further use. The plant and fruit of the *C. odontophyllum* are shown in Fig. 1.

### 2.2 Liquid chromatography-Mass spectroscopy (LC-MS) analysis

The LC-MS analysis of COFE was carried out at the service laboratory of the University Malaya, Malaysia, using the Agilent 6550 iFunnel LC-MS-QTOF system equipped with HiP sampler and a binary pump. The LC-MS instrument includes an LC system fitted with a Dual AJS electrospray ionization (ESI) source and a Q-TOF Mass Spectrometer. The extract separation was performed on a C-18 column Zorbax Eclipse Plus (100 × 4.5 mm, 3.5 μm), using a gradient elution approach with a mobile phase made up of water and acetonitrile in various gradients, which were eluted at 1 ml/min. The mass spectra (MS) were obtained from both positive and negative electrospray modes, and the mass scan rate was 1.00 spectra/sec, with a mass scan range of 100 to 1700 *m*/*z*.

### 2.3 Molecular docking studies

**Fig. 2:**
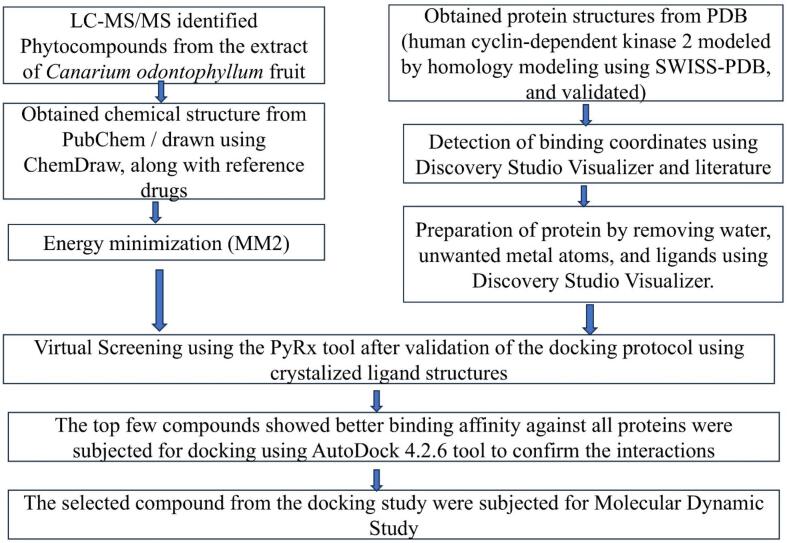
Scheme for in silico studies of phytocompounds identified from *Canarium odontophyllum* fruits.

Online databases including Protein Data Bank (https://www.rcsb.org/) and PubChem (https://pubchem.ncbi.nlm.nih.gov/) were used to retrieve the structures of proteins and chemical compounds, respectively. Additionally, the modeling software such as Chem Office-16 (https://chemistrydocs.com/chemoffice-2016-chemdraw-professional-2016/), Discovery Studio Visualizer 2020 (https://discover.3ds.com/discovery-studio-visualizer-download), SWISS-MODEL (https://swissmodel.expasy.org/), ModLoop (https://modbase.compbio.ucsf.edu/ modloop/), AutoDock 4.2.6 and AutoDock Tool 1.5.7 (https://ccsb.scripps.edu/mgltools/ downloads/), PyRx (https://pyrx.sourceforge.io/downloads/), Swiss-PdbViewer (https://spdbv. unil.ch/) and PyMOL Win (https://www.pymol.org/), online tool SwissADME (https://www. swissadme.ch/), SAVES 6.0 (https://saves.mbi.ucla.edu/), ProSA (https://prosa.services.came. sbg.ac.at/prosa.php), ProQ (https://proq.bioinfo.se/ProQ/ProQ.html), ProTox 3.0 (https://tox.charite.de/protox3/) and pkCSM (https://biosig. lab.uq.edu.au/pkcsm/ prediction) and UCSF-Chimera (https://www.cgl.ucsf.edu/chimera/) were used in the present study. The flow chart of in-silico studies is shown in Fig. 2.

### 2.4 Preparation of ligands

The structures of COFE phytocompounds were downloaded from PubChem as.sdf files, and the unavailable compounds were drawn using ChemDraw software (Table S1). Structures of a few standard FDA-approved antiepileptic drugs were also obtained from PubChem. The ligand structures were then converted to 3D structures and subjected to energy minimization using MM2 force field techniques in the Chem3D tool of Chem Office-16. Before each structure was saved as an .sdf file for later usage, hydrogens and charges were added.

### 2.5 Preparation of proteins

The proteins that are associated with epilepsy, such as gamma-aminobutyric acid receptor, gamma-aminobutyrate aminotransferase, human mitochondrial branched-chain aminotransferase, human voltage-gated sodium channel, α-amino-3-hydroxy-5-methyl-4-isoxazolepropionic acid receptor, human cyclin-dependent kinase 2, and human arginase I, were used in the present study, and the three-dimensional structures of these proteins were acquired from the Protein Data Bank. Only non-mutated proteins with resolutions of less than 3 Å were selected for the current study.^6^ Using PyMOL, the proteins' crystallographic structures were examined for flaws and broken chains, and they were fixed by using the SWISSPDB viewer. Missing residues at the end of protein chains were repaired using PyMOL, while those missing in the middle were modelled (homology) using SWISS-MODEL. Then the modeled protein was energy minimized in the SWISS-PDB viewer tool and in UCSF-Chimera and then validated.

### 2.6 Modeled protein quality assessment

SAVES v6.0 was used to evaluate the prepared protein structures. The protein structures in PDB format were uploaded in SAVE v 6.0, and the relevant parameters were evaluated. Three programs, namely ERRAT, Verify3D, and PROCHECK, were used for model quality assessment. The modeled structures with the better evaluation outcome were selected for further analysis.^7^

### 2.7 Active sites determination

The active sites (XYZ coordinates) for the protein complex with ligand were determined using the Discovery Studio Visualizer 2020. A blind docking method was employed for the gamma-aminobutyric acid GABA(A) receptor (PDB ID: 1gnu), which is an apoprotein with an unknown active site. For proteins Human Voltage-gated Sodium Channel, brain isoform (Nav1.2) (PDB ID: 2kav) and Leucine-rich glioma inactivated 1 (LGI1) (PDB ID: 5y30), active site information was obtained from earlier reported studies. GLY 1870, SER 1869, GLU 1868, LEU 1866, VAL 1865, LEU 1790, PRO 1789, GLU 1788, GLU 1785, SER 1869, and GLU 1785 residues were revealed in the ligand binding pocket of 2kav,^8^ while GLU81, ILE61, ILE82, PHE79, SER60, SER83, and SER86 were reported in the binding pocket of 5y30.^9^

### 2.8 Docking protocol validation

The redocking approach was used to validate the docking protocol.^10^ The crystallographic structure of the ligand was extracted from the ligand–protein complex and re-docked against its respective protein active site using PyRx (AutoDock). Then, the RMSD value between the ligand's re-docked pose and the ligand's original crystallographic structure was determined using PyMOL/Discovery Studio Visualizer.

### 2.9 Virtual screening

The PyRx GUI's Autodock Wizard was used for virtual screening. The ligands and proteins were converted into.pdbqt format in the PyRx tool. The grid box was created according to the identified active sites. The prepared ligand structures were docked against the proteins. During the docking process, the protein was kept rigid, while the ligands were treated as flexible entities. Autodock Wizard produced up to 9 conformations for each compound. For each phytocompound, the conformations with the highest negative binding energy were chosen for additional examination.

### 2.10 Molecular docking studies

AutoDock tool 1.5.7 was used for docking investigations (AutoDock 4.2.6). In order to prepare the proteins, polar hydrogens, Kollman charges, and water were removed. Gasteiger charges were introduced after the ligands were protonated. After that, the ligands and proteins were both saved in the “.pdbqt” format. By entering the X, Y, and Z coordinate values that were determined using Discovery Studio Visualizer, the active grid box was modified.^11^ Except for the maximum number of evaluations (long), population size (300), and number of genetic algorithm (GA) trials (50), all docking parameters were set to their default levels. The protein–ligand complex underwent docking investigations using the Lamarckian Genetic Algorithm.^12^ The optimal conformer was chosen based on the binding affinity once the docking studies were finished. Discovery Studio Visualizer 2020 was then used to examine the docking output files for interactions between substances and the protein's amino acid residues. For further discussion on binding interactions between the ligand and the target, the docking pose and 2D interactions were illustrated and preserved.^13^

### 2.11 Drug likeness and ADMET prediction

The SMILES code of the phytocompounds was obtained by using the Chem3D software. The ADME characteristics of all the phytocompounds from COFE were determined using SwissADME, and the pkCSM software was used to compute the BBB and CNS permeabilities.^14^, ^15^ SwissADME provided information on the molecular weight, logP, number of hydrogen donors, hydrogen acceptors, rotatable bonds, and total polar surface area. The drug-likeness of the compounds was predicted based on Lipinski's rule and Veber’s rule. Toxicity predictions, including neurotoxicity, hepatotoxicity, cardiotoxicity, nephrotoxicity, carcinogenicity, immunotoxicity, mutagenicity, cytotoxicity and clinical toxicity were carried out using SwissADME.

### 2.12 Molecular dynamics simulation studies

The DESMOND computer program was developed by the D.E. Shaw research group to compute the molecular dynamics (MD) simulation of protein–ligand complexes (PLC).^16^ MD simulation modeling highlights the potential effects of PLC at target binding sites in physiological settings (Academic license, Version 2020–1, Schrödinger, LLC, New York, NY, USA, 2021–4, Schrödinger Release: QikProp). A 10 × 10 × 10 box was constructed to hold physiological properties, including pH and water molecules.^17^ Na^+^ or Cl^-^ ions may be added if the pH is absent or required to be changed to meet the specific requirements of the research methodology. The TIP3P water solvation model was used to solve the docked protein–ligand complexes. The physiological salt content was maintained at 0.15 M while counter ions were employed to neutralize the solvated system. The OPLS AA (Optimal Potentials for Liquid Simulation-All Atom) force field was applied to the PLC system.^18^ Using the system builder panel, moderate minimization was carried out on the prepared PLC at roughly 100 ps. As a result, the prepared system stabilized in response to its surroundings. The Nose-Hoover chain thermostat, the Martyna–Tobias–Klein barostat, and the Reversible Reference System Propagator Algorithms (RESPA) integrator were used to conduct molecular dynamics with relaxation lengths of 2 ps.^19^, ^20^ The final MD simulation was generated using the equilibrated system. The MD simulation was run for 100 ns at 310.15 K temperature and 1.0 bar pressure with the default relaxation parameters and the NPT (Isothermal-Isobaric ensemble, constant temperature, constant pressure, and constant number of particles) ensemble.^21^ After the simulation was complete, the findings were evaluated using a simulation interaction diagram.^22^

## 3 Results

Soxhlet extraction is an exhaustive method of extraction, ensuring complete extraction of analytes from the plant matrix.^23^, ^24^, ^25^ In the present study, the extraction value of *C. odontophyllum* fruits was 7.87 % w/w.

### 3.1 LC-MS analysis

Comprehensive analysis on COFE using the MS/MS spectra revealed a total of 63 compounds, of which 49 compounds were detected in positive ionization mode (Supplementary Fig. S1) and 14 compounds were detected in negative ionization mode (Supplementary Fig. S1) (Table 1 and Supplementary Tables S1 and S2). The extract was found to comprise alkaloids, lipids, carbohydrates, heterocyclic compounds, aromatic compounds, and peptides (Supplementary Tables S3).

**Table 1:** Phytoconstituents identified from the hydro-alcoholic extract of *C. odontophyllum* fruits by LC-MS analysis.

Code	Proposed Compounds	RT (min)	MW	Formula
PDFC1	Nigerose (Sakebiose)	3.85	342.1174	C_12_H_22_O_11_
PDFC2	2-(beta-D-Glucosyl)-*sn*-glycerol	4.08	254.1006	C_9_ H_18_O_8_
PDFC3	Neuraminic acid	6.08	267.0937	C_9_H_17_NO_8_
PDFC4	9Z,11E,13-Tetradecatrienal	6.72	206.1667	C_14_H_22_O
PDFC5	3′-Hydroxytrimethoprim	6.79	276.1221	C_13_H_16_N_4_O_3_
PDFC6	Leu Pro	7.04	228.1485	C_11_H_20_N_2_O_3_
PDFC7	Tranylcypromine glucuronide	7.74	309.1221	C_15_H_19_NO_6_
PDFC8	*cis*-Zeatin	8.31	219.1120	C_10_H_13_N_5_O
PDFC9	Asn Tyr Thr	9.27	396.1644	C_17_H_24_N_4_O_7_
PDFC10	Abscisate	9.75	264.1372	C_15_H_20_O_4_
PDFC11	4-Methylesculetin	11.04	192.0433	C_10_H_8_O_4_
PDFC12	Val-Val-OH	11.98	324.1336	C_15_H_20_N_2_O_6_
PDFC13	1-Methyl-4-nitro-5-(S-Gluctathionyl) imidazole	14.05	432.1086	C_14_H_20_N_6_O_8_S
PDFC14	3,5,7,2′,5′-Pentahydroxyflavone	14.27	302.0437	C_15_H_10_O_7_
PDFC15	5,7,2′-Trihydroxy 7-glucoside	14.36	432.1069	C_21_H_20_O_10_
PDFC16	Neovitexin	14.60	432.1067	C_21_H_20_O_10_
PDFC17	(b-D-Glucopyranuronic acid, 1-[2-[(2,3-dimethylphenyl)amino] benzoate])	15.99	417.1436	C_21_H_23_NO_8_
PDFC18	Colnelenic acid	16.79	292.2049	C_18_H_28_O_3_
PDFC19	9-Hydroperoxy-12,13-epoxy-10-octadecenoic acid	17.07	328.2261	C_18_H_32_O_5_
PDFC20	Betaxolol	18.33	307.2136	C_18_H_29_NO_3_
PDFC21	8,11-Octadecadiynoic acid	21.20	276.2101	C_18_H_28_O_2_
PDFC22	Levuglandin E2	21.44	352.2242	C_20_H_32_O_5_
PDFC23	9S,10S,11R-Trihydroxy-12Z-octadecenoic acid	22.07	330.2416	C_18_H_34_O_5_
PDFC24	12,13S-Epoxy-9Z,11-octadecadienoic acid	22.18	294.2207	C_18_H_30_O_3_
PDFC25	Hinokiflavone	22.33	538.0913	C_30_H_18_O_10_
PDFC26	4-Oxo-9Z,11Z,13E,15E-octadecatetraenoic acid	22.63	290.1895	C_18_H_26_O_3_
PDFC27	19-Norandrostenedione	22.79	272.1789	C_18_H_24_O_2_
PDFC28	Trans-EKODE-(E)-Ib	23.79	310.2155	C_18_H_30_O_4_
PDFC29	5-Oxo-ETE-d7	24.40	325.2634	C_20_H_23_D_7_O_3_
PDFC30	Asn Asn Arg	25.21	402.1963	C_14_H_26_N_8_O_6_
PDFC31	Bavachromanol	25.90	340.1323	C_20_H_20_O_5_
PDFC32	Kanzonol B	26.39	322.1214	C_20_H_18_O_4_
PDFC33	9E,12Z,15Z-Octadecatrienoic acid	26.61	278.2255	C_18_H_30_O_2_
PDFC34	Dihomo-PGI2	27.30	380.2551	C_22_H_36_O_5_
PDFC35	9,13-Dihydroxy-10-ethoxy-11-octadecenoic acid	27.58	358.2731	C_20_H_38_O_5_
PDFC36	7-Methoxychromone	28.05	176.0482	C_10_H_8_O_3_
PDFC37	Clobetasol propionate	29.01	466.1909	C_25_H_32_ClFO_5_
PDFC38	2-Butyl-3-[[4-[2-(2*H*-tetrazol-5-yl)phenyl]phenyl]methyl]-1,3-diazaspiro[4.4]non-1-en-4-one	29.35	428.2336	C_25_H_28_N_6_O
PDFC39	Arg Thr Phe	29.49	422.2263	C_19_H_30_N_6_O_5_
PDFC40	5-Hydroxyfluvastatin	30.15	427.18	C_24_H_26_FNO_5_
PDFC41	15-Epi-15-A2t-IsoP	30.67	334.2135	C_20_H_30_O_4_
PDFC42	L-Glutamic acid dibutyl ester	32.11	259.1773	C_13_H_25_NO_4_
PDFC43	Mayolene-18	36.85	560.4820	C_36_H_64_O_4_
PDFC44	12-Hydroxy-10-octadecynoic acid	37.61	296.2365	C_18_H_32_O_3_
PDFC45	9E,12Z,15Z-Octadecatrienoic acid	37.84	278.2258	C_18_H_30_O_2_
PDFC46	N-Hexadecanoyl-L-Homoserine lactone	40.35	339.2775	C_20_H_37_NO_3_
PDFC47	17,20-Dimethyl Prostaglandin F1α	41.42	384.2860	C_22_H_40_O_5_
PDFC48	7,11,14-Eicosatrienoic acid	42.55	306.2565	C_20_H_34_O_2_
NDFC1	Mitoxantrone	9.88	444.1999	C_22_ H_28_N_4_O_6_
NDFC2	Gln Ala Tyr	12.91	380.1693	C_17_H_24_N_4_O_6_
NDFC3	Robustaflavone	23.20	538.0892	C_30_H_18_O_10_
NDFC4	12-Octadecenoic acid, 9,10,18-trihydroxy-; 9,10,18 Trihydroxyoctadec-12-enoic acid	23.35	330.2413	C_18_H_34_O_5_
NDFC5	9-Hydroperoxy-12,13-epoxy 10-octadecenoic acid	24.66	328.2246	C_18_H_32_O_5_
NDFC6	9,13-Dihydroxy-10-ethoxy-11 octadecenoic acid	28.51	358.2712	C_20_H_38_O_5_
NDFC7	2-(Cyclohexanecarbonyl)-3,6,7,11*b*-tetrahydro-1*H*-pyrazino[2,1-a]isoquinolin-4-one	29.36	312.1834	C_19_H_24_N_2_O_2_
NDFC8	Lamioside	30.44	420.1579	C_18_H_28_O_11_
NDFC9	Ajmaline	30.97	326.1998	C_20_H_26_N_2_O_2_
NDFC10	4,14-Dihydroxy-octadecanoic acid	33.64	316.2613	C_18_H_36_O_4_
NDFC11	13(R)-HODE	35.82	296.2348	C_18_H_32_O_3_
NDFC12	9S,10R-Epoxy-stearic acid	37.88	298.2502	C_18_H_34_O_3_
NDFC13	*N*-[4-[2-Hydroxy-3-(propan-2-ylamino)propoxy]phenyl]acetamide	40.26	266.1635	C_14_H_22_N_2_O_3_
NDFC14	2-Chloropalmitaldehyde	43.92	274.2042	C_16_H_31_ClO

### 3.2 Molecular docking studies

#### 3.2.1 Modeled protein

**Fig. 3:**
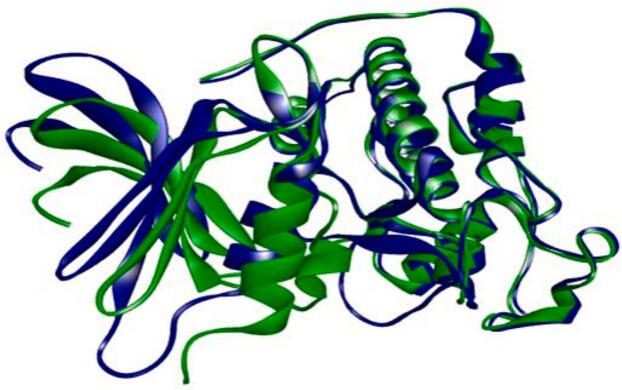
Alignment of the original Human Cyclin Dependent Kinase 2 protein (1pxo) and modeled protein structure.

The human cyclin-dependent kinase 2 was modeled (homology) using PDB ID: 1pxo as a template in the SWISS-MODEL online tool. The alignment of the original protein structure against the modelled protein structure was then carried out using PyMOL molecular viewer. Fig. 3 displays the alignment of the modelled protein (blue color) and the original protein (green color). The RMSD for these structures was found to be 1.305 Å (1776 to 1776 atoms). Similarly, the PyMOL modelled proteins were also aligned against their original proteins, yielding RMSD value of less than 2 Å. The other proteins were used as such, downloaded from PDB.

#### 3.2.2 Modeled protein validation

Protein structure verification servers such as SAVES 6.0 and ProSA-web were used to assess the quality of the models. The ERRAT, overall G-factor, and percentage of residues in the allowed region were calculated by the SAVES 6.0 server. Supplementary Fig. S3 demonstrates that more than 90 % of amino acid residues were mainly found in the most favored and additionally allowed regions. In addition, the overall G-factor was found to be > −0.5. In the current study, modeled human cyclin-dependent kinase 2 showed ERRAT values above 90 %. The ProSA Z-scores were calculated using the ProSA website, and the modelled protein fell within the Z-score range of −1 to −13 (Supplementary Fig. S3). The local model quality estimation for the modeled protein indicates a lower energy level than zero. The quality of the protein structure was also estimated using ProQ, which revealed that the predicted LG score for the modeled protein was >6, indicating an extremely good model (LG score > 4). Additionally, the verify3D values were also calculated for the modeled protein, and it showed high scores (>80 %).

#### 3.2.3 Docking protocol validation

The accuracy of the docking protocol was validated by re-docking, and the extracted ligand from the crystallographic protein–ligand complex structures was redocked with the same protein. The best-ranked poses were superimposed onto their co-crystallized poses, and the RMSD was determined (Table 2).

**Table 2:** RMSD of docked crystal ligand structure against the original crystal ligand structure.

Protein	PBD ID	Crystal ligand name	RMSD (Å)
Gamma-aminobutyric acid A receptor (GABA(A))	1gnu	−-	−-
Gamma-aminobutyrate aminotransferase (GABA-T)	1sff	(4′-Deoxy-4′-Acetylamino-Pyridoxal-5′-Phosphate)	1.640
Human mitochondrial branched chain aminotransferase (BCATm)	2a1h	(Pyridoxal-5′-Phosphate)	1.740
Human voltage-gated sodium channel, brain isoform (Nav1.2) (HVGSC)	2kav	−-	−-
α-amino-3-hydroxy-5-methyl-4-isoxazolepropionic acid receptor (AMPA)	3dp4	((S)-Alpha-Amino-3-Hydroxy-5-Methyl-4-Isoxazolepropionic acid)	1.052
Human Cyclin Dependent Kinase 2 (CDK2)	Modeled (1pxo)	([4-(2-Amino-4-Methyl-Thiazol-5-YL)-Pyrimidin-2-YL]-(3-Nitro-Phenyl)-Amine)	2.790
Leucine-rich glioma inactivated 1 (LGI1)	5y30	−-	−-
Human arginase I (ARG1)	3thj	(L-ornithine)	1.950

As shown in Table 2, all the binding conformations of the re-docked ligand within the binding pocket of the protein produced by the PyRx were like the binding mode of the co-crystallized ligand, and the RMSD values were below 2 Å, except 1pxo, which had 2.79 Å.

#### 3.2.4 Virtual screening

Sixty-three phytocompounds that were identified from the LC-MS analysis along with the selected reference FDA-approved drugs (Supplementary Table S4), were subjected to virtual screening. After applying filters of the binding affinity, the top three compounds that exhibited the highest binding affinity for each receptor were selected. Supplementary Table S5 summarizes the virtual screening results. Based on virtual screening, the best compound based on the binding score, binding interactions, and diversity was found to be kanzonol B (PDFC32).

#### 3.2.5 Docking studies

**Fig. 4:**
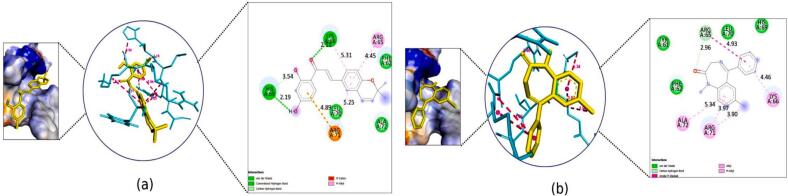
Docked Pose, 3D and 2D Interaction for the GABA(A) with **(a)** Kanzonol B, **(b)** Diazepam.

**Fig. 5:**
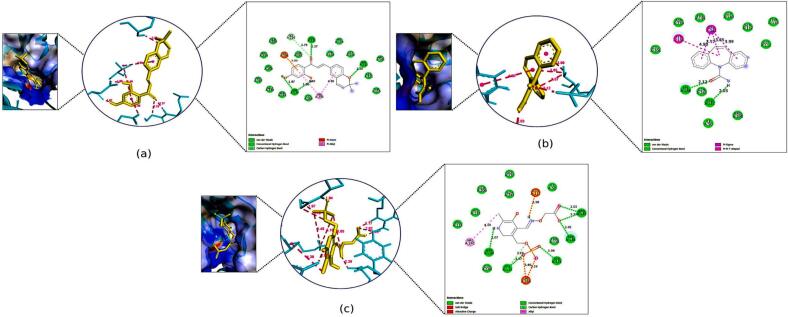
2D Interaction and docked pose for the GABA-T complex with (a) Kanzonol B, (b) Carbamazepine, (c) Crystallographic ligand.

**Fig. 6:**
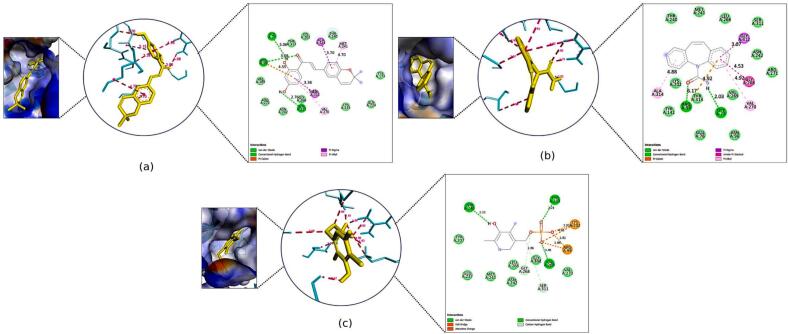
2D Interaction and docked pose for the BCATm complex with (a) Kanzonol B, (b) Carbamazepine, (c) Crystallographic ligand.

**Fig. 7:**
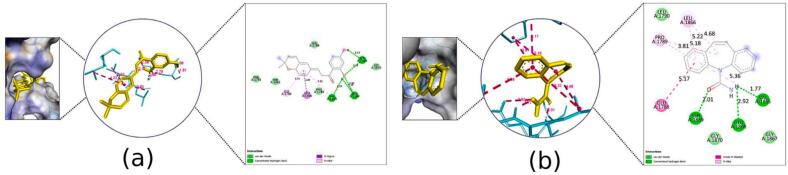
2D Interaction and docked pose for the human voltage-gated sodium channel, brain isoform complex with (a) Kanzonol B, (b) Carbamazepine.

**Fig. 8:**
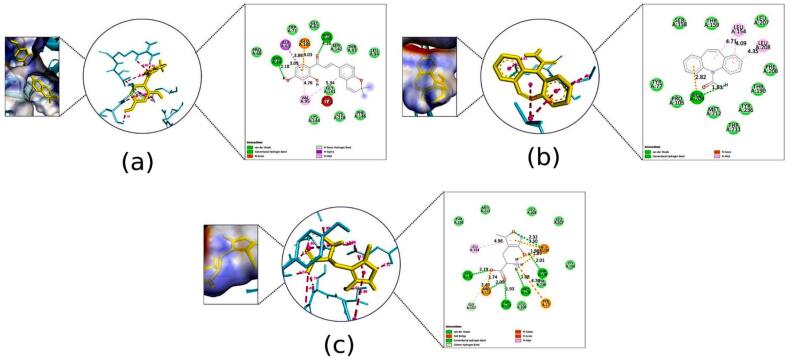
2D Interaction and docked pose for the α-amino-3-hydroxy-5-methyl-4-isoxazolepropionic acid complex with (a) Kanzonol B, (b) Carbamazepine, (c) Crystallographic ligand.

**Fig. 9:**
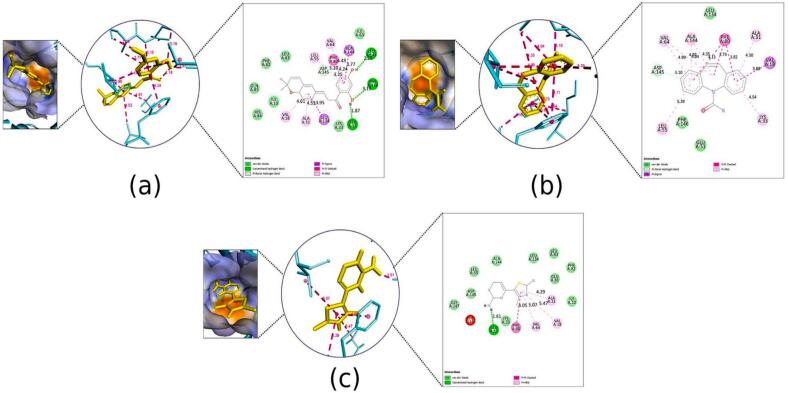
2D Interaction and docked pose for the human cyclin dependent kinase 2 complex with (a) Kanzonol B, (b) Carbamazepine, (c) Crystallographic ligand.

**Fig. 10:**
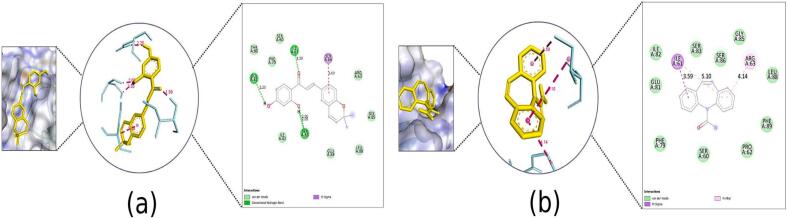
2D Interaction and docked pose for the leucine-rich glioma inactivated 1 complex with (a) Kanzonol B, (b) Carbamazepine.

**Fig. 11:**
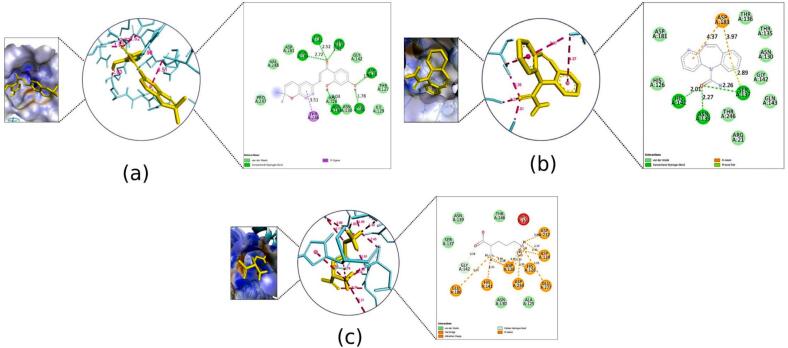
2D Interaction and docked pose for the human arginase I complex with (a) Kanzonol B, (b) Carbamazepine, (c) Crystallographic ligand.

The binding affinities of kanzonol B and the standard drugs to the selected proteins are shown in Table 3. The binding interaction surface poses and 2D interactions on receptors are shown in Fig. 4, Fig. 5, Fig. 6, Fig. 7, Fig. 8, Fig. 9, Fig. 10, Fig. 11. While the amino acid residues involved in interactions were revealed in supplementary Table S6.

**Table 3:** Docking scores of selected compounds from COFE and the standard drugs.

Protein	Compounds	Binding affinity (kcal/mol)	Standard drugs	Binding affinity (kcal/mol)
Gamma-Aminobutyric acid GABA(A) receptor	Kanzonol B (PDFC 32)	−6.70	Diazepam	−6.63
Gamma-aminobutyrate aminotransferase (GABA-T)	Kanzonol B (PDFC 32)	−10.65	Carbamazepine	−7.60
Human mitochondrial branched chain aminotransferase (BCATm)	Kanzonol B (PDFC 32)	−7.90	Carbamazepine	−7.11
Human Voltage-gated Sodium Channel, brain isoform (Nav1.2)	Kanzonol B (PDFC 32)	−7.92	Carbamazepine	−6.63
α-amino-3-hydroxy-5-methyl-4-isoxazolepropionic acid (AMPA) receptor	Kanzonol B (PDFC 32)	−8.30	Carbamazepine	−6.79
Human Cyclin Dependent Kinase 2	Kanzonol B (PDFC 32)	−10.04	Carbamazepine	−8.07
Leucine-rich glioma inactivated 1 (LGI1)	Kanzonol B (PDFC 32)	−8.07	Carbamazepine	−6.06
Human arginase I (ARG1)	Kanzonol B (PDFC 32)	−6.78	Carbamazepine	−5.74

##### 3.2.5.1 Binding to gamma-aminobutyric acid A (GABA(A)) receptor (PDB ID: 1gnu)

Kanzonol B showed molecular interactions at the active site residue of GABA(A) receptor with a binding score of −6.70 kcal/mol. As shown in Fig. 4a, kanzonol B formed three H-bond interactions with residue LYS 66 (2.11Å) and HIS69 (2.19Å and 3.54 Å) of the GABA(A) receptor, whereas hydrophobic interactions were formed with residues ARG65, LYS66 and ARG71. The standard drug (diazepam) showed one C–H bond at ARG65 (2.96 Å), five hydrophobic interactions with ARG65, ARG71 (2 bonds), ARG72 and LYS66 on the GABA(A) receptor with a binding score of −6.63 kcal/mol. Moreover, van der Waals interactions were also observed in the GABA(A) receptor with kanzonol B at PHE62, LEU70, and ALA72, whereas for diazepam at TYR61, PHE62, HIS69, and LEU70 (Fig. 4b).

##### 3.2.5.2 Binding to gamma-aminobutyrate aminotransferase (GABA-T) (PDB ID:1sff)

Kanzonol B showed strong interaction with the enzyme GABA-T, with a high binding score of −10.65 kcal/mol. As illustrated in Fig. 5a, kanzonol B formed five H-bonds with SER112 (2.20 Å), GLU211 (2.37 Å), ASP239 (1.87 Å and 1.86 Å), and GLY210 (3.79Å). Additionally, it formed two hydrophobic interactions with VAL241 and one electrostatic interaction with GLU206. In comparison, the standard drug (carbamazepine) formed two H-bonds with LYS268 (2.12 Å) and GLU211 (2.03 Å), along with four hydrophobic interactions with residues VAL241 (3 bonds) and TYR138 (Fig. 5b). The binding affinity of carbamazepine to the gamma-aminobutyrate aminotransferase (GABA-T) protein was weaker (−7.60 kcal/mol) as compared to kanzonol B (Fig. 5b).

Meanwhile, the crystallographic ligand extracted from the GABA-T protein model formed eight H-bond with GLU211 (2.38 Å), GLY111 (two bonds at 1.97 Å and 3.19 Å), SER112 (1.94 Å), ARG141 (two bonds at 2.17 Å and 2.03 Å), ASP239 (2.07Å) and TYR138 (2.92 Å) (Fig. 5c). Besides, it also exhibited one hydrophobic interaction with VAL241 and two electrostatic interactions with LYS268.

##### 3.2.5.3 Binding to human mitochondrial branched chain aminotransferase (BCATm) (PDB ID:2a1h)

In this study, as shown in Fig. 6a, kanzonol B fitted well into the active site of BCATm enzyme with the binding affinity of −7.90 kcal/mol. It formed three hydrogen bonds with the residues ARG99 (3.08 Å), SER311 (2.76 Å), and GLY77 (3.06 Å).

Four hydrophobic bonds with GLY312, ALA314, MET241, VAL270, and one electrostatic interaction at ARG99 were also noticed for the docking of kanzonol B on BCATm. In comparison, carbamazepine had showed a binding affinity of −7.11 kcal/mol, formed three H-bonds at ARG99 (2.83 Å and 1.75 Å), and GLY77 (2.03 Å), one electrostatic interaction at ARG99 and four hydrophobic interactions at GLY312, GLY268, ALA314 and VAL270, respectively (Fig. 6b). The crystallographic ligand formed seven H-bonds at ARG99 (1.88 Å), LYS202 (1.93 Å), VAL269 (1.81 Å), THR313 (2.21 Å), THR240 (2.12 Å), GLY268 (2.86 Å) and SER311 (3.50) (Fig. 6c). Among these, the hydrogen bonds formed at ARG99 and LYS202 were salt bridges, while GLY268 and SER311 were C–H bonds. Moreover, the ligand formed two electrostatic interactions with ARG99 and LYS202.

##### 3.2.5.4 Binding to human voltage-gated sodium channel, brain isoform (Nav1.2) (PDB ID: 2kav)

Kanzonol B exhibited binding affinity of −7.92 kcal/mol against the human voltage-gated sodium channel. Furthermore, six H-bonds at GLU1868 (2.90 Å and 2.38 Å), SER1869 (2.70 Å, 1.81 Å, and 2.53 Å), and VAL1865 (2.13 Å), along with three hydrophobic interactions at LEU1866, LEU1790, VAL1865 were observed (Fig. 7a).

Whereas, carbamazepine formed three H-bonds with SER1869 (2.01 Å), VAL1865 (1.77 Å), and GLU1868 (2.92 Å), as well as six hydrophobic interactions at GLU1788, PRO1789 (2 bonds), VAL1865, and LEU1866 (2 bonds) (Fig. 7b).

##### 3.2.5.5 Binding to α-amino-3-hydroxy-5-methyl-4-isoxazolepropionic acid (AMPA) (PDB ID: 3dp4)

Kanzonol B and carbamazepine showed molecular interactions on the active site residue of AMPA receptor with a binding score of – 8.30 and − 6.79 kcal/mol, respectively. In the kanzonol B complex, four H-bonds were observed at ARG96 (2.80 Å and 2.61 Å), ARG64 (2.18 Å and 3.09 Å), along with three hydrophobic interactions with the residue ALA63, VAL95 (2 bonds) (Fig. 8a). While, one H-bond at GLU209 (1.81 Å), three hydrophobic at LEU154 (2 bonds) and LEU208 and an electrostatic interaction were observed at GLU209 in carbamazepine complex (Fig. 8b).

The crystallographic ligand formed ten hydrogen bonds with ARG112 (1.74 Å), GLU209 (three bonds: 1.96 Å, 1.89 Å, 2.32 Å), THR107 (1.93 Å), ARG112 (2.00 Å), SER158 (2.19 Å), PRO105 (1.88 Å), THR159 (2.01 Å) and a C–H bond with GLY157 (3.49 Å). Accordingly, a hydrophobic interaction at LEU154 and two electrostatic interactions at TYR77 and GLU209 were also observed (Fig. 8c).

##### 3.2.5.6 Binding to human cyclin-dependent kinase 2 (PDB ID: 1pxo)

Kanzonol B exhibited excellent docking scores against human cyclin-dependent kinase 2, with a binding affinity of −10.04 kcal/mol. It was observed that kanzonol B formed four hydrogen bonds at PHE146 (3.19 Å), LEU143 (2.28 Å), GLU51 (1.87 Å), and ASP145 (3.15 Å) (Fig. 9a), as well as seven hydrophobic interactions at LEU134, ALA144, PHE80, VAL18, ALA31, LEU55, and VAL64.

In comparison, carbamazepine showed a lower binding affinity of −8.07 kcal/mol, forming only one H-bond with residue ASP145 (3.10 Å) (Fig. 9b). The crystallographic ligand extracted from the human cyclin-dependent kinase 2 protein model formed one H-bond with residue GLU51 (1.61 Å) (Fig. 9c).

##### 3.2.5.7 Binding to leucine-rich glioma inactivated 1 (LGI1) (PDB ID: 5y30)

Molecular docking of kanzonol B into the active site LGI1 showed a better affinity and a lower scoring function value (−8.07 kcal/mol) compared to carbamazepine (−6.06 kcal/mol). As illustrated in Fig. 10a, kanzonol B formed four H-bonds at ILE61 (1.59 Å), SER83 (2.06 Å and 2.05 Å) and GLU81 (2.20 Å), as well as one hydrophobic interaction at SER86. In contrast, carbamazepine formed three hydrophobic interactions at ILE61 (two bonds) and ARG63 respectively (Fig. 10b).

##### 3.2.5.8 Binding to human arginase I (PDB ID: 3thj)

As illustrated in Fig. 11a, kanzonol B demonstrated a strong binding to the human arginase I with a binding affinity of −6.78  kcal/mol, making it superior in binding efficiency as compared to carbamazepine (−5.74 kcal/mol). This high binding energy of kanzonol B in the binding site was attributed to the H-bonds formed with ASP128 (1.98Å), SER137 (1.92 Å), ASN139 (2.72 Å), HIS141 (2.52Å), GLU186 (1.78 Å), and ASP183 (2.03 Å), along with hydrophobic interactions at THR246.

Carbamazepine formed three hydrogen bonds at SER137 (2.26 Å), ASN139 (2.27 Å), and HIS141(2.01 Å) and an electrostatic interaction at ASP183. Furthermore, it displayed Pi-Lone Pair interaction with the residues ASP183 and SER137 (Fig. 11b). Meanwhile, the crystallographic ligand displayed eight salt bridges at ASP128 (2.14 Å, 1.93 Å, 2.01 Å and 2.45 Å), ASP124 (1.93 Å and 2.30 Å), ASP234 (3.06 Å), and ASP232 (1.86 Å); two C–H bonds at GLY142 (3.34 Å) and ASP234 (3.17 Å) (Fig. 11c). It was further stabilized by four electrostatic interactions at GLU186, GLU277, HIS126 and HIS14.

### 3.3 Drug likeness and ADMET prediction

Supplementary Table S7 shows the SMILES of all 63 phytoconstituents, which were used for ADMET property calculations. Supplementary Table S8 presents the key phytochemical parameters such as molecular weight (MW), logP, number of hydrogen bond acceptors and donors, number of rotatable bonds, and topological polar surface area (TPSA). Drug-likeness predictions result for the compounds, as displayed in supplementary Table S8, demonstrated that most of the compounds followed Lipinski’s rule and Veber's rule, and posed good oral bioavailability. Supplementary Table S9 presents the results for gastrointestinal (GI) absorption, BBB permeability, central nervous system (CNS) permeability, BA scores, and metabolism for the compounds studied. As displayed in supplementary Table S9, compounds NDFC7, NDFC14, PDFC4, and PDFC36 had high BBB permeation, which indicates these compounds are readily able to penetrate the BBB. Even though most of the studied compounds pass through the BBB, 27 out of 63 studied compounds from COFE were predicted to have poor brain diffusion. As shown in supplementary Table S9, most of the compounds have a BA score of 0.55 or 0.56, and CYP2C9 is involved in the metabolism of some of the studied compounds.

### 3.4 Boiled egg for GI absorption and brain penetration prediction

Supplementary Fig. S4 displays the BOLIED-Egg models for all the compounds studied. In the present study, 50 molecules out of 66 (including standard drugs) were within the prediction site. Compounds NDFC10 and 12, PDFC 4, 10, 11, 18, 20, 21, 24, 26–29, 32, 33, 36, 45, 46, and 49 were predicted to passively diffuse across the BBB and not be pumped out by p-glycoprotein efflux.

### 3.5 Toxicity prediction results

The phytocompounds of COFE were investigated for their toxicity using several toxicological endpoints, including rat acute toxicity, LD_50_ value, neurotoxicity, hepatotoxicity, cardiotoxicity, nephrotoxicity, carcinogenicity, immunotoxicity, mutagenicity, cytotoxicity, clinical toxicity, and their results are shown in supplementary Table S10. Many of the tested phytocompounds showed no activity for neurotoxicity, hepatotoxicity, cardiotoxicity, carcinogenicity, immunotoxicity, mutagenicity, and cytotoxicity.

### 3.6 Molecular dynamics studies

MD simulations lasting 100 ns were carried out on the top binding and scored complexes (ligand–protein complex) and standard drug-protein complex obtained from molecular docking studies in order to gain a better understanding of the stability of apo-protein, ligand–protein complex, and standard drug-protein complexes. The selected compound, kanzonol B, showed better stability when it was complexed with all the tested proteins in the current study.

#### 3.6.1 PDFC32- GABA-T complex

**Fig. 12:**
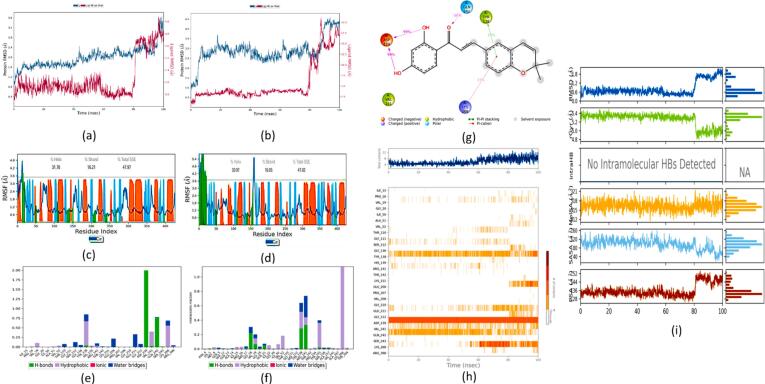
MD studies on gamma-aminobutyrate aminotransferase (GABA-T) complex with kanzonol B and carbamazepine: RMSD Plots GABA-T complex with **(a)** Kanzonol B, **(b)** Carbamazepine; RMSF plots for GABA-T with: **(c)** Kanzonol B, **(d)** Carbamazepine (Protein residues that interact with the ligand are marked with a green-colored vertical bar. The secondary structure elements (alpha-helices and beta-strands) of the protein are overlaid in the background of the simulation to show the structural context throughout the analysis); Interaction of the molecule with the protein target during simulation: **(e)** Kanzonol B, **(f)** Carbamazepine, **(g)** Kanzonol B interactions with the protein residues; **(h)** GABA-T protein residues contact with kanzonol B during 100 ns simulation (white refers to zero interaction while the deep color indicates more interactions); **(i)** Properties of kanzonol B with its interaction with GABA-T protein during a 100 ns MD simulation. (For interpretation of the references to color in this figure legend, the reader is referred to the web version of this article.)

The protein RMSD trajectory was represented in blue, the ligand RMSD trajectory was represented in red color, and the RMSD values were shown on the left Y-axis in Å units as shown in Fig. 12 (a) and (b). A comparative analysis of the RMSD trajectory between the kanzonol B-(GABA-T) complex (Fig. 12 (a)) and the carbamazepine-(GABA-T) complex (Fig. 12 (b)) suggested a lower RMSD value associated with the kanzonol B-(GABA-T) complex (1.969 ± 0.41 Å vs 2.878 ± 0.65 Å).

The protein RMSF plot of the kanzonol B-(GABA-T) complex is given in Fig. 12 (c), while the protein RMSF plot of the carbamazepine-(GABA-T) complex is shown in Fig. 12 (d). The green bars in the protein RMSF plot represent the amino acids interacting with the ligand. The plot in Fig. 12 (c) illustrates that the amino acid residues involved in kanzonol B-(GABA-T) complex are the same as in carbamazepine-(GABA-T) complex (Fig. 12 (d)).

The interaction chart between kanzonol B-(GABA-T) complex is given in Fig. 12 (e) and carbamazepine-(GABA-T) complex is given in Fig. 12 (f). The 2D interaction diagram showed that the complex was stabilized by hydrogen bonding, pi-cationic interaction, and hydrophobic interaction (Fig. 12 (g)). Fig. 12 (h) illustrates the changes in contacts over 100 ns of simulation time. The top panel shows the protein's total number of specific contacts with the kanzonol B. The bottom panel shows the interacting residues in each trajectory frame. A darker shade of orange represents residues that make more contact.

As illustrated in Fig. 12 (i), the RMSD concerning its atoms' initial positions fluctuated up to 5 ns of simulation time before reaching equilibrium at 0.8 Å and extended up to 80 ns. There was a sudden jump at 80 ns from 0.8 to 1.6 Å, and its stability was retained until the end of 100 ns. Compound kanzonol B showed an average rGyr value of 5.257 Å during the 100 ns MD simulation. Kanzonol B had no intramolecular hydrogen bonds. The molecular surface (MolSA) was calculated with a 1.4 Å probe radius, with this value being equivalent to the van der Waals surface area of a water molecule. Kanzonol B displayed better results with a lower average SASA of 95.70 Å^2^, higher average PSA of 135.89 Å^2^, and MolSA of 316.30 Å^2^ (Fig. 12 (i)).

#### 3.6.2 PDFC32- human mitochondrial branched chain aminotransferase (BCATm) complex

**Fig. 13:**
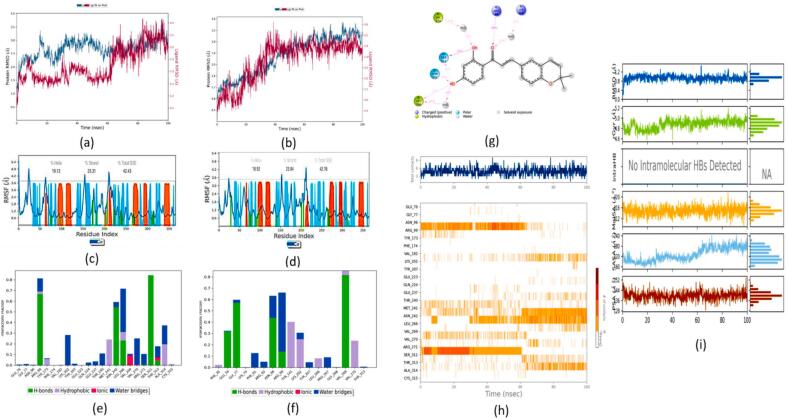
MD studies on Human mitochondrial branched chain aminotransferase (BCATm) complex with kanzonol B and carbamazepine: RMSD Plots BCATm complex with **(a)** Kanzonol B, **(b)** Carbamazepine; RMSF plots for BCATm with: **(c)** Kanzonol B, **(d)** Carbamazepine (Protein residues that interact with the ligand are marked with a green-colored vertical bar. The secondary structure elements (alpha-helices and beta-strands) of the protein are overlaid in the background of the simulation to show the structural context throughout the analysis); Interaction of the molecule with the protein target during simulation: **(e)** Kanzonol B, **(f)** Carbamazepine, **(g)** Kanzonol B interactions with the protein residues; **(h)** BCATm protein residues contact with kanzonol B during 100 ns simulation (white refers to zero interaction while the deep color indicates more interactions); **(i)** Properties of kanzonol B with its interaction with BCATm protein during a 100 ns MD simulation. (For interpretation of the references to color in this figure legend, the reader is referred to the web version of this article.)

A comparative analysis of the overall average RMSD plot between the kanzonol B-BCATm complex (Fig. 13 (a)) and the carbamazepine-BCATm complex (Fig. 13 (b)) suggested both ligands converge around comparable RMSD values. However, a lower RMSD value was associated with the carbamazepine-BCATm complex as compared to kanzonol B-BCATm complex (2.304 ± 0.69 Å vs 2.681 ± 0.37 Å).

Fig. 13 (c) illustrates the protein RMSF plot of the kanzonol B-BCATm complex, while the protein RMSF plot of the carbamazepine-BCATm complex is presented in Fig. 13 (d). In both figures, the green bars represent the amino acids interacting with the ligand. From Fig. 13 (c), it can be observed that the residues 36–57, 81–94, 109–129, 150–170, and 207–221 of BCATm protein displayed a few small fluctuations. The interaction chart between kanzonol B-BCATm complex is given in Fig. 13 (e), and carbamazepine-BCATm complex is given in Fig. 13 (f). The 2D interaction diagram suggested that the complex was stabilized by hydrogen bonding, pi-cationic interaction, and hydrophobic interaction (Fig. 13 (g)).

The changes in the contacts over 100 ns of simulation time are illustrated in Fig. 13 (h). At the beginning of the simulation, kanzonol B and BCATm proteins were initially distant from each other, resulting in a relatively lower number of contacts. However, as time progressed, the number of contacts increased, peaking between 40 to 100 ns.

As shown in Fig. 13 (i), the RMSD concerning its atoms' initial positions fluctuated up to 15 ns of simulation time, after which it reached equilibrium at around 0.9 Å and remained stable until 80.6 ns. At 80.6 ns, there was a sudden increase from 0.8 to 1.1 Å, and its stability was retained until the end of 100 ns. Kanzonol B showed an average rGyr value of 4.88 Å in the 100 ns MD simulation. Kanzonol B did not form any intramolecular hydrogen bonds. The MolSA was stable over most of the simulation time at around 314 Å^2^. Kanzonol B showed a higher average PSA of 139.775 Å^2^ and a MolSA of 314.852 Å.

#### 3.6.3 PDFC32- human voltage-gated sodium channel, brain isoform (Nav1.2) complex

**Fig. 14:**
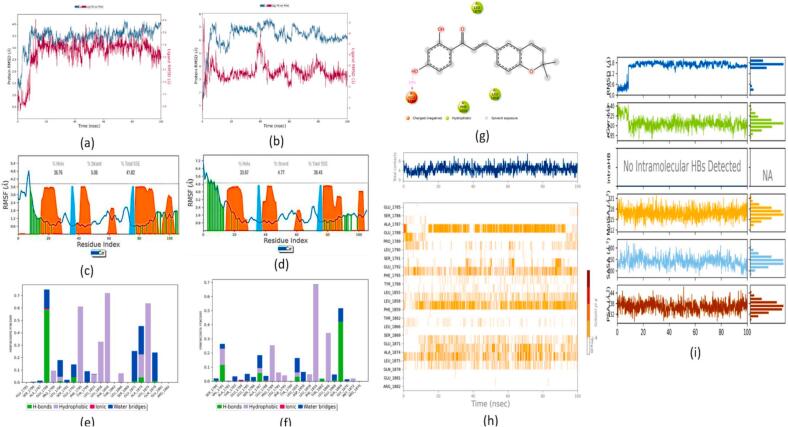
MD studies on Human Voltage-gated Sodium Channel, brain isoform (Nav1.2) complex with kanzonol B and carbamazepine: RMSD Plots Nav1.2 complex with **(a)** Kanzonol B, **(b)** Carbamazepine; RMSF plots for Nav1.2 with: **(c)** Kanzonol B, **(d)** Carbamazepine (Protein residues that interact with the ligand are marked with a green-colored vertical bar. The secondary structure elements (alpha-helices and beta-strands) of the protein are overlaid in the background of the simulation to show the structural context throughout the analysis); Interaction of the molecule with the protein target during simulation: **(e)** Kanzonol B, **(f)** Carbamazepine, **(g)** Kanzonol B interactions with the protein residues; **(h)** Nav1.2 protein residues contact with kanzonol B during 100 ns simulation (white refers to zero interaction while the deep color indicates more interactions); **(i)** Properties of kanzonol B with its interaction with Nav1.2 protein during a 100 ns MD simulation. (For interpretation of the references to color in this figure legend, the reader is referred to the web version of this article.)

A comparative analysis of the overall average RMSD plot between the HVGSC complex and the carbamazepine-HVGSC complex suggested a lower RMSD value was associated with the kanzonol B-HVGSC complex (Fig. 14 (a)) as compared to the carbamazepine-HVGSC complex (Fig. 14 (b)) (3.445 ± 0.46 Å vs 6.344 ± 0.88 Å).

The protein RMSF plot of the kanzonol B-HVGSC complex is illustrated in Fig. 14 (c), while the protein RMSF plot of the carbamazepine-HVGSC complex is presented in Fig. 14 (d). The green bars represent the amino acids interacting with the ligand in both figures. From Fig. 14 (c), it can be observed that the residues 5–7, 23–33, 54–60, and 66–77 of the human voltage-gated sodium channel protein displayed fluctuations. The interaction chart between the kanzonol B-HVGSC complex is shown in Fig. 14 (e), and the carbamazepine-HVGSC complex is given in Fig. 14 (f). The 2D interaction diagram suggested that the complex was stabilized by hydrogen bonding with GLU1788, GLU1792, ALA1874 (Fig. 14 (g)).

Fig. 14 (h) illustrates the changes in contacts over 100 ns of simulation time. The top panel shows the total number of specific contacts between the protein and kanzonol B, while the bottom panel shows the residues involved in the interactions in each trajectory frame. As shown in Fig. 14 (i), there was a sudden increase in the RMSD concerning its atoms' initial positions from 0.347 to 1.605 Å at 8.7 ns, before reaching a plateau with an average RMSD of 1.703 Å and its stability was retained until the end of 100 ns simulation. The average gyration radius (rGyr) value of kanzonol B was 5.13 Å during the 100 ns MD simulation. The MolSA was stable over most of the simulation time at around 316.80 Å^2^. The kanzonol B displayed better results with a high PSA of 136.25 Å^2^, and MolSA of 316.80 Å^2^, contributing to maintain the stability throughout the simulation.

#### 3.6.4 PDFC32- Leucine-rich glioma inactivated 1 (LGI1) complex

**Fig. 15:**
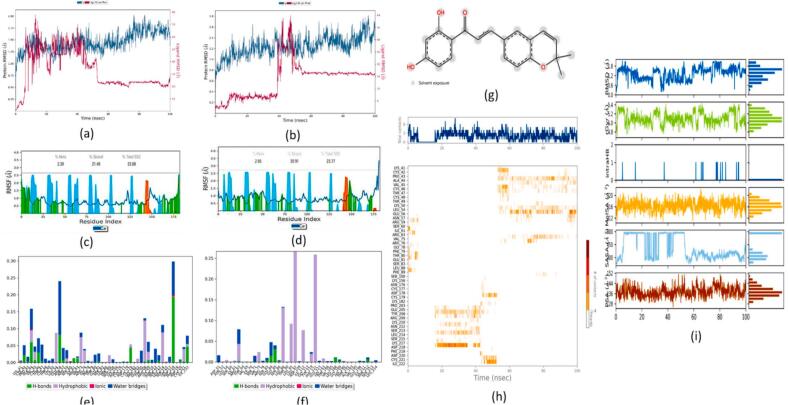
MD studies on Leucine-rich glioma inactivated 1 (LGI1) complex with kanzonol B and carbamazepine: RMSD Plots LGI1 complex with **(a)** Kanzonol B, **(b)** Carbamazepine; RMSF plots for LGI1 with: **(c)** Kanzonol B, **(d)** Carbamazepine (Protein residues that interact with the ligand are marked with a green-colored vertical bar. The secondary structure elements (alpha-helices and beta-strands) of the protein are overlaid in the background of the simulation to show the structural context throughout the analysis); Interaction of the molecule with the protein target during simulation: **(e)** Kanzonol B, **(f)** Carbamazepine, **(g)** Kanzonol B interactions with the protein residues; **(h)** LGI1 protein residues contact with kanzonol B during 100 ns simulation (white refers to zero interaction while the deep color indicates more interactions); **(i)** Properties of kanzonol B with its interaction with LGI1 protein during a 100 ns MD simulation. (For interpretation of the references to color in this figure legend, the reader is referred to the web version of this article.)

The average RMSD plot between the kanzonol B-LGI1 complex (Fig. 15 (a)) and the carbamazepine-LGI1 complex (Fig. 15 (b)) suggested a lower RMSD value was associated with the carbamazepine-LGI1 complex as compared to kanzonol B-LGI1 complex (1.238 ± 0.19 Å vs 1.454 ± 0.21 Å).

The protein RMSF plot of the kanzonol B-LGI1 complex is shown in Fig. 15 (c), while the protein RMSF plot of the carbamazepine-LGI1 complex is presented in Fig. 15 (d). In both figures, the green bars represent the amino acids that interact with the ligand. From Fig. 15 (c), the residues 25–33, 69–78, 86–100, 117–130, and 143–158 of LGI1 protein exhibited notable fluctuations. The interaction chart between kanzonol B-LGI1 complex is shown in Fig. 15 (e) and carbamazepine-LGI1 complex is given in Fig. 15 (f). The 2D interaction diagram showed that the kanzonol B had hydrogen bonding at ASP218, GLU56, ILE222, and hydrophobic interaction with TYR206, LEU214, LEU54, and VAL75 of LGI1 (Fig. 15 (g).

The changes in contacts over 100 ns simulation period are illustrated in Fig. 15 (h). At the beginning of the simulation, the kanzonol B and LGI1 were initially distant from each other, resulting in a lower number of contacts. Ligand properties, such as RMSD, rGyr, intramolecular hydrogen bond, MolSA, SASA, and PSA are presented in Fig. 15 (i). As illustrated in Fig. 15 (i), the RMSD concerning its atoms' initial positions had undergone fluctuations throughout the MD simulation. Kanzonol B displayed better results with a higher average PSA of 137.46 Å^2^, and MolSA of 316.34 Å^2^.

## 4 Discussion

Long-term consumption of polyphenol-rich foods confers numerous benefits, including protection against type 2 diabetes, cardiovascular and neurological illnesses, pancreatitis, osteoporosis, lung damage, cancer, and gastrointestinal disorders. Several studies have demonstrated the antiepileptic potential of phytochemicals. Polyphenols are characterized by their powerful antioxidant properties and have been reported to have the ability to mediate various signaling processes involved in epilepsy.^26^ Phytosterol has been reported for its antiepileptic ability through the GABAergic mechanism,^27^ and saponins have been identified as a potential bioactive agent in treating epilepsy. However, the exact mechanism of action of saponin remains unclear.^28^ Additionally, various types of alkaloids, including isoquinoline alkaloids,^29^ indole alkaloids,^30^ piperidine alkaloids,^31^ and aporphine alkaloids^32^ have also been demonstrated to exert antiepileptic effects.

Flavonoids have been found to show antiepileptic activities through diverse mechanisms, including the regulation of the GABAA-Cl-channel complex.^33^ Apart from aglycones, flavonoid glycosides have also demonstrated antiepileptic effects, such as vitexin^34^ and quercetin.^28^ Glycosides have been reported to exert a therapeutic effect on epilepsy, such as Paeoniflorin^35^ and iridoid glycoside.^36^ Furthermore, a comparative analysis has also revealed that cardiac glycosides have a different severity of anticonvulsant effect.^37^ Tannin has also been reported to be safe and desirable with the concurrent administration of Vitamin E in the treatment of epilepsy.^38^

It has been reported that the compounds found in the different species of plants have exhibited various pharmacological activities. For instance, hinokiflavone was reported to display multiple pharmacological activities, including anti-inflammatory, antioxidant, antiprotozoal, and antitumor activity.^39^ Besides, hinokiflavone has also shown its effectiveness against various types of cancerous disorders, including breast cancer, colorectal cancer, esophageal squamous cancer, adenocarcinoma, hepatocellular carcinoma, myeloid leukemia, and melanoma.^40^

Vitexin, found in *Ficus deltoidei*, demonstrated antioxidant, anti‐inflammatory, anticancer, antinociceptive, and neuroprotective effects.^41^ The protective effects of vitexin as an antioxidant against reactive oxygen species, lipid peroxidation, and other oxidative damages, including seizure, memory impairment, cerebral ischemia, neurotoxicity, myocardial and respiratory injury, and metabolic dysfunction, have been highlighted in many studies.^42^, ^43^ All these reported data provide scientific evidence and encourage us to extract the active phytoconstituents from *C. odontophyllum* fruits with the intention that they might be useful in the management of epilepsy.

The percentage yield of the hydro-alcoholic extract of *C. odontophyllum* fruits was 7.87 %. LC–MS Q-TOF was used to screen and identify bioactive chemicals from the hydro-alcoholic extract of *C. odontophyllum* fruits. A total of 63 phytocompounds have been found in the extract of *C. odontophyllum* fruits (Table 1, Supplementary Table S1 and S2). In that 49 compounds were detected in positive mode and 14 compounds in negative mode. In the positive ion mode, 49 compounds showed (M + H)^+^ ion peak, six compounds showed (M + NH_4_)^+^, and four compounds each had (M + Na)^+^ and (M + K)^+^ ion peaks. In the negative ion mode, 12 compounds showed (M−H)^-^ ion peaks, and one compound each had (M + CH_3_COO)^-^ and (M + Cl)^-^ ion peaks (Supplementary Table S2). The most common phenolic types identified are flavonoids and terpenoids, the other compounds are carbohydrates, lipids, and peptides. However, lipid compounds dominated the extract. The identified flavonoids are 3,5,7,2′,5′-pentahydroxyflavone, neovitexin, hinokiflavone, bavachromanol, kanzonol B, and robustaflavone. Abscisate and lamioside are the terpenoids, and ajmaline is an important alkaloid present in the *C. odontophyllum* fruit extract.

### 4.1 Molecular docking studies

The seven enzymes used in the current study were selected based on the following reasons. Gamma-aminobutyric acid (GABA) is a principal inhibitory neurotransmitter in the cerebral cortex, playing a pivotal role in counterbalancing neuronal excitation. Disruption of this balance may lead to seizures.^44^ GABA is produced in GABAergic axon terminals and released into the synapse, where it acts on GABA(A) receptors. These receptors are ligand-gated ionotropic chloride (Cl^-^) channels that mediate the majority of fast inhibitory neurotransmission in the adult CNS. When GABA binds to the GABAA receptor, it enhances the openings of the chloride ion channel, resulting in membrane hyperpolarization and a reduction of neuronal excitability, thereby suppressing the postsynaptic action potential generation.^45^ Natural compounds, particularly flavonoids and phenolic compounds, have been extensively investigated for their anxiolytic, sedative, and anticonvulsant properties, often attributed to their modulation of the GABA(A) receptor.^46^, ^47^ Several studies have reported that various plant-derived compounds interact with the benzodiazepine binding site of the GABA(A) receptor, influencing chloride ion influx and modulating neuronal excitability.^48^, ^49^ The role of GABA-T inhibition in the treatment of epilepsy is well-established, GABA-T is responsible for the catabolism of GABA, the primary inhibitory neurotransmitter in the CNS. Inhibiting GABA-T leads to increased GABA levels in the brain, thereby enhancing inhibitory neurotransmission and reducing neuronal hyperexcitability, which is characteristic of epileptic seizures.^50^, ^51^

BCATm is an enzyme that plays an important role in the catabolism of branched-chain amino acids (BCAAs), including leucine, isoleucine, and valine. It has been used as a target in several docking studies related to epilepsy.^52^, ^53^ BCATm is astrocyte-specific and it catalyzes the transfer of an amino group from BCAAs to α-ketoglutarate, removes α-ketoglutarate from the TCA cycle, and subsequently produces glutamate.^54^ The altered level of glutamate and its precursors impacts neuronal activity and potentially plays a role in seizure generation or modulation. The inhibition of BCATm can reduce the release of glutamate during excitation in neuronal tissues. Hence, the inhibition of glutamate release is useful for the treatment of epilepsy. The human voltage-gated sodium channel, brain isoform (Nav1.2) (HVGSC), encoded by SCN2A gene, is a major voltage-gated sodium channel in the CNS supporting action potential (AP) firing.^55^, ^56^ In epilepsy, mutations or dysfunctions in the SCN2A gene can alter the channel activity. HVGSC gain-of-function mutations increase neuronal excitability by allowing excessive sodium influx, leading to epilepsy.^57^ To address this issue, drugs can be designed to target HVGSC, correcting the channel's dysfunction by either inhibiting the excessive activity or modulating the channel’s behavior.

AMPA receptors (PDB ID: 3dp4) are ionotropic glutamate receptors, a major excitatory postsynaptic receptor mediating fast synaptic transmission by allowing the influx of sodium (Na+) ions into the postsynaptic neuron. It is widely distributed throughout the CNS, indicating its physiologic importance.^58^, ^59^ Studies have highlighted the pathophysiologic role of AMPA receptors in epilepsy.^60^, ^61^ Besides, AMPA-receptor antagonists have been found effective in reducing the severity of seizures and inhibiting the chemically induced seizures.^62^, ^63^, ^64^ The protein human cyclin dependent kinase 2 (PDB ID: 1pxo) was selected as it is involved indirectly in the epilepsy mechanism. CDK2 itself does not cause epilepsy directly, but is implicated in mechanisms that contribute to epilepsy, particularly in those with Alzheimer’s disease (AD). In AD, the accumulation of beta-amyloid (Aβ) will take place. Amyloid β1-42 is a major histological feature in AD. Accumulation of Aβ will form insoluble plaques that are toxic to neurons and may block cell-to-cell signaling at synapses. Aβ signaling through ROS production is uniquely mediated by the activation of PI3K/Akt. This pathway is essential for phosphorylating mammalian target of rapamycin complex 1 (mTORC1). Activation of mTORC1 by Aβ further increases the phosphorylation of eukaryotic translation initiation factor 4E (eIF4E), a binding protein (4E-BP1) and p70S6K1 to stimulate the HIF1α synthesis. This HIF1α synthesis further induced cyclinD /cyclin-dependent kinase 4 (CDK4) and cycline/CDK2, whereas it significantly attenuated the activation of autophagy.^65^ Several studies have proved that abnormal autophagy mechanisms can lead to epilepsy.^66^, ^67^ Besides, the presence of Aβ42 will further increase the neuronal excitability in AD, which subsequently initiates the development of progressive epilepsy.^68^ In short, CDK2 can be activated by pathways triggered by Aβ in AD, in turn, leading to neuronal excitability and potentially epilepsy.

Furthermore, research has shown that people with epilepsy, especially those with drug-resistant forms, may exhibit elevated levels of Aβ in their brains. Aβ will specifically induce the CDK2-mediated phosphorylation of tau, which is responsible for microtubule destabilization in promoting neuronal apoptosis. Apoptosis of neurons and glial cells in the brain is of great importance in the pathogenesis of epilepsy, especially the drug-resistant forms.^69^ A study on rats illustrated that epilepsy can lead to increased Aβ expression.^70^ Thus, it suggests a correlation between epilepsy, CDK2, and Aβ. Leucine-rich glioma inactivated 1 (LGI1) (PDB ID: 5y30) is a secreted neuronal protein, expressed predominantly in the hippocampus.^71^ LGI1 is involved in synaptic function and plasticity, and the development of neuronal networks. It also functions to modulate the neuronal excitability through the voltage-gated potassium Kv1.1 channels, and the postsynaptic ADAM22, which interacts with AMPA receptors.^72^, ^73^ The alteration of LGI1 functions can be seen in autosomal dominant temporal lobe epilepsy (ADLTE) and autoimmune limbic encephalitis, both characterized by epileptic seizures.^74^

Human arginase I (ARG1) (PDB ID: 3thj) was selected to be involved in this study as ARG1 can be indirectly involved in the epilepsy mechanism. ARG1 is the final enzyme that catalyzes the hydrolysis of arginine into ornithine and urea.^75^, ^76^ Arginine is also a substrate for nitric oxide synthase (NOS), which produces NO, a signaling molecule that is involved in neurotransmission and neuronal plasticity. Studies have shown that NO level is elevated during epileptic activity, suggesting a role in seizure activity.^77^ It remains unknown whether the increase in NO formation contributes to the development and/or maintenance of epileptic activity.^78^ However, the involvement of NO in seizure modulation through both proconvulsant and anticonvulsant capabilities has been highlighted, where this dual role for NO in seizure modulation depends on the epilepsy model and the drug dose used.^79^ Increased NO levels might enhance excitatory neurotransmission and synaptic activity, potentially lowering the threshold for seizures and increasing the frequency of epileptic events. By converting arginine into ornithine, ARG1 reduces the availability of arginine for NO production, potentially impacting NO levels and neuronal excitability. This reduction in NO potentially decreases excitatory neurotransmission, increases the seizure threshold, and thereby reduces the frequency or severity of seizures.^80^

#### 4.1.1 Protein preparation

All the used proteins except human cyclin dependent kinase 2 were used as downloaded from the PDB after curing. The protein human cyclin dependent kinase 2 downloaded from the PDB (1pxo) had few missing residues in between the head and tail residues. Hence, we modelled the human cyclin dependent kinase 2 using SWISS-MODEL. The RMSD between the original crystallographic protein and the modelled protein was 1.305 Å, indicating a high degree of similarity between these two proteins since the RMSD was below 2 Å.

#### 4.1.2 Protein validation

The Ramachandran plot is a representation of phi (ö) and psi (ø) angles of amino acid residues, providing information about the total number of amino acid residues in the favored, allowed, and disallowed regions. For a model to be considered high quality and close to the native structure, at least 90 % of the torsion angles of each amino acid residue must reside should lie within the favored region of the plot.^81^, ^82^ Supplementary Fig. S3 shows that more than 90 % of the amino acid residues were in the most favored and additionally allowed regions, suggesting that the model is reliable, stable, and of good quality. The overall G-factor of >−0.5 indicates good model quality. ^83^

ERRAT evaluates the quality of protein models by analyzing the statistics of non-bonded interactions between different atom types, where higher scores indicate better quality.^84^ According to this parameter, a score of 95 % or higher indicates a good high-resolution model.^83^ However, a quality factor of > 50 % is generally accepted as indicative of a good quality model.^85^, ^86^ In the current study, the modeled human cyclin-dependent kinase 2 achieved an ERRAT value above 90 %. A model Z-score obtained within the range typically found for native proteins of similar size indicates high quality model, while a score outside this range indicates structural error.^87^ According to the ProSA-web plot, the Z-scores for PDB protein structures with 300–500 residues are in the range of −1 to −13.^88^ The modeled protein fell within this range, as shown in the supplementary Fig. S3. Local model quality assessment with a lower energy level than zero further supports the reliability of this protein model.

The predicted LG score with a value >6 also falls within the acceptable range for an extremely good model.^85^ However, the modeled protein showed moderate verify3D score, which remained comparable with the original protein score. The modeled protein passed the validation criteria, confirming their overall good quality and suitability for the molecular docking experiments.

#### 4.1.3 Docking protocol validation

The RMSD value accurately predicts the ligand’s geometry, orientation, and position relative to its reference native pose.^89^ The smaller the RMSD value, the closer the ligand position is to the natural ligand conformation. RMSD value less than 2 Å is deemed very good, indicating a valid docking protocol, and suitable for the docking process.^90^ While an RMSD between 2 Å and 3 Å is considered acceptable. Conversely, an RMSD exceeding 3 Å is considered suboptimal.^91^ A cut-off value of RMSD <2 Å is generally recognized as the most effective threshold for validating accurately posed molecules.^92^, ^93^ The RMSD values shown in Table 2 confirm that the docking protocol was successfully validated and can be used to dock the phytocompounds against the selected proteins with specificity and accuracy.

#### 4.1.4 Virtual screening

Ligands with a lower negative docking score were selected as these indicate a stronger binding affinity between the receptor and ligand molecules.^94^ Based on the virtual screening result (Supplementary Table S5), kanzonol B was selected as the best binding compound based on its binding score, binding interactions, and diversity. Although human arginase I and GABA(A) receptor showed stronger binding affinity for 19-norandrosterone (PDFC27), this compound may be a contaminant or a rare metabolite from phytosterol by microbial action. Hence, kanzonol B was also chosen for these two proteins, since it showed comparable binding affinity against these proteins. Consequently, kanzonol B was further subjected to individual molecular docking analysis using AutoDock 4.2.6 to investigate its potential as an antiepileptic candidate.

#### 4.1.5 Docking studies

##### 4.1.5.1 Binding to Gamma-Aminobutyric acid GABA(A) receptor (PDB ID: 1gnu)

Docking results indicate that the significant binding affinity of kanzonol B (−6.70 kcal/mol), being comparable to, and even slightly better than that of diazepam (−6.63 kcal/mol), is a promising finding. The formation of H-bonds with LYS 66 and HIS69 by kanzonol B suggests critical polar interactions that may significantly contribute to its binding stability. While these specific residues are not universally reported in the literature for all GABA(A) receptor modulators, the involvement of amino acid residues in the vicinity of the benzodiazepine binding site is a common feature for potent modulators. For example, residues like HIS101, TYR159, and PHE162 on the α subunit and GLY200 on the γ subunit are often implicated in benzodiazepine binding within the α+/γ − interface, a well-characterized benzodiazepine binding site.^95^, ^96^ Although our current study interacting residue (LYS 66, HIS69, ARG65, ARG71) might differ from some published conventions (which can vary based on the specific receptor subunit and numbering scheme used in the PDB file), the general principle of a combination of hydrogen bonding and hydrophobic interactions driving ligand binding remains consistent with established literature on GABA(A) receptor modulation. Its comparable binding affinity to diazepam suggests its potential as a novel lead compound for developing new therapeutic agents targeting GABA(A) receptor-related disorders.

##### 4.1.5.2 Binding to gamma-aminobutyrate aminotransferase (GABA-T) (PDB ID:1sff)

The molecular docking study revealed significant interactions between kanzonol B and the enzyme GABA-T, demonstrating its potential as an antiepileptic agent. The impressive binding affinity of kanzonol B (−10.65 kcal/mol) to GABA-T, and this strong interaction, characterized by multiple hydrogen bonds involving residues such as SER112, GLU211, and ASP239, suggests a robust binding pose that could effectively inhibit enzyme activity. The involvement of GLU211 and ASP239, which are also crucial for the binding of the crystallographic ligand, indicates that kanzonol B interacts with key catalytic or binding residues within the GABA-T active site. Previous studies on GABA-T inhibitors have highlighted the importance of interactions within the active site pocket, often involving similar types of polar and hydrophobic contacts, such as those observed with vigabatrin, a known irreversible GABA-T inhibitor.^97^

The finding that the crystallographic ligand exhibits the strongest binding and a greater number of hydrogen bonds, including a salt bridge with GLU211, validates our docking methodology and highlights the optimal interactions for GABA-T inhibition. The fact that kanzonol B shares several common interacting residues with the crystallographic ligand (e.g., GLU211, SER112, ASP239, VAL241), despite forming fewer total bonds than the native ligand, is indicative of a similar binding pocket engagement and a promising inhibitory mechanism. The higher binding affinity and the nature of interactions displayed by kanzonol B compared to carbamazepine strongly support its potential as a more potent GABA-T inhibitor.

This study provides preliminary evidence that kanzonol B may serve as a novel antiepileptic agent by modulating GABA-T activity. Its superior binding affinity and favourable interaction profile with key active site residues of GABA-T, when compared to carbamazepine, warrant further experimental validation to confirm its inhibitory effect on the enzyme and its antiepileptic potential *in vitro* and *in vivo*.

##### 4.1.5.3 Binding to human mitochondrial branched chain aminotransferase (BCATm) (PDB ID:2a1h)

Although carbamazepine is an established antiepileptic drug, it primarily exerts its effects by blocking voltage-gated sodium channels.^98^ Its interaction with BCATm, as observed in our study, might be a secondary or off-target, possibly contributing to a multi-target mechanism of action. The fact that kanzonol B displays a higher binding affinity suggests it could be a more potent or selective modulator of BCATm activity. The specific hydrogen bonding and hydrophobic interactions observed for kanzonol B with residues including ARG99, SER311, GLY77, GLY312, ALA314, MET241, and VAL270 are crucial. Previous studies on BCATm inhibitors have emphazised the importance of interactions with residues in the active site, which typically accommodates the branched-chain α-keto acids and glutamate/α-ketoglutarate substrates. For instance, the active site of BCATm is known to involve residues that interact with carboxylate and amino groups of its substrates. The involvement of ARG99, which forms hydrogen bonds and electrostatic interactions with both kanzonol B and the crystallographic ligand, suggests its critical role in ligand binding within the BCATm active site. ARG99 is frequently reported to be involved in substrate recognition and catalysis in aminotransferases due to its positive charge.^99^ The presence of multiple hydrogen bonds and hydrophobic interactions collectively contributes to the stability of the kanzonol B-BCATm complex.

The crystallographic ligand displayed strong binding with numerous hydrogen bonds, including salt bridges at ARG99 and LYS202, validates the active site's capacity for highly stable interactions. The fact that kanzonol B shares common interacting residues with the crystallographic ligand, such as ARG99 and SER311, further supports the notion that kanzonol B effectively docks into the relevant binding pocket of BCATm. While the crystallographic ligand shows a higher number of interactions and stronger types of bonds (e.g., salt bridges), the observed interactions for kanzonol B are still robust and indicate a promising inhibitory potential.

The superior binding affinity and interaction profile of kanzonol B with BCATm, compared to carbamazepine, suggest that it could serve as a novel lead compound for developing BCATm inhibitors. Given the emerging role of BCATm in neurological disorders, further experimental validation of kanzonol B's inhibitory activity against BCATm and its potential antiepileptic effects *in vitro* and *in vivo* is warranted.

##### 4.1.5.4 Binding to human voltage-gated sodium channel, brain isoform (Nav1.2) (PDB ID: 2kav)

The docking study results indicate that kanzonol B forms stronger molecular interactions and a more stabilized protein–ligand complex with the HVGSC, as evidenced by its higher binding energy compared to carbamazepine. This suggests that kanzonol B possesses potential antiepileptic effects by modulating channel function and reducing excessive neuronal firing. Our findings align with the established role of VGSCs as a primary target for AEDs. The observed binding affinity of carbamazepine to the HVGSC (−6.63 kcal/mol) is consistent with its known efficacy as a sodium channel blocker. Numerous studies have detailed the interactions of carbamazepine and similar AEDs with key residues in the pore-forming regions or voltage-sensing domains of VGSCs, often involving hydrophobic interactions and hydrogen bonds.^100^ The specific residues involved, such as SER1869, VAL1865, and GLU1868, are commonly found in the drug-binding sites of VGSCs, particularly within the domain IV S6 segment, which is a well-known binding locus for many sodium channel blockers.^101^ The PDB ID 2KAV corresponds to a pore-forming module of a voltage-gated sodium channel, making it a relevant model for studying drug interactions.

Natural compounds, particularly those with flavonoid or phenolic structures like kanzonol B, have been increasingly investigated for their neuroactive properties, including interactions with ion channels. While specific studies on kanzonol B's interaction with VGSCs might be limited in the literature, the general concept of plant-derived compounds modulating these channels is well-documented.^102^

The stronger molecular interactions and more stabilized protein–ligand complex observed for kanzonol B suggest that it could be a potent modulator of HVGSCs, potentially by stabilizing the inactivated state of the channel, similar to carbamazepine, but with enhanced efficacy. This would lead to a reduction in repetitive neuronal firing, a crucial mechanism for antiepileptic drugs.^103^ Further experimental validation using electrophysiological studies is essential to confirm the functional effects of kanzonol B on HVGSCs and to elucidate its precise mechanism of action (e.g., state-dependent blockade, frequency-dependent blockade). These findings position kanzonol B as a promising lead compound for the development of novel antiepileptic agents targeting voltage-gated sodium channels.

##### 4.1.5.5 Binding to α-amino-3-hydroxy-5-methyl-4-isoxazolepropionic acid (AMPA) (PDB ID: 3dp4)

Overall, kanzonol B displayed a weaker binding affinity than the crystallographic ligand but had a stronger binding and a greater number of more robust hydrogen bonds than carbamazepine. These results suggest that kanzonol B might be beneficial in epilepsy treatment by potentially blocking the AMPA receptor and inducing membrane hyperpolarization. Our findings are consistent with the understanding that AMPA receptor antagonism can be a viable approach for antiepileptic effects. The binding of the crystallographic ligand, showing numerous hydrogen bonds and electrostatic interactions with key residues like ARG112, GLU209, and THR107, highlights the critical contact points within the AMPA receptor's ligand-binding domain. These residues are known to be crucial for agonist binding and subsequent channel activation.^104^, ^105^ The formation of salt bridges at ARG112 and strong conventional hydrogen bonds by the crystallographic ligand underscores the high-affinity nature of physiological ligand interactions.

Carbamazepine's binding to the AMPA receptor, although weaker than kanzonol B, is of interest. While carbamazepine's primary mechanism of action is voltage-gated sodium channel blockade, some studies have suggested potential modulation of glutamatergic systems by AEDs, including AMPA receptor interactions, which could contribute to their overall antiepileptic effects.^72^ The observed interactions with GLU209 and LEU154 by carbamazepine align with known regions involved in ligand binding within the AMPA receptor.

The superior binding affinity of kanzonol B (−8.30 kcal/mol) compared to carbamazepine (−6.79 kcal/mol) for the AMPA receptor is a significant finding. Kanzonol B's ability to form four hydrogen bonds with ARG96 and ARG64, along with hydrophobic interactions, suggests a strong and specific interaction with the receptor. ARG residues in the ligand-binding domain are often critical for interacting with the acidic groups of glutamate or its analogs.^106^ The greater number and strength of hydrogen bonds formed by kanzonol B, relative to carbamazepine, imply a more stable and potentially effective blockade of the AMPA receptor.

Natural compounds, including flavonoids and other plants secondary metabolites, have been increasingly explored for their neuroprotective and anticonvulsant properties, often attributed to their ability to modulate ion channels and neurotransmitter receptors, including AMPA receptors.^49^, ^107^ The robust interaction of kanzonol B with the AMPA receptor positions it as a promising candidate for further investigation as a novel antiepileptic agent. If kanzonol B indeed acts as an AMPA receptor antagonist, it could lead to membrane hyperpolarization by reducing excitatory input, thereby dampening neuronal excitability and preventing seizure activity.^108^, ^109^ Further experimental studies, including electrophysiological assays, are crucial to confirm the antagonistic properties of kanzonol B on AMPA receptors and to evaluate its efficacy in preclinical models of epilepsy.

##### 4.1.5.6 Binding to human cyclin-dependent kinase 2 (modeled)

Our finding that kanzonol B exhibits an exceptionally high binding affinity for human CDK2 (−10.04 kcal/mol) is particularly striking. This score is significantly more favorable than that of carbamazepine (−8.07 kcal/mol), which is primarily known for its voltage-gated sodium channel blocking activity and not typically considered a direct CDK2 inhibitor.^98^ The observed single hydrogen bond for carbamazepine with ASP145, while present, does not suggest a strong or specific interaction with the CDK2 active site. Similarly, the crystallographic ligand, which often represents a fragment or a tightly bound physiological substrate/inhibitor, also shows only one hydrogen bond with GLU51. This could indicate a highly specific interaction or that the fragment itself has limited contact points.

Kanzonol B's interaction profile with CDK2, characterized by four strong hydrogen bonds involving PHE146, LEU143, GLU51, and ASP145, along with seven hydrophobic interactions, suggests a robust and multifaceted binding mechanism. The residues PHE146 and LEU143 are in the DFG motif (Asp-Phe-Gly) and activation loop of CDK2, respectively, which are critical for kinase activity and ATP binding.^110^, ^111^ GLU51 and ASP145 are also highly conserved residues in the active site of CDKs, often involved in hydrogen bonding with ATP or ATP-mimetic inhibitors. Extensive hydrophobic interactions further contribute to the stability of the kanzonol B-CDK2 complex.

The superior binding affinity and more extensive interaction network of kanzonol B compared to carbamazepine strongly suggest that kanzonol B could be a potent inhibitor or modulator of CDK2. While its direct relevance to antiepileptic effects via CDK2 modulation requires further experimental validation, the implication of CDK dysregulation in neuronal network hyperexcitability and epilepsy warrants investigation into this potential target.^112^ For instance, specific CDK inhibitors have shown neuroprotective effects in various neurological disease models by modulating neuronal survival and synaptic plasticity.^113^

This study, therefore, identifies kanzonol B as a promising compound with high affinity for human CDK2, surpassing the binding of a common antiepileptic drug, carbamazepine. Further experimental studies are essential to confirm the inhibitory effect of kanzonol B on CDK2 activity and to explore its functional consequences in neuronal systems and its potential as a novel therapeutic agent for epilepsy or other neurological conditions where CDK2 dysregulation plays a role.

##### 4.1.5.7 Binding to leucine-rich glioma inactivated 1 (LGI1) (PDB ID: 5y30)

Our finding showed that kanzonol B exhibits a significantly higher binding affinity for LGI1 (−8.07 kcal/mol) compared to carbamazepine (−6.06 kcal/mol) is particularly noteworthy. While carbamazepine is effective in treating ADLTE, its primary mechanism of action is well-established as voltage-gated sodium channel blockade. Its interaction with LGI1, as observed in our study, might represent a secondary or off-target interaction, or it could contribute to its overall therapeutic efficacy in ADLTE. The relatively weak and purely hydrophobic interactions for carbamazepine suggest that it may not directly modulate LGI1 function in a specific or potent manner.

In contrast, kanzonol B's robust binding with four hydrogen bonds involving ILE61, SER83, and GLU81, along with a hydrophobic interaction at SER86, indicates a strong and specific engagement with LGI1. The residues ILE61, SER83, and GLU81 are likely located within or near functional domains of LGI1 that are critical for its interactions with ADAM proteins or other synaptic components. For example, the epilepsy-associated mutations in LGI1 often cluster in the LRR (leucine-rich repeat) domains or the EPTP repeats, which mediate protein–protein interactions.^114^ Therefore, compounds that can bind to these regions and modulate LGI1′s interaction with its partners could have therapeutic implications.

The superior binding affinity and the nature of interactions (multiple hydrogen bonds versus only hydrophobic) demonstrated by kanzonol B suggest a more favourable and specific binding to LGI1 compared to carbamazepine. This indicates that kanzonol B has a greater potential to directly modulate LGI1 function, potentially by stabilizing its active conformation, promoting its secretion, or enhancing its interaction with ADAM receptors, thereby restoring proper synaptic transmission and reducing neuronal excitability.^115^

Given the strong association between LGI1 dysfunction and epilepsy, the identification of kanzonol B as a potent LGI1 ligand opens a new avenue for antiepileptic drug development. Further experimental studies are crucial to validate the functional effects of Kanzonol B on LGI1 in cellular and animal models of epilepsy, particularly those linked to LGI1 dysfunction, and to determine its precise mechanism of action in modulating LGI1-mediated synaptic transmission.

##### 4.1.5.8 Binding to human arginase i (PDB ID: 3thj)

The extensive hydrogen bonding pattern observed for kanzonol B with ARG21, THR127, and ASP128 highlights its specific interaction with the ARG1 active site. ARG1 is a metalloenzyme requiring manganese ions for its catalytic activity, and residues like ASP128 are known to be critical for coordinating these metal ions and interacting with the substrate.^116^ The multiple salt bridges formed by the crystallographic ligand with aspartate residues (ASP128, ASP124, ASP234, ASP232) further emphasize the importance of these negatively charged residues in the active site for optimal ligand binding. The high number of salt bridges in the crystallographic ligand complex suggests that this native or tightly bound ligand forms strong ionic interactions with the enzyme's active site, which is typical for highly specific enzymatic reactions. Kanzonol B, by forming hydrogen bonds with some of these critical residues, appears to effectively engage with the ARG1 active site.

The proposed mechanism that kanzonol B activates ARG1, thereby decreasing arginine availability and subsequently reducing NO production by NOS, aligns with the hypothesis that modulating the L-arginine-NO pathway can influence seizure threshold. Elevated NO levels have been implicated in seizure generation and progression in various experimental models of epilepsy.^117^ By activating ARG1, kanzonol B could enhance the catabolism of L-arginine, thus limiting its availability for NOS, and ultimately reducing NO-mediated excitatory neurotransmission.^118^ This mechanism suggests a novel pathway through which kanzonol B could exert its antiepileptic effects, distinct from the classical targets of conventional AEDs.

The comprehensive molecular docking study, encompassing interactions with GABA(A) receptor, GABA-T, BCATm, HVGSC, AMPA receptor, CDK2, LGI1, and now ARG1, strongly suggests that kanzonol B is a multi-targeted compound with significant potential for antiepileptic activity. The consistently strong binding affinities and favourable interaction profiles across these diverse targets, particularly when compared to carbamazepine, point towards a multifactorial mechanism of action for kanzonol B in epilepsy treatment. This multi-target approach could offer advantages in treating complex neurological conditions like epilepsy, potentially leading to broader efficacy and reduced drug resistance. Future experimental studies are crucial to validate these predicted biological activities and to elucidate the precise functional consequences of kanzonol B's interactions with each identified target.

### 4.2 Drug likeness and ADMET prediction

The values of MW, TPSA and the LogP are indications of good membrane permeability and oral bioavailability of the compounds, while the number of rotatable bonds accounts for the good intestinal availability.^119^ Lipinski’s rule is known as the rule of five (RO5).^120^ It is used to assess whether a chemical compound has properties that are consistent with being an orally active drug. The Lipinski’s rule suggests that a compound is likely to have poor absorption or permeation if its molecular weight is greater than 500 Da, lipophilicity (LogP) and hydrogen-bond donors (HBD) are more than 5, and the hydrogen-bond acceptors are more than 10.^121^, ^122^ Veber's rule (VR) further refines the criteria for bioavailability, with two simple rules (total polar surface area (TPSA) ≤ 140 Å^2^, rotatable bonds ≤ 10) that compounds should adhere to for optimized bioavailability.^123^

The results of drug-likeness predictions for the compounds, as displayed in supplementary Table S8, showed that most of the compounds followed Lipinski’s rule and Veber's rule, posing good oral bioavailability. The violations of more than one Ro5 parameter depict that the molecule was not orally active, having poor permeability or absorption.^119^, ^124^ All the studied compounds obeyed Lipinski’s rule, except for NDFC3, NDFC8, PDFC1, PDFC9, PDFC30, PDFC39 and PDFC43, with more than one violation, suggesting they have a potential bioavailability problem. While NDFC9, NDFC13, PDFC2, PDFC4, PDFC5, PDFC6, PDFC7, PDFC8, PDFC10, PDFC11, PDFC12, PDFC14, PDFC21, PDFC27, PDFC31, PDFC32, PDFC36, PDFC37, PDFC38 AND PDFC40 followed Veber’s rule with no violation. The remaining compounds displayed a violation of Veber’s rule, predicted to have poor oral bioavailability. Overall, most of the compounds were expected to exhibit good oral bioavailability. The selected compounds from COFE for docking studies, kanzonol B demonstrated high GI absorption. Kanzonol B exhibits good absorbance in the human intestine, like that of established drugs such as carbamazepine, diazepam and vigabatrin.

For blood–brain barrier (BBB) permeation, compounds with logBB values ≥ 0.3 are readily permeable through the BBB. While compounds with logBB values between 0.3 < logBB <  − 1 can still pass the BBB. While compounds having logBB values <  − 1 are poorly diffused into the brain.^125^ As displayed in supplementary Table S9, NDFC7, NDFC14, PDFC4 and PDFC36 compounds hold the high BBB permeation, readily to penetrate the BBB. Most of them pass the BBB, while 27 compounds out of 63 studied compounds from COFE are predicted to have poor brain diffusion. For the CNS permeability interpretation, compounds with a logPS > −2 are considered to penetrate the CNS, whereas those with logPS < −3 are considered unable to penetrate the CNS. PDFC32 has high CNS permeability, and can penetrate the BBB, resulting in the ability to bypass drug-hindering barriers and potentially exert therapeutic activity against neurological disorders.

A bioavailability score (BA) of 0.55 or 0.56 suggests that the compounds are ideal and are absorbed well by the body.^126^, ^127^ As shown in supplementary Table S9, most of the compounds have a BA score of 0.55 or 0.56, indicating good pharmacokinetic properties. Metabolism involves the chemical biotransformation of a drug by the body. Cytochrome P450 is a superfamily of membrane-bound hemoprotein isozymes with distinct classifications. Although present in various tissues, CYP enzymes are most concentrated in the liver, intestines, and kidneys, with six isoenzymes (CYP1A2, CYP2C9, CYP2C19, CYP2D6, CYP2E1, and CYP3A4) responsible for 90 % of drug metabolism.^128^ In particular, CYP2C9 has been reported to be involved in the metabolism of an anti-epileptic agent, phenytoin and an anticoagulant, warfarin.^129^ Supplementary Table S9 revealed that CYP2C9 is involved in the metabolism of kanzonol B.

### 4.3 Boiled-egg for GI absorption and brain penetration prediction

The BOILED-egg model is used to assess the capability of GI absorption and permeability of the BBB.^130^ In the BOILED-Egg model, the white region indicates a high probability for passive absorption by the GI tract, and the yellow region (yolk) indicates a high probability of brain penetration. In addition, the points are colored in blue if predicted as actively effluxed by P-gp (PGP + ) and in red if predicted as non-substrate of P-gp (PGP-).^131^ The selected compounds from COFE for docking studies, kanzonol B were located within the yolk of the boiled egg, suggesting that this compound has high brain penetration and may cause mild CNS adverse effects. Kanzonol B are not subject to active efflux (red dot).

### 4.4 Toxicity prediction results

For oral rat acute toxicity, a substance with a lower LD_50_ is more lethal than a substance with a higher LD_50_. The c classification system categorizes toxicity into several classes based on LD_50_ values in mg/kg, with Class I: fatal if swallowed (LD_50_ ≤ 5), Class II: fatal if swallowed (5 < LD_50_ ≤ 50), Class III: toxic if swallowed (50 < LD_50_ ≤ 300), Class IV: harmful if swallowed (300 < LD_50_ ≤ 2000), Class V: may be harmful if swallowed (2000 < LD_50_ ≤ 5000) and Class VI: non-toxic (LD_50_ > 5000). Most of the compounds studied were in Class IV, V and VI. To highlight, the selected compounds for molecular docking and dynamic studies (kanzonolB) are in Class V, indicating that they are safe.

Previous studies have shown that the long-term use of AEDs can cause nervous system damage.^132^ Short-term uses of AEDs can cause neurons damage in the immature brain, and the combined use of antiepileptic drugs exacerbates the damage.^133^ Given that *C. odontophyllum* fruits are targeting epilepsy, it is essential to assess the potential neurotoxic risks of COFE. The results show that most of the compounds from COFE do not exhibit neurotoxicity, except for NDFC9, PDFC8, PDFC27, PDFC38 and PDFC40.

Long-term use of AEDs is associated with renal issues, particularly the deleterious effects of some AEDs on the kidney.^134^ The disposition of AEDs can be altered in patients with impaired renal function, leading to a higher risk of AED toxicity or therapy failure.^135^ Thus, nephrotoxicity is also one of the parameters to be considered. Among the studied compounds from COFE, more than 50 % of compounds are identified to be potentially causing nephrotoxicity, suggesting renal dosage adjustment and close monitoring may be necessary when using COFE in epilepsy management. In addition, most of the compounds from COFE were predicted as not exhibiting hepatotoxicity, cardiotoxicity, carcinogenicity, immunotoxicity, mutagenicity, cytotoxicity, and clinical toxicity.

### 4.5 Molecular dynamics studies

#### 4.5.1 PDFC32- GABA-T complex

RMSD is an essential measure for analyzing the MD trajectory equilibration and checking the stability of complicated systems throughout the simulation process. RMSD demonstrates the conformational changes occurring in the protein’s backbone over the simulation.

The RMSD of the kanzonol B-(GABA-T) complex and the carbamazepine-(GABA-T) complex suggested kanzonol B-(GABA-T) complex is more stable than the standard drug-protein complex (Fig. 12 (a), (b)). While protein in kanzonol B-protein complex reached equilibrium at 20 ns and plateaued up to 47 ns, and then there was a small jump in RMSD at 48 ns, and then it showed stabilized equilibrium until 80 ns with an average RMSD of 1.8 Å. Moreover, at around 81 ns, the RMSD values observed for the protein in the complex were significantly larger, indicating the possibility that the ligand had a different binding mode away from its initial binding site or major rearrangement of the molecule (Fig. 12 (a)). Similarly, around 78.8 ns, the RMSD values observed for the carbamazepine-(GABA-T) complex were significantly larger, which indicated a significant possibility for shifting out of carbamazepine from the GABA-T active pocket (Fig. 12 (b)). RMSD of kanzonol B was stable in the complex, and the average RMSD was found to be 1.793 ± 1.316 Å. There was a sudden jump at 80 ns from 0.8 to 4.5 Å, and its stability was retained until the end at 4.4 Å. This sudden jump indicated that there might be a conformational change in the molecule (Fig. 12 (a)). However, the standard carbamazepine showed a high average RMSD with 5.571 ± 4.672 Å in the molecular dynamic trajectory (Fig. 12 (b)). The result indicates that GABA-T has periodic backbone stability throughout the MD simulation while bound with the kanzonol B.

RMSF measures how far atomic locations have deviated from the initial point. RMSF, in other words, demonstrates the dynamic nature of protein–ligand interaction. Both figures (Fig. 12 (c) and (d)) illustrate that the N-terminal amino acid residues have a high RMSF value because these residues are free to move and highly reactive.^136^ It was observed from Fig. 12 (c) that a few small fluctuations in scale for the residues 69–80, 153–160, 172–177, 283–296, 359–364 of GABA-T protein, and this flexibility is due to these domains not possessing ligands. The plot in Fig. 12 (c) showed that the amino acid residues of the protein behaved like carbamazepine-GABA-T (Fig. 12 (d)) during simulation. All the fluctuations were the same as the reference, and the overall plots showed similar trends. Interestingly, the binding of kanzonol B had slightly reduced fluctuations than carbamazepine with GABA-T. Even though the intermolecular interactions produce low fluctuation, the secondary structure elements (alpha helices and beta strands) make the protein molecule slightly rigid. Protein secondary structure elements (SSE) like alpha-helices and beta-strands are monitored throughout the simulation and shown in Fig. 12 (c) and (d).

Simulation interaction diagrams during the entire MD run provided insights into the interaction pattern of the ligands with GABA-T protein. The X-axis on the plot shows the names of residues interacting with the ligand, and the Y-axis on the plot shows the bond fraction. Fig. 12 (e) indicates that the kanzonol B complex with GABA-T was stabilized by hydrogen bonding with ASP239 and GLN242, pi-cationic interaction with LYS268, and hydrophobic interaction with TYR138 and VAL241. Interestingly, few water bridges were observed involving SER112, TYR138, GLU211, ARG141, and SER268, and the residue TYR138 has also formed pi–pi stacking interaction with the ligand. Further experimental investigation is needed to confirm the significance of water and the interactions. For the conventional hydrogen bonds, ASP239 showed the highest interaction fraction around 100 % with 2nd and 4th –OH groups of kanzonol B, followed by GLN242 at around 66 % with −C=O of ligand (Fig. 12 (g)). The same types of hydrogen bond interactions had also been observed in our molecular docking studies. A higher value of interaction fraction suggested that the hydrogen bonding interaction was adequately maintained during the 100 ns MD simulation.

Fig. 12 (h) illustrates that a darker shade of orange represents residues that make more contact with the ligand. At the beginning of the simulation, the compound kanzonol B and GABA-T protein were initially distant from each other, and thereby, the number of contacts was relatively low. As time progressed, the interactions increased, leading to a rise in the number of contacts. Between 60 to 100 ns, the number of contacts reached its maximum value, indicating a substantial level of interaction between kanzonol B and GABA-T protein. This period represented a significant and robust binding event between the drug and the protein, where they established multiple contacts. In the kanzonol B-(GABA-T) complex, residue ASP239 showed a continuously deep orange band, which explained that this residue was in strong interaction with the ligand. The results were analogous to the histogram data (Fig. 12 (e)). The residue ASP239 formed more than 100 % hydrogen bonding interactions during the simulation time, indicating the possibility of the protein residue making multiple contacts of the same subtype with the ligand. The system was standardized, and no changes in density, volume, or kinetic energies were detected (Fig. 12 (a), (c), (e)).

RMSD provides insight into the structural stability and magnitude of ligand fluctuations during the simulation. As illustrated in Fig. 12 (i), the equilibrium RMSD was observed from 5 ns of simulation time up to 80 ns at around 0.8 Å. There was a sudden jump at 80 ns from 0.8 to 1.6 Å, indicating that there might be a conformational change in the molecule. Radius of gyration (rGyr) offers a measure of the ligand’s compactness, with a stable rGyr value indicating that the ligand maintains its structure throughout the simulation. The rGyr of the ligand showed an equilibrium up to 80 ns with 5.4 Å, and then suddenly the rGyr value dropped down to 5 Å, and its stability was retained until the end of 100 ns. Kanzonol B did not show any intramolecular hydrogen bonds. MolSA offers a glimpse into the ligand’s overall shape and size, with any changes indicating potential conformational alterations during the simulation. The MolSA was stable over most of the simulation time at around 316 Å^2^. SASA estimates the area of a ligand’s surface exposed to solvent, and the PSA measures the ligand’s ability to interact with polar solvent molecules. This was stable until 80 ns at around 134 Å^2^ (Fig. 12 (i)). The ligand properties showed some fluctuation at the end of the simulation, indicating small conformational changes in the ligand structure in the active site of the protein. Upon comparison with the reference, kanzonol B displayed better results with higher average PSA, and MolSA contributing to maintaining the stability of kanzonol B in the active site of the protein (Fig. 12 (i)). The SASA showed stable equilibrium until 60 ns, then there is a decrease in the SASA value, indicating that the kanzonol B might be buried in the protein, which shows the stability of the ligand–protein complex.

#### 4.5.2 PDFC32- human mitochondrial branched chain aminotransferase (BCATm) complex

For a drug to produce an agonistic or antagonistic effect on a protein, it should first remain stable within the protein. This requires the drug to stabilize specific conformational states of the receptor, particularly specific conformational states in the protein binding pocket, to achieve the desired biological response. Therefore, measuring the RMSD of a drug candidate and comparing it with reference drugs is important for assessing the stability of the drug within the protein. A lower RMSD indicates a better model.

A lower RMSD value for carbamazepine-BCATm complex (2.304 ± 0.69 Å) as compared to kanzonol B-BCATm complex (2.681 ± 0.37 Å), indicating carbamazepine-BCATm complex was more stable than the test compound-protein complex. While protein in the ligand–protein complex was unstable at the beginning of the simulation, which might be due to the accommodation process between the ligand and protein. Around 15.5 ns, there was a rise in RMSD to 3.063 Å, then it stabilized until 100 ns with an average RMSD of 2.77 Å (Fig. 13 (a)). Similarly, the RMSD values observed for the carbamazepine-BCATm complex reached a stable equilibrium around 40 ns. Kanzonol B RMSD was stable in the complex, and the average RMSD was 2.264 ± 0.702 Å. However, a sudden jump was observed at 62.8 ns from 1.8 to 2.6 Å, followed by stability until the end at 2.8 Å. This sudden jump suggested a possible conformational change in the molecule. The standard carbamazepine showed a high average RMSD with 2.268 ± 0.773 Å in the molecular dynamic’s trajectory (Fig. 13 (b)). The result indicates that BCATm has periodic backbone stability throughout the MD simulation while bound with the ligand.

Fig. 13 (c) flexibility of residues 36–57, 81–94, 109–129, 150–170, and 207–221 of BCATm is due to these domains not possessing ligands. The plot in Fig. 13 (c) showed that the amino acid residues of the protein behaved like standard drug-BCATm (Fig. 13 (d)) during simulation, with both plots showing comparable fluctuation patterns and trends. In comparison, the binding of kanzonol B had slightly higher fluctuations than the standard drug carbamazepine with BCATm. However, the presence of secondary structure elements (SSE), such as alpha-helices and beta-strands, in the protein made the structure more rigid. The higher SSE indicates that in the PDFC32-BCATm complex, the protein maintained a well-structured conformation during the simulation. Protein secondary structure elements (SSE) like alpha-helices and beta-strands are monitored throughout the simulation and shown in Fig. 13 (c) and (d).

Simulation interaction diagrams throughout the entire MD run provided insights into the interaction pattern between the ligands and BCATm protein. In the plot, the X-axis represents the residues interacting with the ligand, while the Y-axis displays the bond fraction. The 2D interaction diagram suggested that the complex was stabilized by hydrogen bonding with ARG99, ASN 242, LEU266, SER311 and THR313, pi-cationic interaction with ARG99, and hydrophobic interaction with TYR173, MET241 ALA314, and LEU266. Water bridges with LYS202, VAL270 and LEU266 were also observed. Among the conventional hydrogen bonds, SER311 demonstrated the highest interaction fraction, around 80 % with 2nd and 4th –OH groups of kanzonol B, followed by ARG99 at around 66 % with −C=O of ligand (Fig. 13 (g)). These hydrogen bond interactions were also observed in molecular docking studies.

A darker shade of orange in Fig. 13 (h) indicates the amino acid residues with more significant contacts. At the beginning of the simulation, there was a relatively lower number of contacts. However, as time progressed, the number of contacts increased, peaking between 40 to 100 ns, indicating a substantial level of interaction between kanzonol B and BCATm protein. This period represented a major binding event between the drug and the protein, where they formed multiple contacts. Our analysis revealed that the residues SER311 and ARG99 consistently demonstrated a deep orange band, suggesting that these residues were in strong interaction with the ligand during this period of simulation. The results were consistent with the histogram data (Fig. 13(e)). The residue SER311 formed around 80 % hydrogen bonding interactions during the simulation time, indicating the possibility of the protein residue making multiple contacts of the same subtype with the ligand. These interactions contributed to the stability of the protein–ligand complex throughout the entire duration of the MD simulation study. The system was standardized, and no changes in density, volume, or kinetic energies were detected (Fig. 13 (a), (c), (e)).

The sudden jump in RMSD suggested a possible conformational change in the molecule. The RMSD trajectories of kanzonol B demonstrated a minimum fluctuation throughout the simulation, indicating good stability. The rGyr measures the ligand’s compactness, and a stable rGyr value indicates that the ligand maintains its structure throughout the simulation. Initially, the rGyr value of kanzonol B showed an equilibrium up to 37 ns with 4.8 Å and then suddenly the rGyr value increased to 5.07 Å. Its stability was then retained until the end of 100 ns (Fig. 13 (i)). Kanzonol B did not form any intramolecular hydrogen bonds. MolSA enables the understanding of the ligand’s overall shape and size, with any changes indicating potential conformational alterations during the simulation. Initially, the SASA values of the ligands fluctuated slightly before reaching equilibrium. This may be due to the ligand initially reorienting itself within the active site. Eventually, the ligand settled and attained a stable conformation, with an SASA value of 130.55 Å^2^ at the equilibrium (Fig. 13 (i)). During the simulation, the ligand’s ability to interact with polar solvent molecules was measured by its PSA. The ligands’ PSA values ranged between 135.07 and 146.87 Å^2^, which means that the properties of the ligand initially changed slightly but attained equilibrium and remained constant throughout the simulation. This demonstrates that the ligand was stable in the protein’s active region. The equilibrium value of 139.77 Å^2^ shows that there was a balanced distribution of polar contacts, which contributed to the stability of the protein–ligand complex. Compared to the reference standard, kanzonol B showed better results, with a higher average PSA of 139.77 Å^2^ and a MolSA of 314.85 Å^2^, which helped maintain its stability in the protein's active site. However, the average SASA of kanzonol B was higher than that of the reference ligand, at 130.55 Å2 (Fig. 13 (i)). This higher SASA could be due to weak ligand binding or structural rearrangements that contribute to the low stability of the protein–ligand complex.

#### 4.5.3 PDFC32- human voltage-gated sodium channel, brain isoform (Nav1.2) complex

A lower RMSD value of kanzonol B-HVGSC complex (3.445 ± 0.46 Å) (Fig. 14 (a)) as compared to the carbamazepine-HVGSC complex (6.344 ± 0.88 Å) (Fig. 14 (b)), indicating the kanzonol B-HVGSC complex is more stable than the standard drug-protein complex. Although an RMSD value below 3 Å is generally considered the threshold value for RMSD, even though the RMSD values higher than 3 Å have been reported in other studies.^137^, ^138^ Notably, at the 8.2 ns timestep, the RMSD trajectory of kanzonol B-HVGSC complex had demonstrated a fluctuation from 2.754 to 3.658 Å, which is likely due to the ligand adjusting within the binding pocket of the protein. Then the RMSD trajectory was depicted steadily till the end of the MD simulation with an average RMSD of 3.551 Å (Fig. 14 (a)). In contrast, the carbamazepine-HVGSC complex showed a sudden rise at around 6.7 ns from 6.492 to 7.225, followed by a drop to around 5 Å at around 16.7 ns. It reached a stable equilibrium around 20 ns and remained steady throughout the rest of the simulation (Fig. 14 (b)). For the kanzonol B, the RMSD was stable in the complex, with an average RMSD of 4.269 ± 1.04 Å. However, a sudden jump was observed at 13.5 ns from 2.9 to 4.5 Å, followed by stability until the end of the simulation. This sudden jump suggested a possible conformational change in the molecule. On the other hand, the standard carbamazepine showed a higher average RMSD with 4.397 ± 0.82 Å in the molecular dynamics’ trajectory. The result indicates that the HVGSC has periodic backbone stability throughout the MD simulation, while bound with the ligand, and kanzonol B has the potential to act as an antiepileptic agent. In comparison, among the RMSD trajectory plots of the protein–ligand complexes obtained, the RMSD trajectory of the human voltage-gated sodium channel complex showed the highest stability with steady peaks.

A small fluctuation on residues 5–7, 23–33, 54–60, and 66–77 of the HVGSC protein, as shown in Fig. 14 (c) and (d), is due to these domains not possessing ligands. The plot in Fig. 14 (c) represents that the amino acid residues of the protein behaved like the carbamazepine-HVGSC (Fig. 14 (d)) during simulation, with both plots showing comparable fluctuation patterns and trends. Interestingly, the binding of kanzonol B had slightly reduced the fluctuations than the standard drug carbamazepine with human voltage-gated sodium channel. The exhibition of the least fluctuations confirmed the strong attachment of the ligand to the protein. The RMSF data for kanzonol B-HVGSC complex suggests that the presence of kanzonol B does not lead to significant increases in structural flexibility, which is often associated with destabilization. Rather, the ligand seems to maintain or even enhance the structural integrity of the proteins. The secondary structure elements (SSE), such as alpha-helices and beta-strands in the protein, contribute to the rigidity of the structure. The higher SSE indicates that in the kanzonol B-HVGSC complex, the protein maintained a well-structured conformation during the simulation. Protein secondary structure elements (SSE) are monitored throughout the simulation.

In the plots, the X-axis represents the residues involved in ligand interactions, while the Y-axis shows the bond fraction. The 2D interaction diagram suggested that the complex was stabilized by hydrogen bonding with GLU1788, GLU1792, ALA1874; hydrophobic interaction with PHE1795, PHE1859, LEU1875, LEU 1858 and LEU1866; and water bridges with GLU1871, GLN1878, and ALA1874. Among the conventional hydrogen bonds, GLU1788 demonstrated the highest interaction fraction, around 60 % with 4th –OH groups of kanzonol B (Fig. 14 (g)). In our molecular docking studies, the GLU1788 residue was found to be involved in van der Waals interactions, and the residues GLU1868, SER1869 and VAL1865 were involved in hydrogen bonding, indicating that all the amino acid residues involved in the interaction are in and around the binding pocket.

In Fig. 14 (h), a darker shade of orange represents residues with more significant contacts. At the beginning of the simulation, kanzonol B and HVGSC were initially distant from each other, leading to a relatively lower number of contacts. However, as time progressed, the number of contacts increased, peaking between 20 to 100 ns, indicating a substantial level of interaction between kanzonol B and HVGSC protein, where they formed multiple contacts. Our analysis revealed that in the kanzonol B-HVGSC complex, there are multiple red and deep orange bands with residue GLU1788, which explains that this specific residue is in strong interaction with the ligand in all probable alignments. The results were consistent with the histogram data (Fig. 14 (e)). The residue GLU1788 formed around 60 % hydrogen bonding interactions during the simulation time, which contributed to the stability of the protein–ligand complex throughout the entire duration of the MD simulation study. The system was standardized, and no changes in density, volume, or kinetic energies were detected (Fig. 14 (a), (c), (e)).

The plateau RMSD of 1.703 Å was reached at 8.7 ns, and its stability was retained until the end of the 100-ns simulation. The sudden increase in the RMSD suggested a possible conformational change in the molecule. Throughout the simulation, the RMSD trajectories of kanzonol demonstrated a minimum fluctuation, indicating good stability. Initially, the rGyr value of kanzonol B dropped at 10.6 ns but then stabilized up to 100 ns with an average value of 5.12 Å. Kanzonol B did not form any intramolecular hydrogen bonds (Fig. 14 (i)). The MolSA was stable over most of the simulation time at around 316.80 Å^2^. The ligand settled and attained a stable conformation, with an SASA value of 141.43 Å^2^ at the equilibrium. The equilibrium value of 136.25 Å^2^ shows that there was a balanced distribution of polar contacts, which contributed to the stability of the protein–ligand complex (Fig. 14 (i)). Upon comparison with the reference standard ligand compound, the kanzonol B displayed better results with a high PSA of 136.25 Å^2^, and MolSA of 316.80 Å^2^, contributing to maintain the stability throughout the simulation. However, the average SASA of PDFC32 was higher than that of the reference ligand, at 141.43 Å^2^ (Fig. 14 (i)). This higher SASA could be due to ligand binding or structural rearrangements that contribute to the stabilization of the protein–ligand complex.

#### 4.5.4 PDFC32- Leucine-rich glioma inactivated 1 (LGI1) complex

A lower RMSD value of the carbamazepine-LGI1 complex (1.238 ± 0.19 Å) as compared to kanzonol B-LGI1 complex (1.454 ± 0.21 Å) indicates that the carbamazepine-LGI1 complex was more stable than the kanzonol B-LGI1 complex. During the simulation, the protein was stable throughout the simulation in the kanzonol B-protein complex, maintaining a plateau in RMSD from 10.5 ns to 55 ns with an average RMSD of 1.380 Å. There was a significant drop to 1.17 Å at 56.8 ns, after which the RMSD achieved equilibrium until the end of the simulation (Fig. 15 (a)). Similarly, the RMSD values observed for the carbamazepine-LGI1 complex reached a stable equilibrium around 17 ns. Kanzonol B RMSD showed instability at the beginning of the simulation with a rise to 61 Å around 7.7 ns. The RMSD value observed was significantly larger, indicating the possibility that the ligand had a different binding mode away from its initial binding site or major rearrangement of the molecule. However, it was then stabilized until 42 ns. A significant drop was observed around 53 ns, reaching 23.701 Å, and then it maintained stability until the end of the simulation, with an average RMSD of 22.721 Å. These fluctuations in the RMSD values suggested a possible conformational change in the molecule. When comparing the RMSD values of the individual ligand, the standard carbamazepine showed a lower average RMSD of 21.433 ± 11.48 Å in the molecular dynamics trajectory, whereas the kanzonol B had a higher average RMSD of 33.340 ± 15.49 Å. Overall, the result indicates that LGI1 has periodic backbone stability throughout the MD simulation while bound with the ligand.

The amino acid residues 25–33, 69–78, 86–100, 117–130, and 143–158 of LGI1 showed notable fluctuations, which may have been brought by the increased flexibility (Fig. 15 (c)). The RMSF plot in Fig. 15 (c) indicated that the behavior of amino acid residues of protein was like standard drug-LGI1 (Fig. 15 (d)) during simulation, both plots demonstrating comparable fluctuation patterns and trends. However, when comparing the two complexes, the binding of kanzonol B had slightly reduced fluctuations than the standard drug carbamazepine with LGI1. In the presence of secondary structure elements (SSE), such as alpha-helices and beta-strands in the protein, the protein structure is more rigid. The higher SSE in the PDFC32- Leucine-rich glioma inactivated 1 (LGI1) complex indicates that the protein was more rigid in conformation with fewer fluctuations. The distributions of these SSE are monitored throughout the simulation and shown in Fig. 15 (c) and (d).

The hydrogen bonding with ASP218, GLU56, ILE222, and hydrophobic interaction with TYR206, LEU214, LEU54 and VAL75 of LGI1 by kanzonol B indicates the stability of the complex. Besides, water bridges were also observed with ALA44, GLU56, and GLU205. The residues ARG76 and ARG209 also formed pi–pi stacking interactions with the ligand. Among the conventional hydrogen bonds, ASP218 demonstrated the highest interaction fraction, around 20 % with 4th-OH groups of kanzonol B (Fig. 15 (g). However, in molecular docking studies, ASP218 was not involved in hydrogen bonding, while ILE61, SER83, GLU81 and SER83 were found to form hydrogen bonds. In the molecular dynamic study, GLU81 residue was found to form hydrogen bonds, but SER83 residue formed water bridges instead (Fig. 15 (g). The residues GLU81, ILE61, ILE82, PHE79, SER60, SER83 and SER86 have been previously reported to be involved in the interaction between ethosuximide and LGI1 complex, which contributes to the treatment of absence seizures.^9^ These findings are consistent with our results, further supporting that kanzonol B may exhibit good antiepileptic potential by interacting with LGI1.

At the beginning of the simulation, there was a smaller number of contacts, but as the simulation progressed, the number of contacts increased, reaching a peak between 20 to 100 ns, suggesting a strong interaction between kanzonol B and LGI1 protein, where they formed multiple contacts. However, notably, the number of contacts dropped to 0 between 0 to 20 ns, suggesting the possibility that the ligand may have adopted a different binding mode away from its initial binding site or major rearrangement of the molecule, which is consistent with the result shown in Fig. 15 (a). Our analysis revealed that in the kanzonol B-LGI1 complex, there are multiple red and deep orange bands with residue ASP218, which explains that this specific residue is in strong interaction with the ligand in all probable alignments. The results were aligned with the histogram data (Fig. 15 (e)). The residue ASP218 formed around 20 % hydrogen bonding interactions during the simulation, contributing to the stability of the protein–ligand complex throughout the MD simulation. The system was standardized, and no changes in density, volume, or kinetic energies were detected (Fig. 15 (a), (c), (e)).

These fluctuations in RMSD (Fig. 15 (i)) suggested that there might be a possible conformational change in the molecule. Kanzonol B showed an average rGyr value of 5.10 Å during the 100 ns MD simulation. The kanzonol B had demonstrated heavy fluctuation in rGyr throughout the simulation, indicating potential conformational changes during the simulation. Intramolecular hydrogen bonding was also observed within the ligand. These intramolecular hydrogen bonds contribute to the structural rigidity of the ligand. However, this rigidity may limit the ligand's ability to adapt to minor conformational changes in the binding site of the target, potentially reducing binding affinity. The MolSA was stable over most of the simulation time at around 316.34 Å^2^. SASA estimates the area of a ligand’s surface exposed to solvent. The SASA of the ligand showed a significant fluctuation of up to 60 ns of simulation and then gradually reached average equilibrium at 326.28 Å^2^ (Fig. 15 (i)). The PSA of the ligand remained stable over the simulation with an average PSA of 137.46 Å^2^. Upon comparison with the reference standard ligand compound, the kanzonol B displayed better results with a higher average PSA of 137.46 Å^2^, and MolSA of 316.34 Å^2^, contributing to maintaining the stability of the ligand in the active site of the protein. However, the average SASA of kanzonol B was higher than that of the reference ligand, at 413.34 Å^2^ (Fig. 15 (i)). This higher SASA could be due to the ligand moving away from the protein or structural rearrangements that contribute to the low stability of the protein–ligand complex.

The current research work is mainly concerned with the in-silico prediction of antiepileptic activity of phytoconstituents of *C. odontophyllum* fruits and in-silico validation only. Furthermore, the in vitro and in vivo studies of the antiepileptic activity of the identified compound are a must to validate the current prediction in this research work.

## 5 Conclusion

In the present study, we performed molecular docking studies to explore the affinity of phytoconstituents of *C. odontophyllum* fruits against the proteins responsible for epilepsy. Our molecular docking and dynamic study results indicated that a phytochemical compound, kanzonol B (PDFC32) of *C. odontophyllum* fruits, showed a strong binding affinity for the active sites of proteins related to epilepsy. The obtained results indicated that it would be an effective multi-targeted antiepileptic. It adheres to Lipinski’s rule, has good bioavailability, and possesses excellent GI absorption, CNS permeability, and BBB permeability. Most importantly, it is considered safe for use with the toxicity class of V. All these parameters make kanzonol B a potentially promising agent for epilepsy treatment. Promising results were also obtained via molecular dynamics (MD) simulation analysis performed for four selected proteins (GABA-T, BCATm, Nav1.2 and LGI1) with kanzonol B. The RMSD and RMSF data confirmed the stability of proteins and ligands. The interaction between kanzonol B and the four selected proteins demonstrated that kanzonol B had good stability with the Human Voltage-gated Sodium Channel, brain isoform than other proteins. These computational studies suggested that kanzonol B may serve as a potent multi-targeted antiepileptic drug. However, further experimental studies and validations are important to confirm its potential for clinical use.

## CRediT authorship contribution statement

**Lim Joe Siang:** Writing – original draft, Validation, Software, Investigation, Formal analysis, Data curation. **Kamini** **Vijeepallam:** Writing – review & editing, Methodology, Funding acquisition, Formal analysis, Data curation, Conceptualization. **Arunachalam Muthuraman:** Writing – review & editing, Methodology, Investigation, Funding acquisition, Formal analysis, Data curation, Conceptualization. **Parasuraman Pavadai:** Writing – review & editing, Validation, Software, Resources, Methodology, Formal analysis, Conceptualization. **Thiruventhan Karunakaran:** Writing – review & editing, Visualization, Validation, Methodology, Investigation, Formal analysis, Conceptualization. **Veerasamy Ravichandran:** Supervision, Resources, Project administration, Methodology, Funding acquisition, Conceptualization.

## Declaration of competing interest

The authors declare that they have no known competing financial interests or personal relationships that could have appeared to influence the work reported in this paper.
