# Supplementary figures and images for: Exploration of Canarium odontophyllum fruit phytoconstituents as potential candidates against epilepsy using in silico studies
Source: J Genet Eng Biotechnol. 2025 Aug 26;23(4):100561. doi: 10.1016/j.jgeb.2025.100561 (PMC12409388; doi:10.1016/j.jgeb.2025.100561)

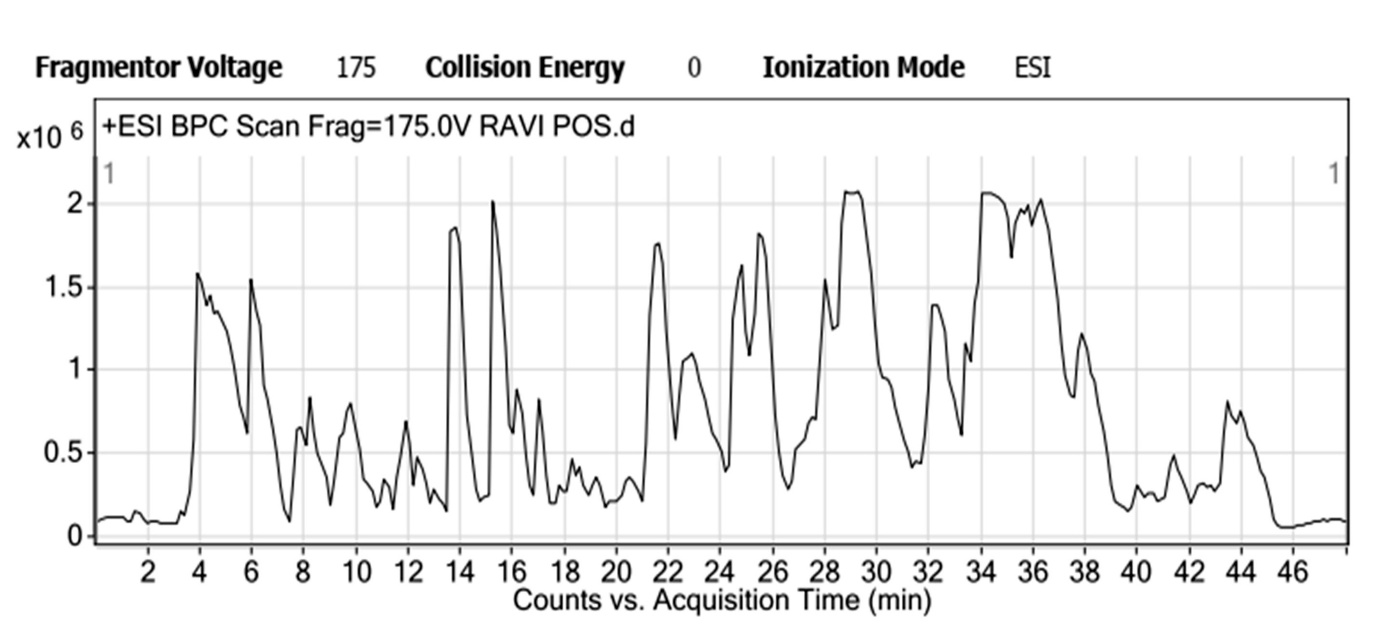

Supplement: Supplementary Data 1 [file mmc1.docx]

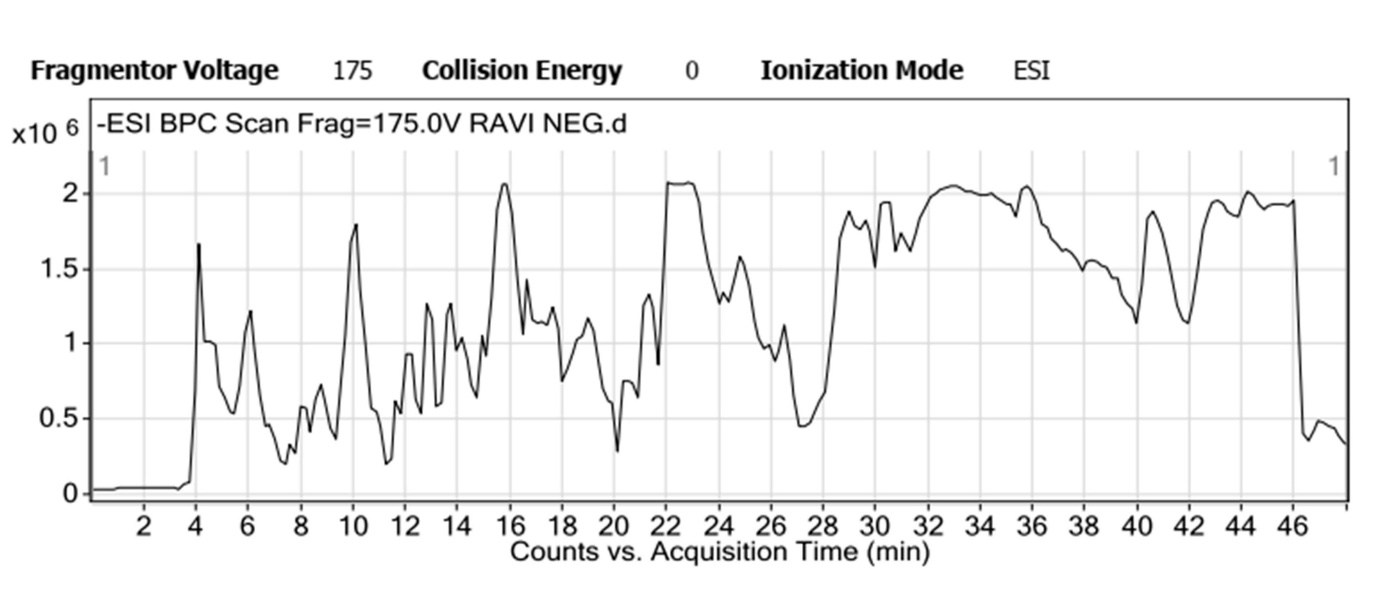

Supplement: Supplementary Data 2 [file mmc2.docx]

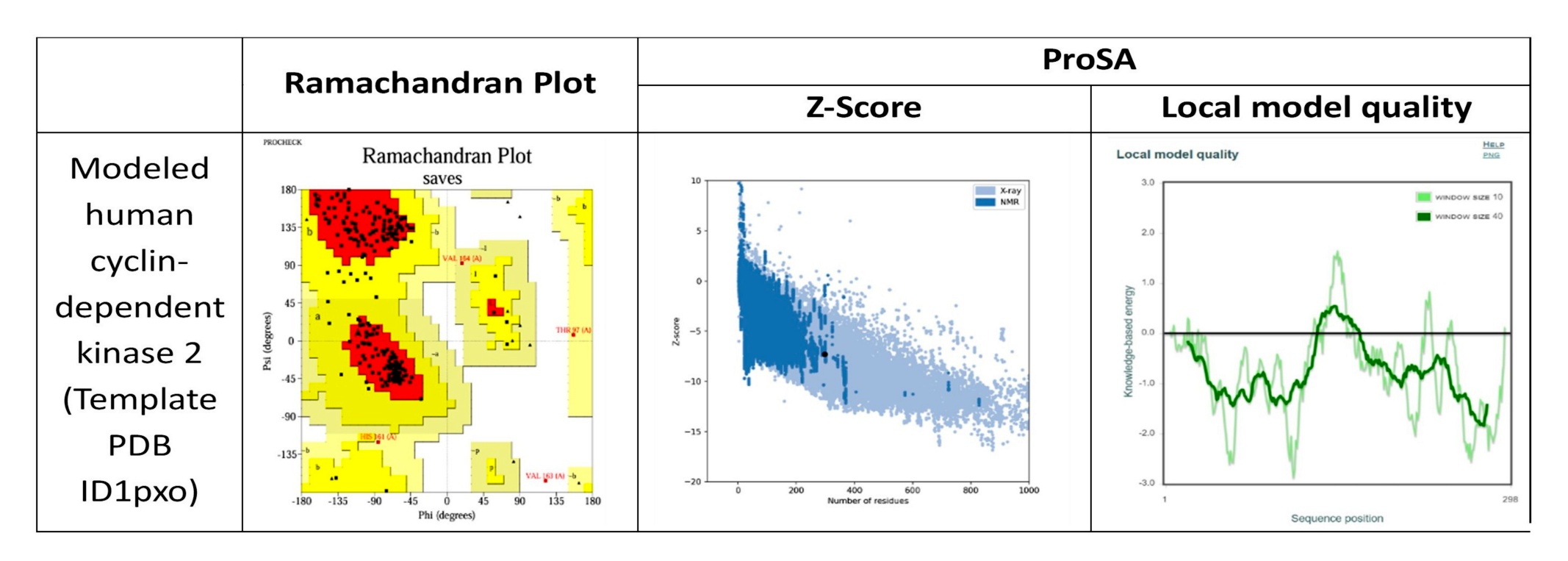

Supplement: Supplementary Data 3 [file mmc3.docx]
