# Supplementary material for: Exploration of Canarium odontophyllum fruit phytoconstituents as potential candidates against epilepsy using in silico studies
Source: J Genet Eng Biotechnol. 2025 Aug 26;23(4):100561. doi: 10.1016/j.jgeb.2025.100561 (PMC12409388; doi:10.1016/j.jgeb.2025.100561)
Supplement: Supplementary Data 4 [file mmc4.docx]

| NDFC1 | NDFC2 |
| --- | --- |
| 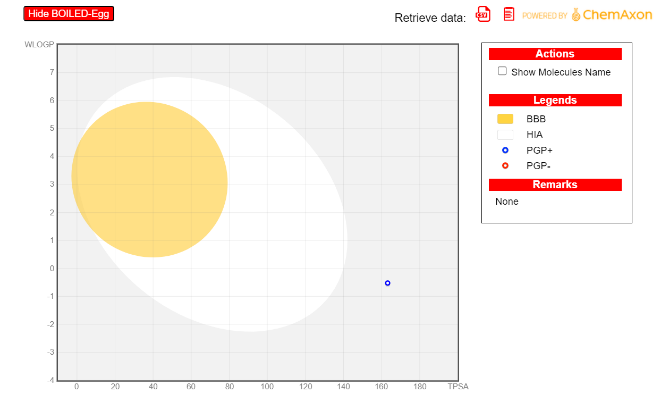 | 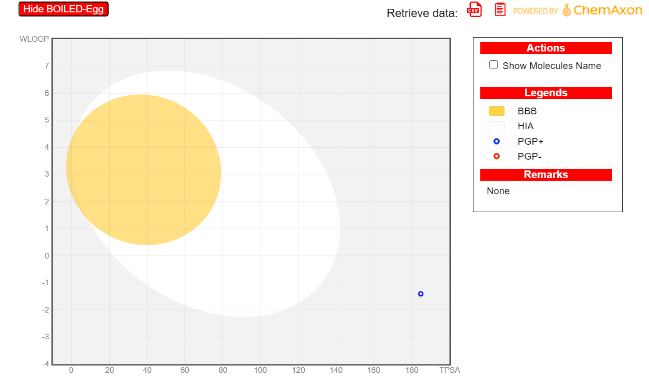 |
| NDFC3 | NDFC4 |
| 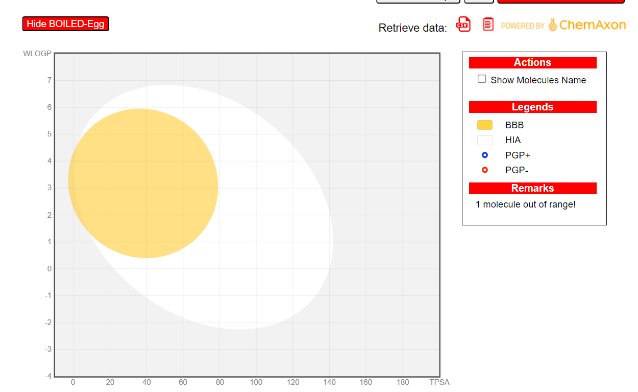 | 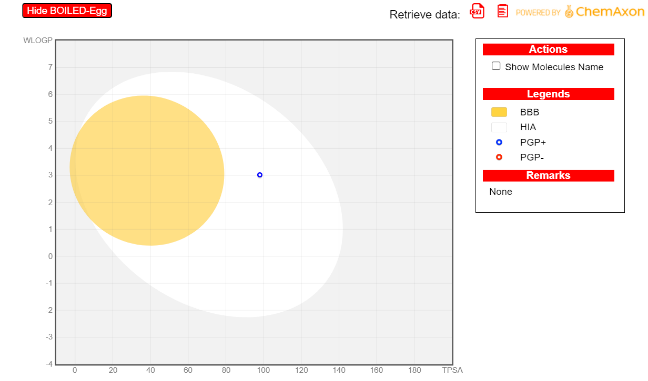 |
| NDFC5 | NDFC6 |
| 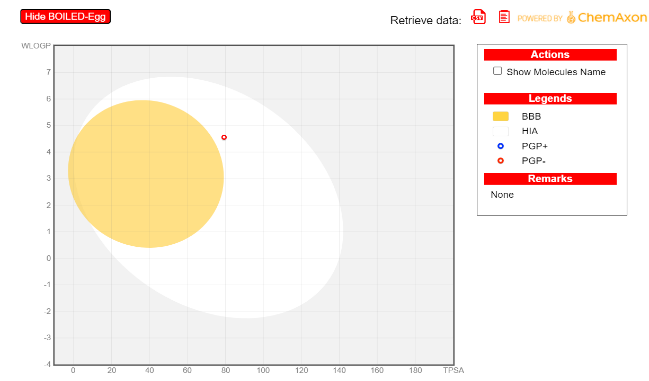 | 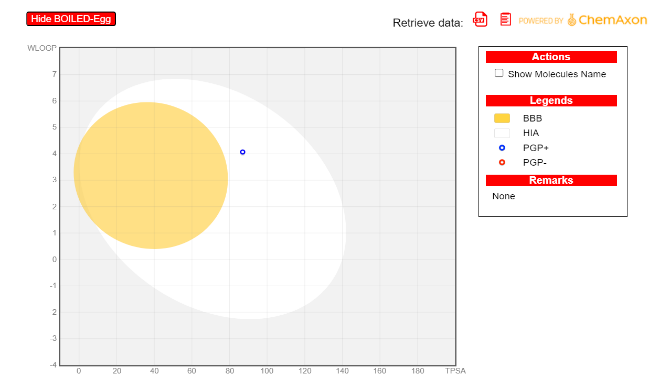 |
| NDFC7 | NDFC8 |
| 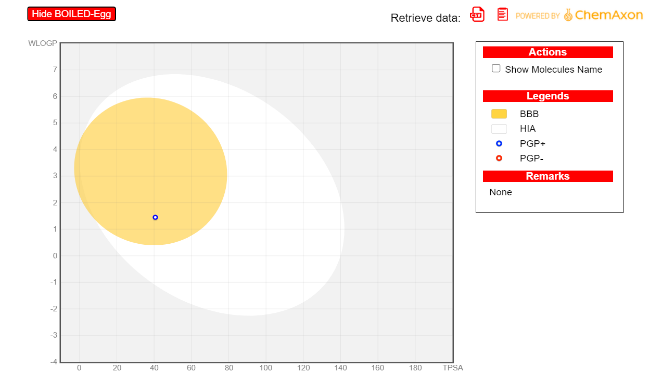 | 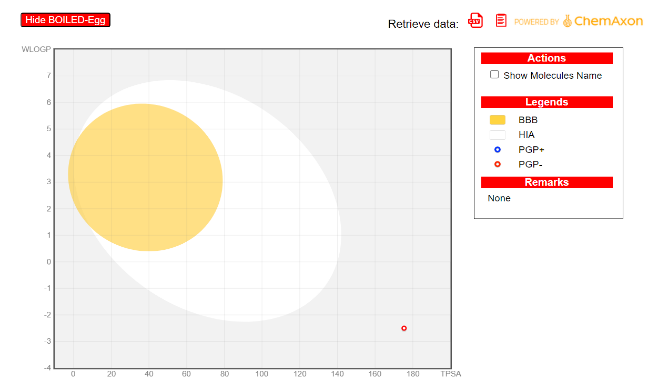 |
| NDFC9 | NDFC10 |
| 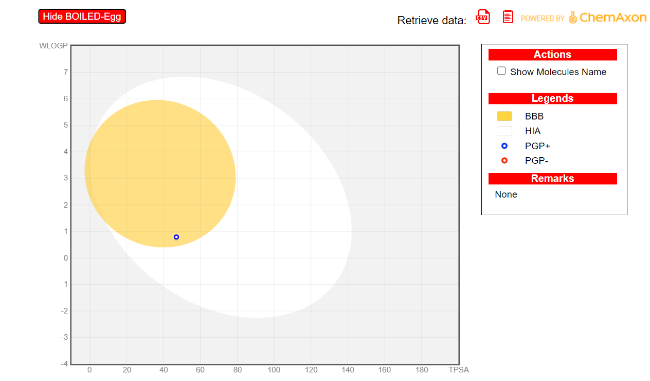 | 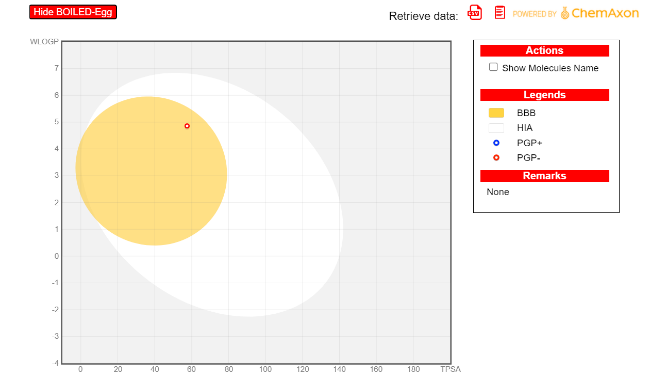 |
| NDFC11 | NDFC12 |
| 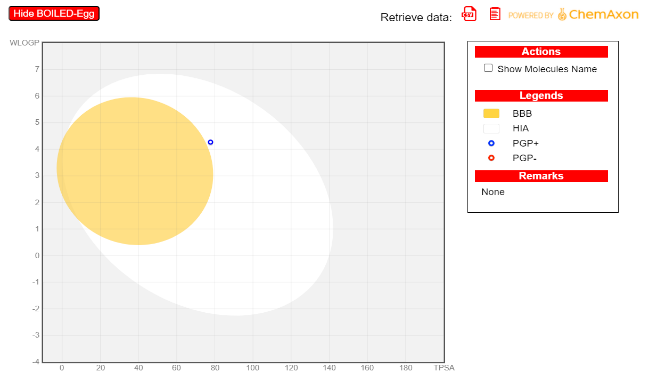 | 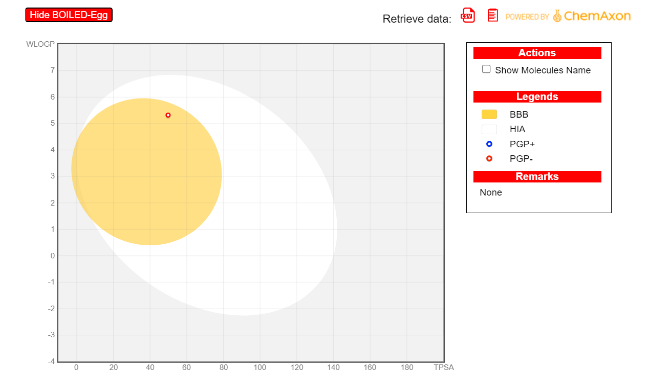 |
| NDFC13 | NDFC14 |
| 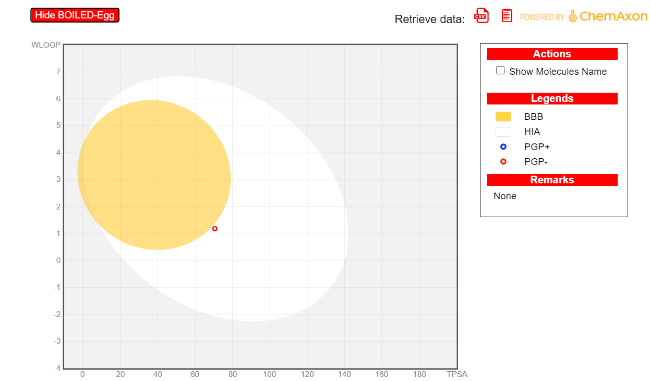 | 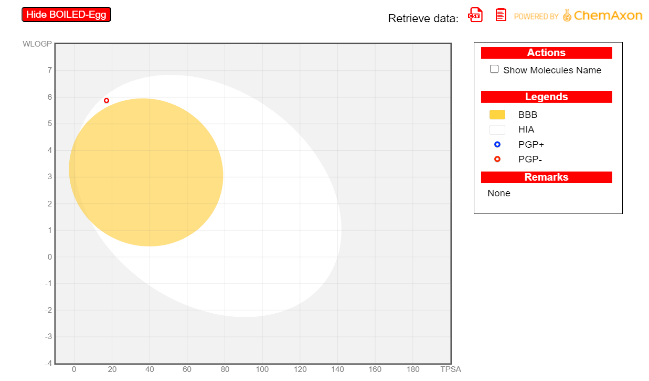 |
| PDFC1 | PDFC2 |
| 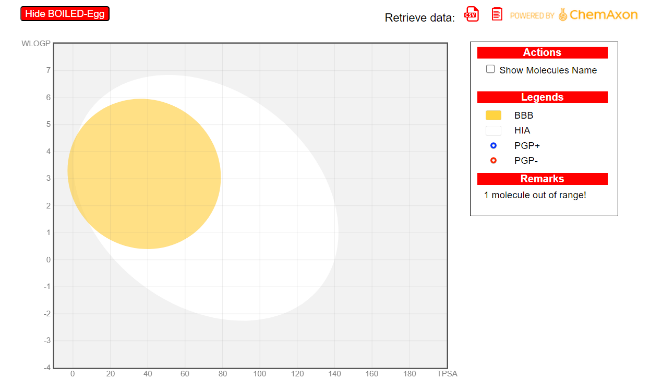 | 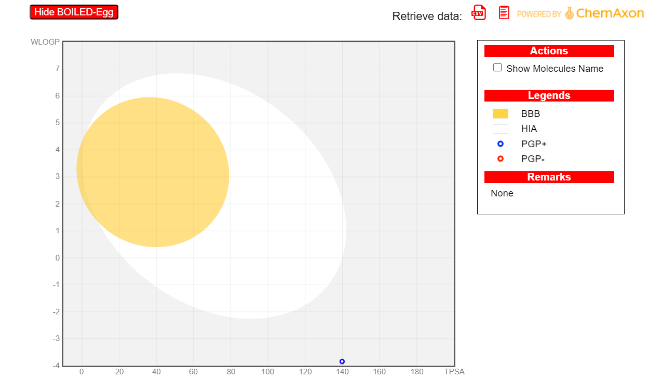 |
| PDFC3 | PDFC4 |
| 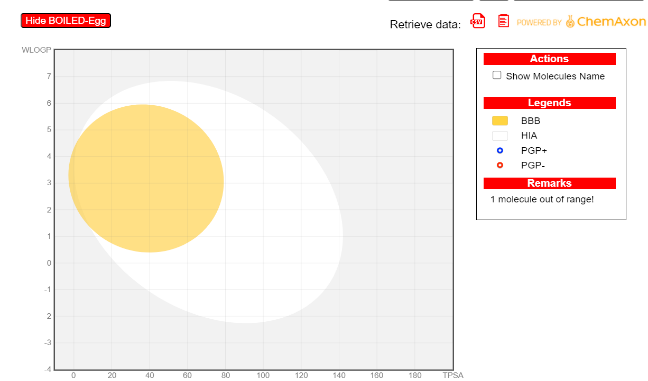 | 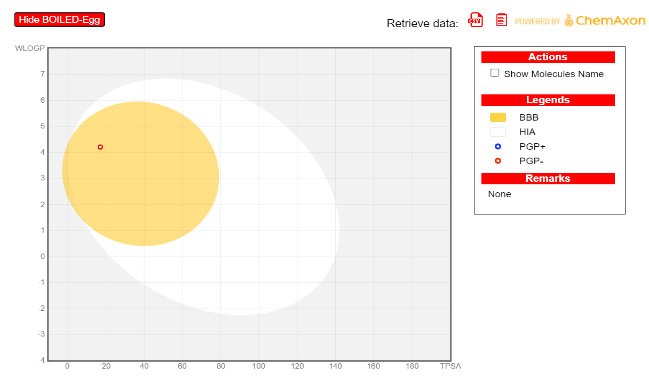 |
| PDFC5 | PDFC6 |
| 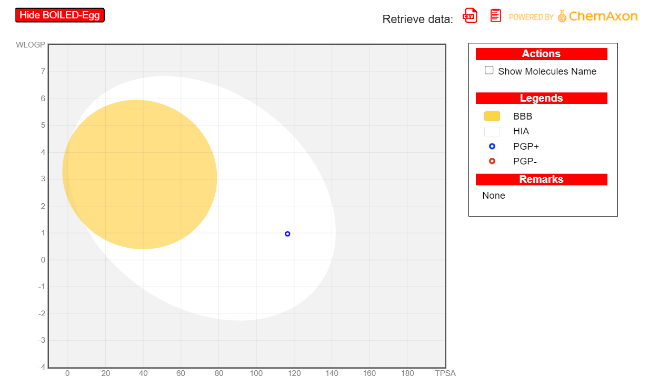 | 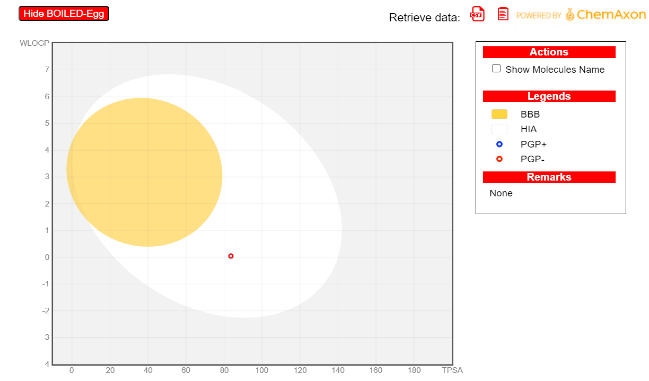 |
| PDFC7 | PDFC8 |
| 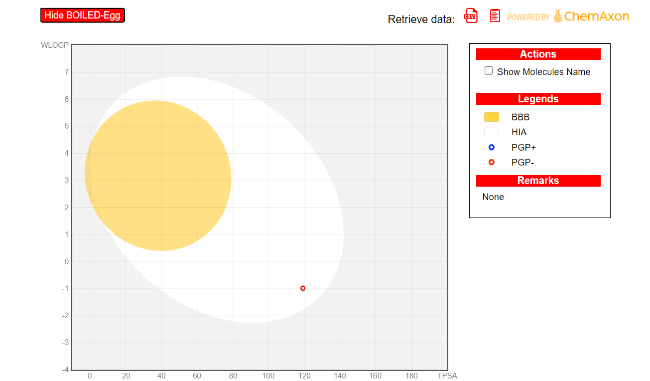 | 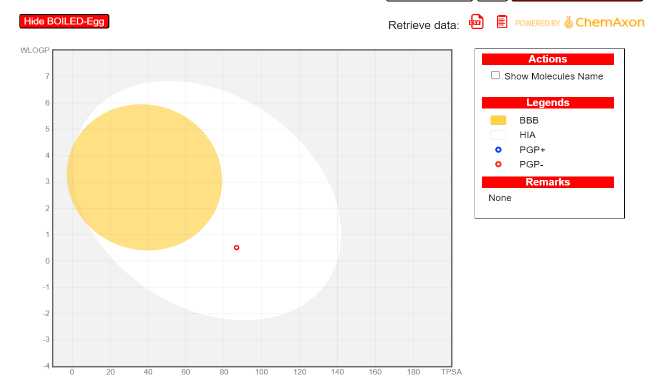 |
| PDFC9 | PDFC10 |
| 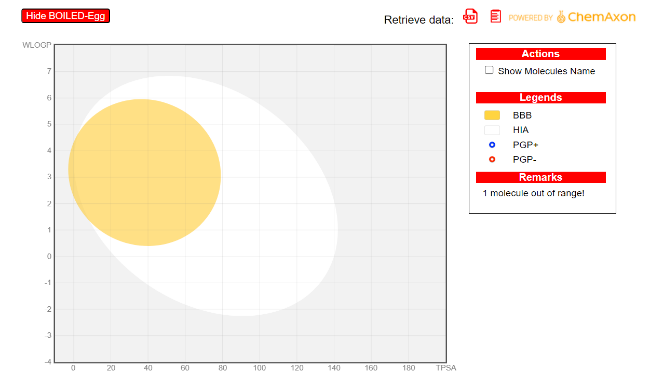 | 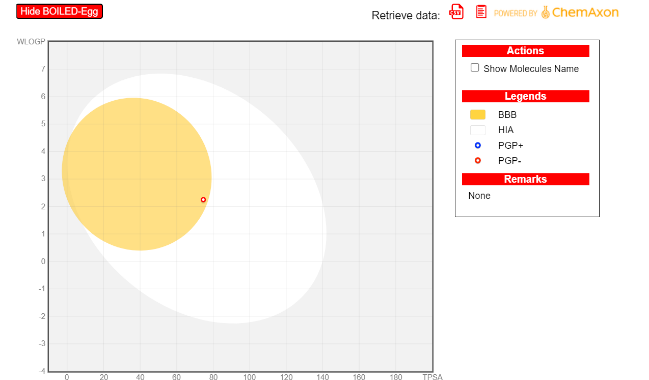 |
| PDFC11 | PDFC12 |
| 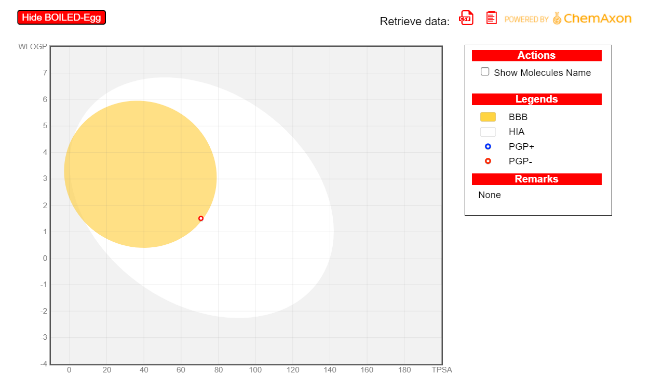 | 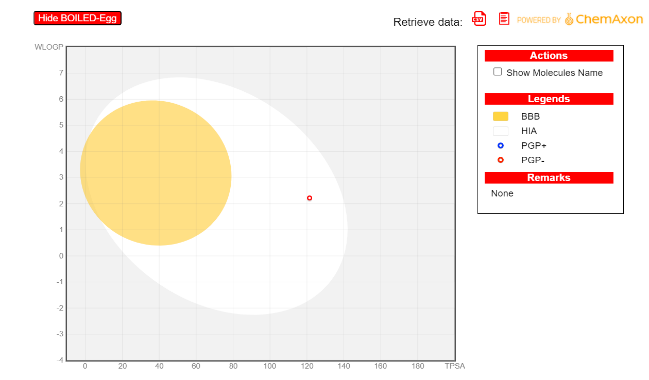 |
| PDFC13 | PDFC14 |
| 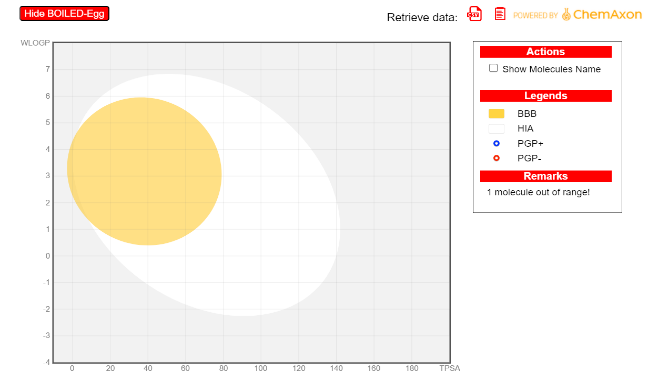 | 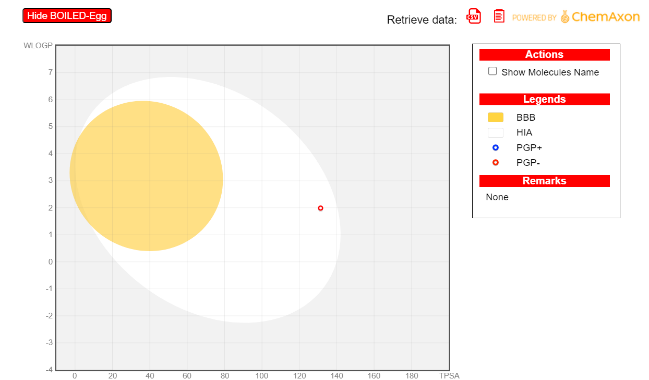 |
| PDFC15 | PDFC16 |
| 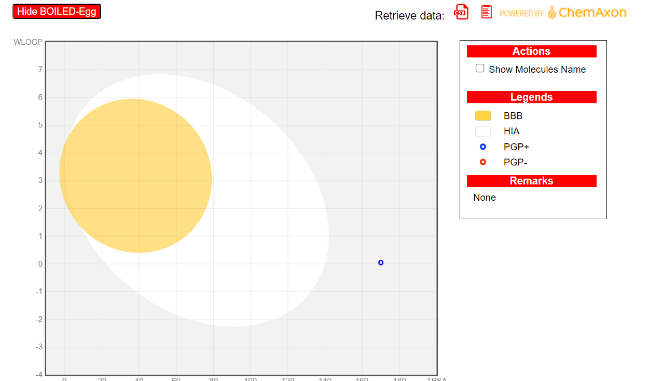 | 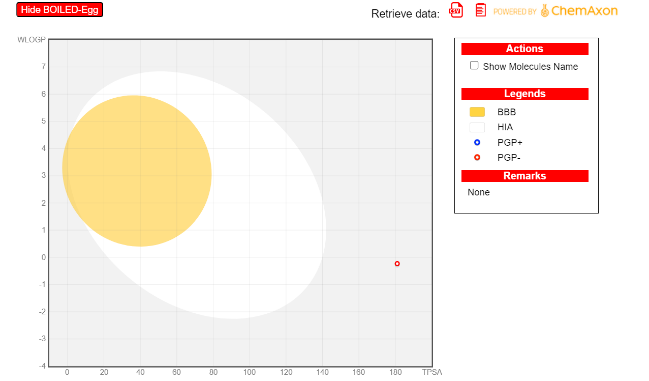 |
| PDFC17 | PDFC18 |
| 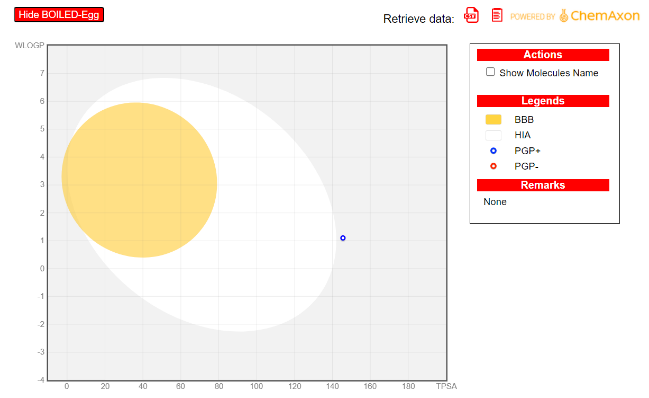 | 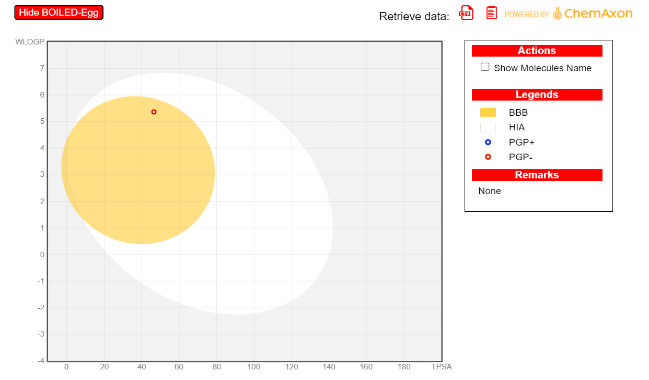 |
| PDFC19 | PDFC20 |
| 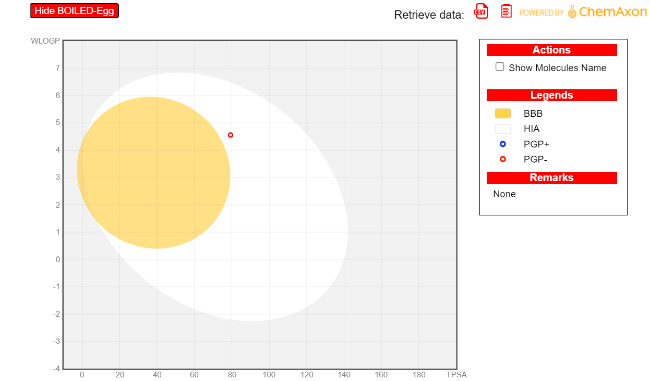 | 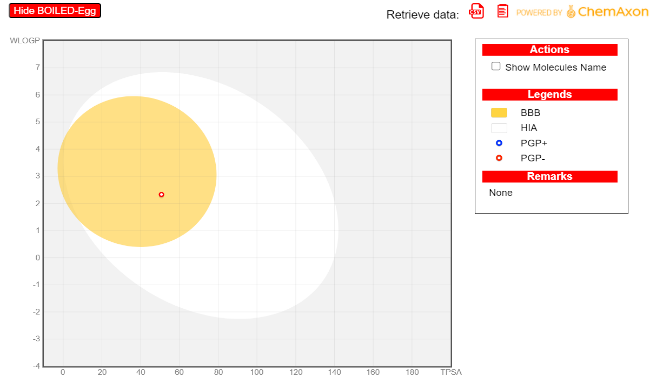 |
| PDFC21 | PDFC22 |
| 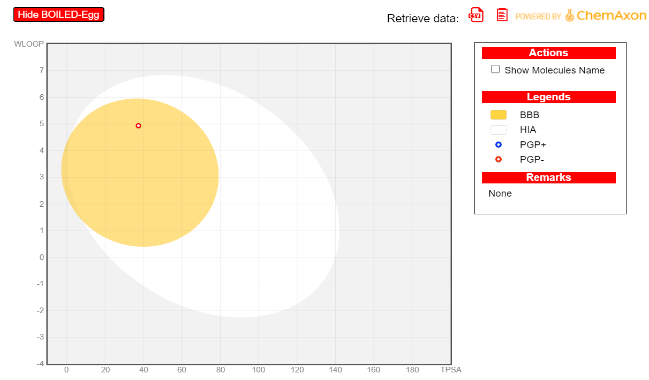 | 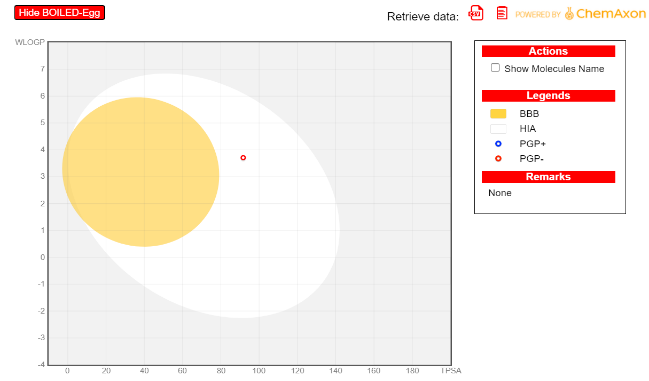 |
| PDFC23 | PDFC24 |
| 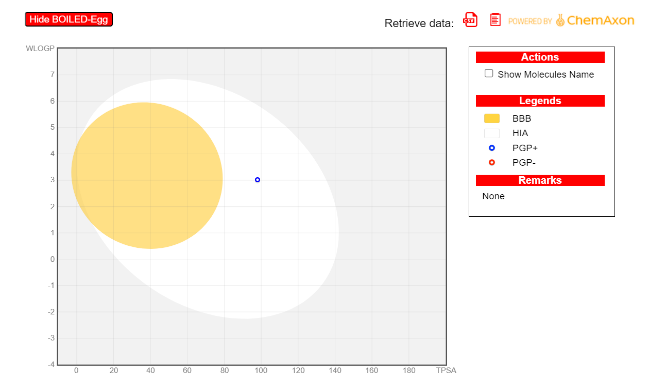 | 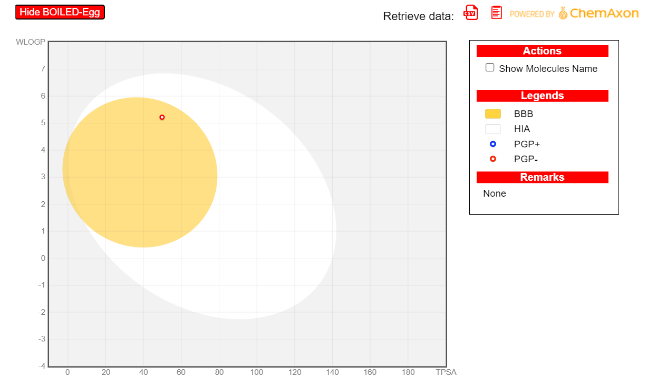 |
| PDFC25 | PDFC26 |
| 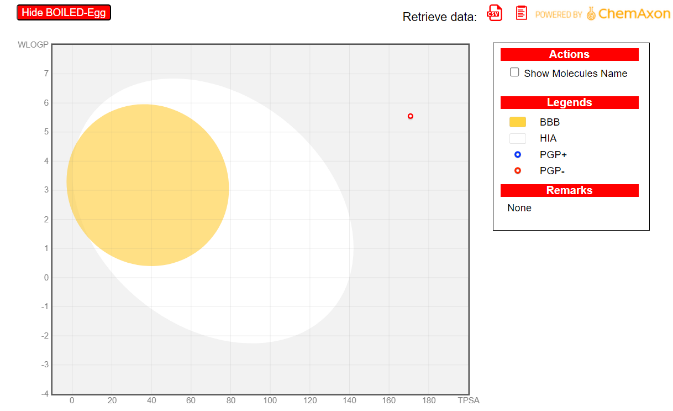 | 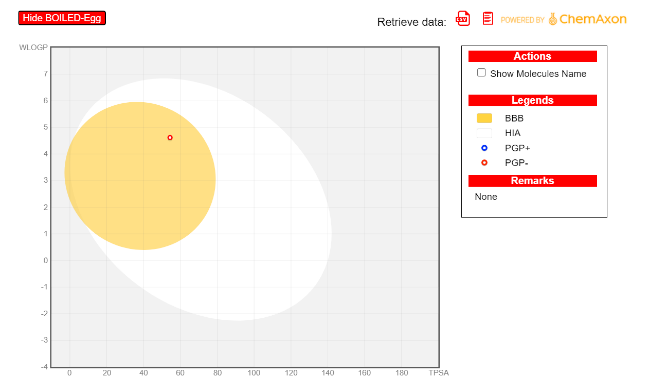 |
| PDFC27 | PDFC28 |
| 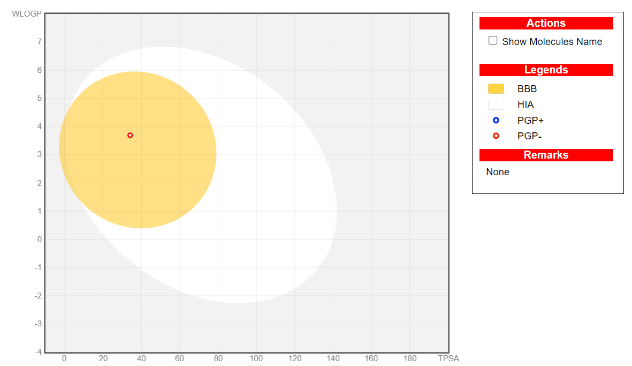 | 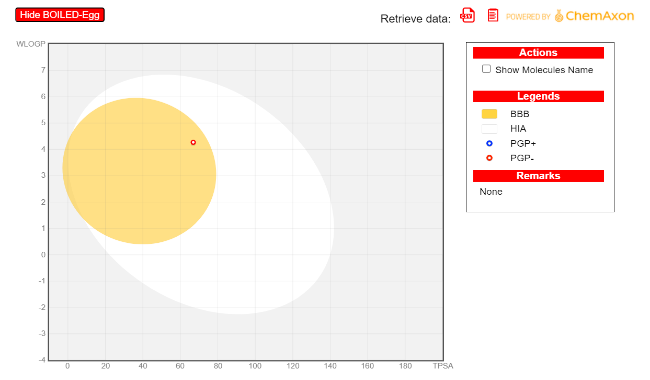 |
| PDFC29 | PDFC30 |
| 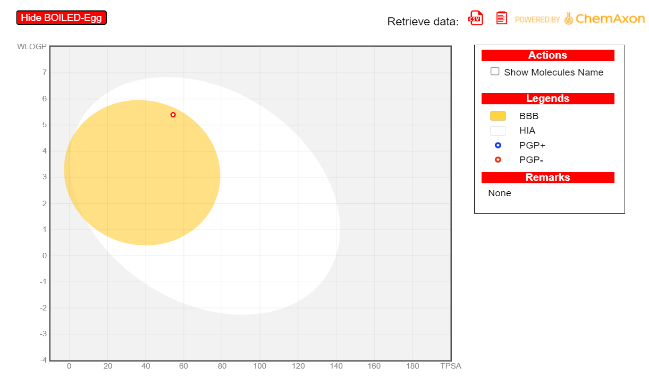 | 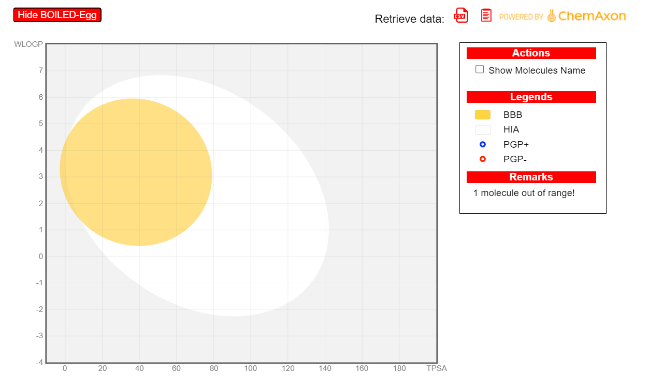 |
| PDFC31 | PDFC32 |
| 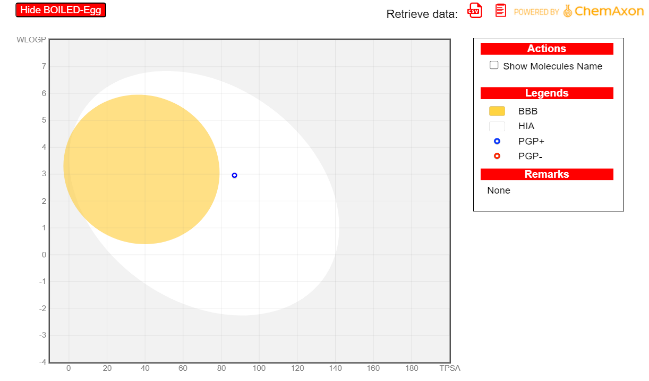 | 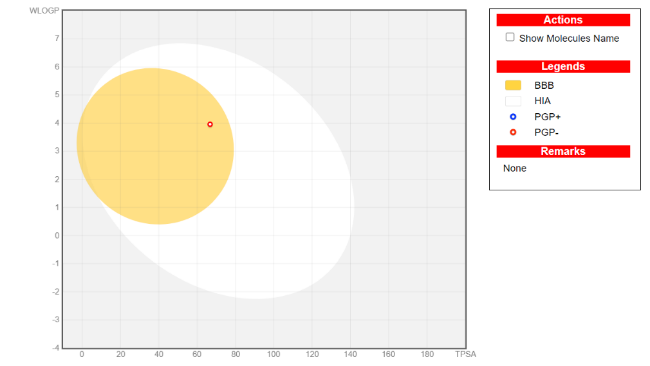 |
| PDFC33 | PDFC34 |
| 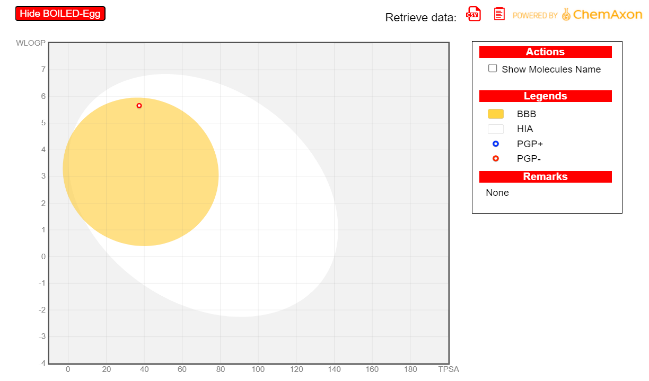 | 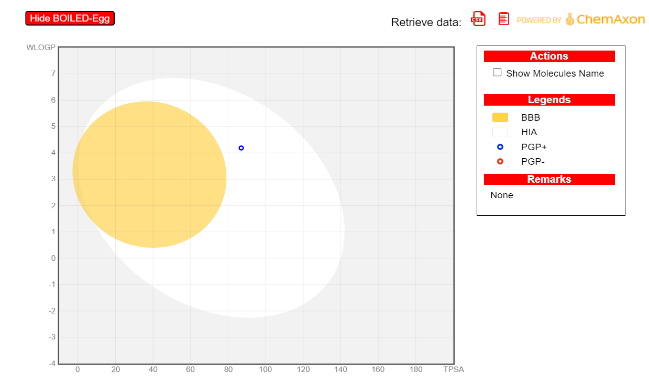 |
| PDFC35 | PDFC36 |
| 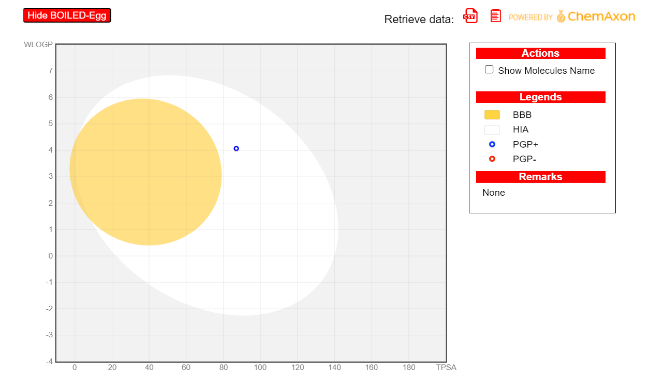 | 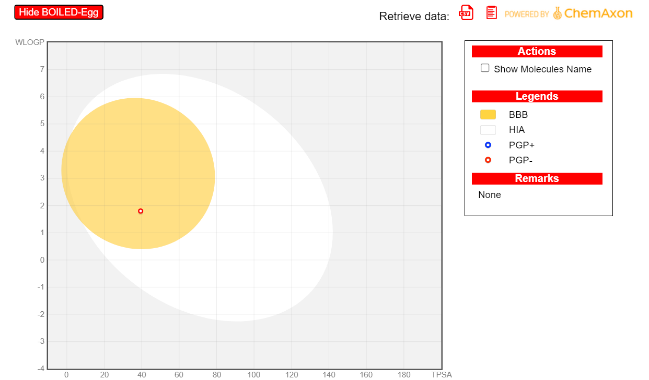 |
| PDFC37 | PDFC38 |
| 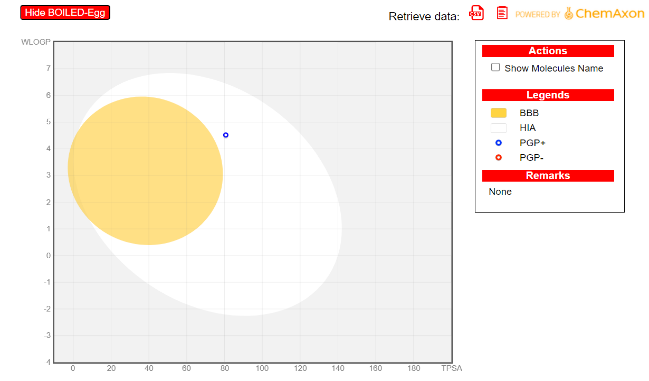 | 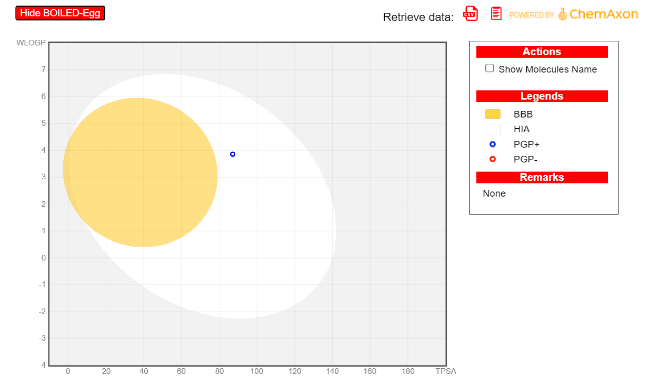 |
| PDFC39 | PDFC40 |
| 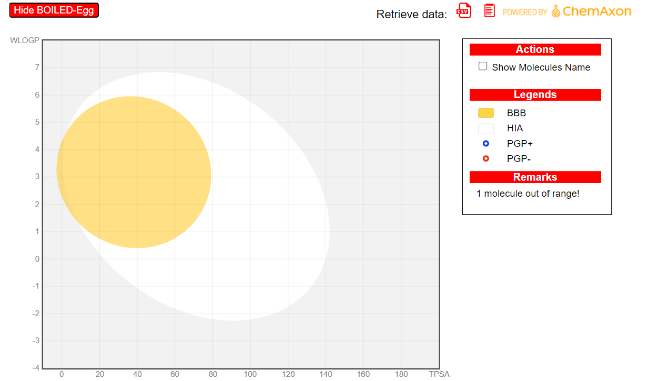 | 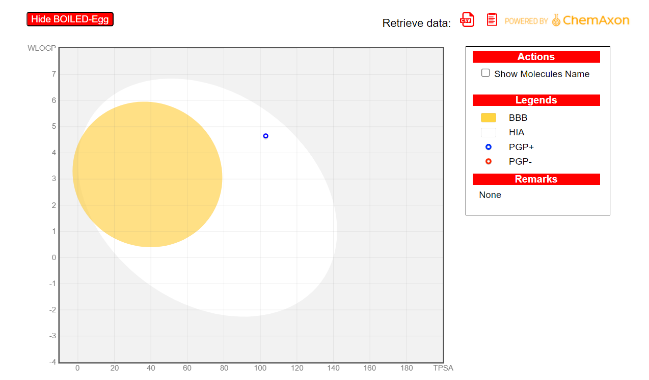 |
| PDFC41 | PDFC42 |
| 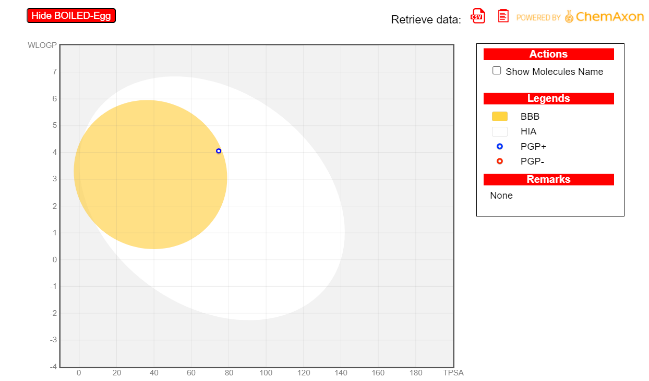 | 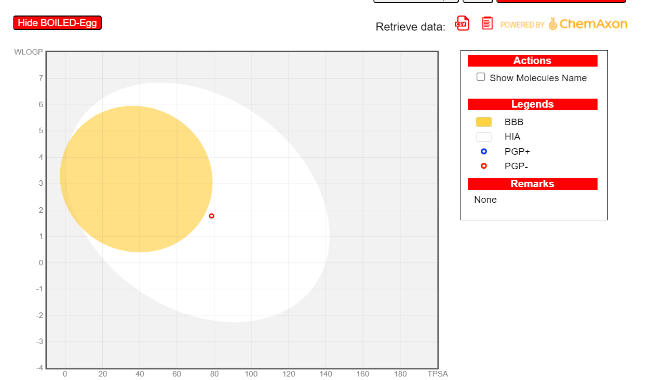 |
| PDFC43 | PDFC44 |
| 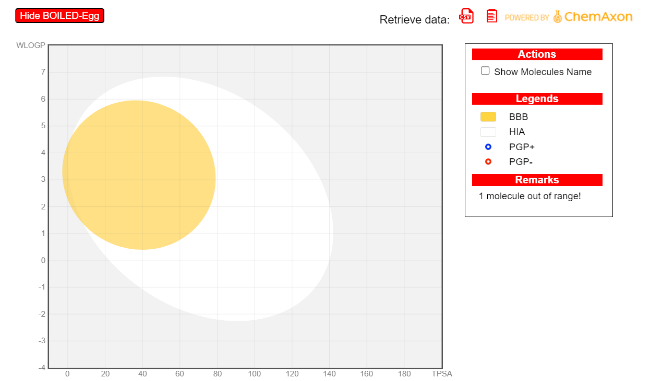 | 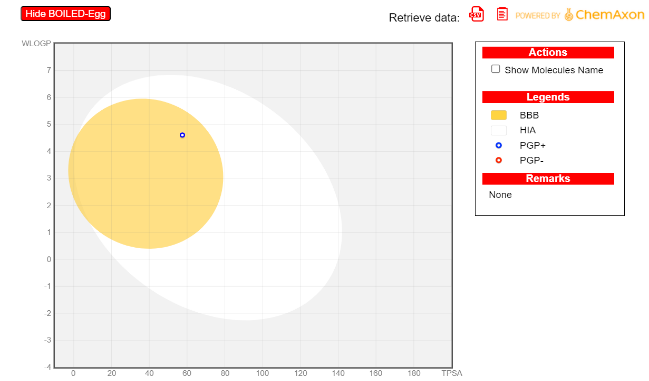 |
| PDFC45 | PDFC46 |
| 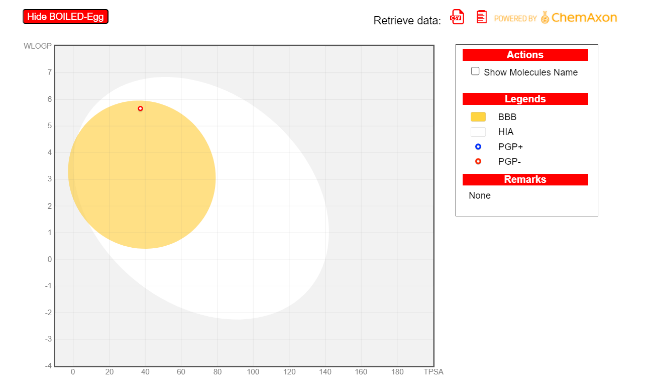 | 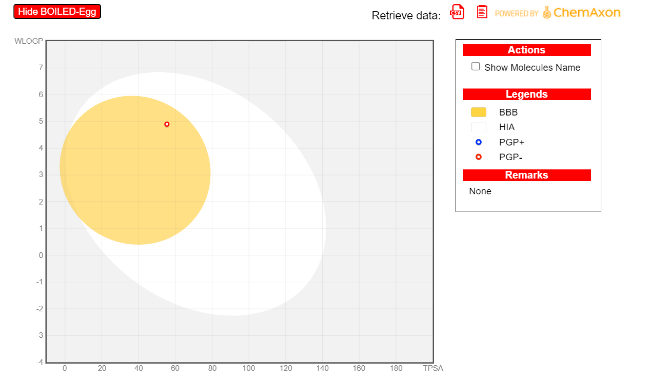 |
| PDFC47 | PDFC48 |
| 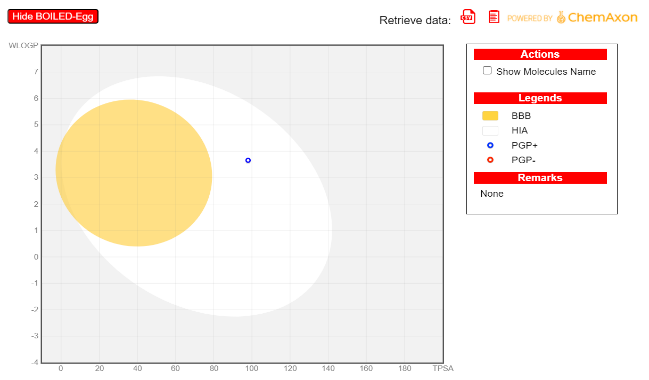 | 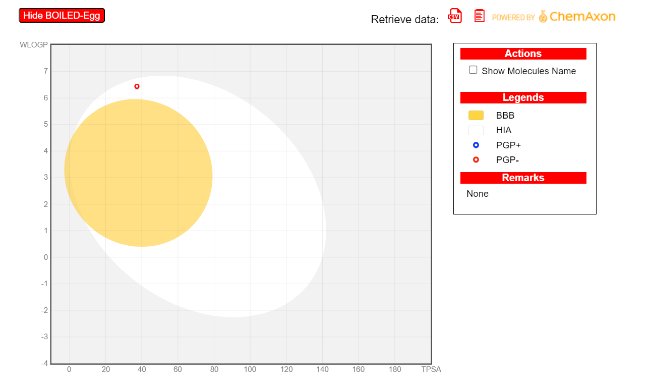 |
| PDFC49 | Carbamazepine |
| 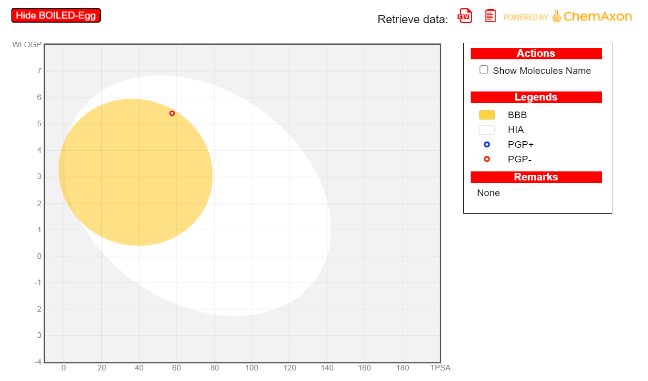 | 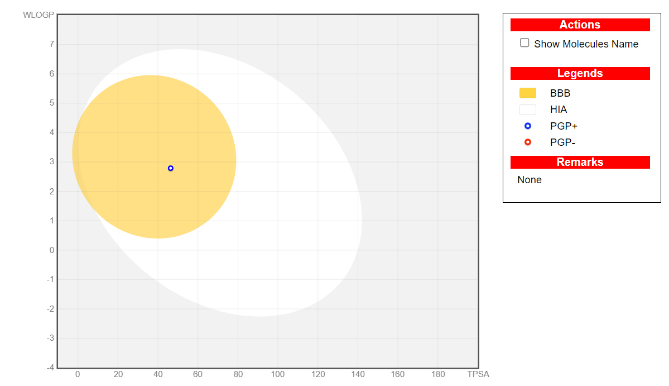 |
| Diazepam | Vigabatrin |
| 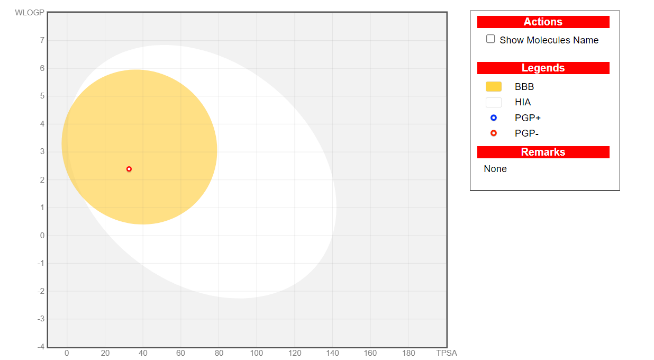 | 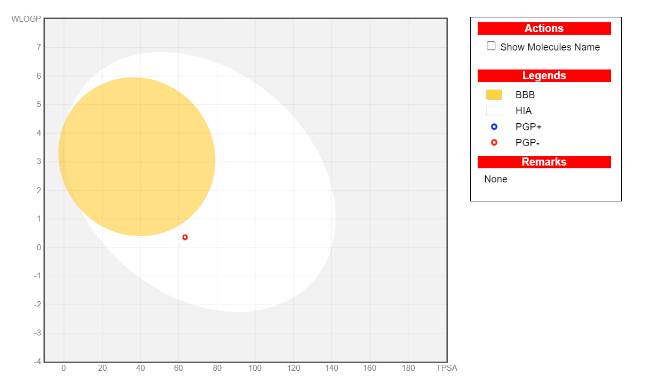 |

**Fig. S2.** BOILED-Egg models prediction for GI absorption and permeability of the BBB for phytoconstituents of dabai fruit
