# Supplementary material for: Exploration of Canarium odontophyllum fruit phytoconstituents as potential candidates against epilepsy using in silico studies
Source: J Genet Eng Biotechnol. 2025 Aug 26;23(4):100561. doi: 10.1016/j.jgeb.2025.100561 (PMC12409388; doi:10.1016/j.jgeb.2025.100561)
Supplement: Supplementary Data 5 [file mmc5.docx]

**Table S1.**

Structures of putatively identified compounds from the hydro-alcoholic extract of *C. odontophyllum* fruits by LC-MS analysis

| **No** | **Compound names & Structures** | **No** | **Compound names & Structures** |
| --- | --- | --- | --- |
| 1 | Nigerose (Sakebiose) (PDFC1)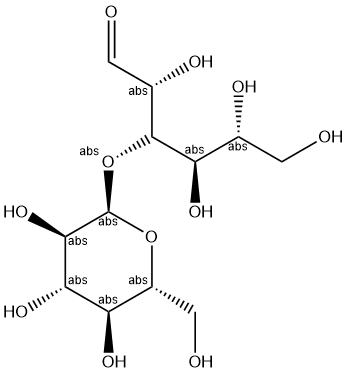 | 2 | 2-(beta-D-Glucosyl)-sn-glycerol  (PDFC2)  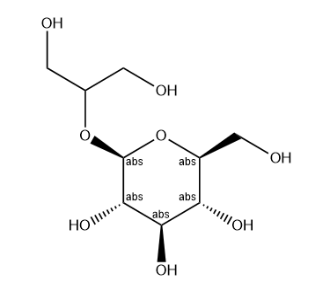 |
| 3 | Neuraminic acid (PDFC3)  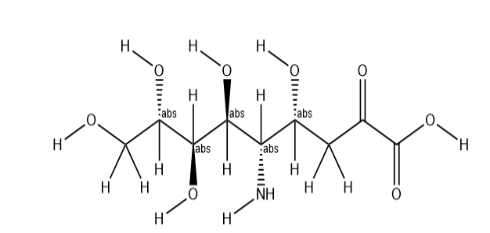 | 4 | 9Z,11E,13-Tetradecatrienal (PDFC4)  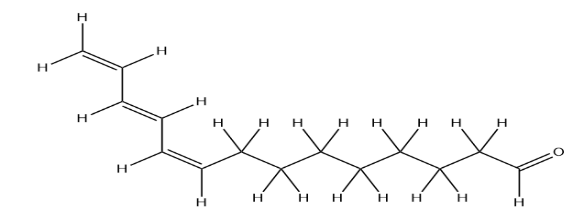 |
| 5 | 3'-Hydroxytrimethoprim (PDFC5)  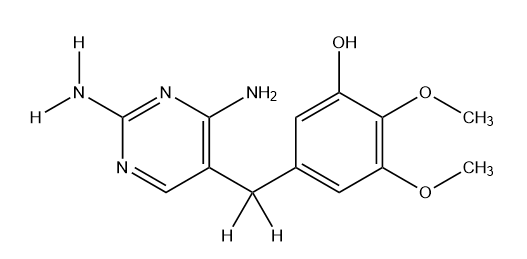 | 6 | Leu Pro (PDFC6)  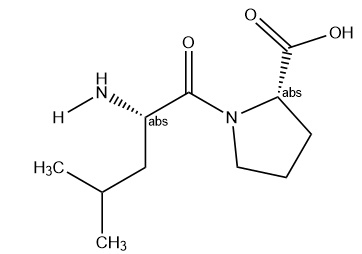 |
| 7 | Tranylcypromine glucuronide (PDFC7)  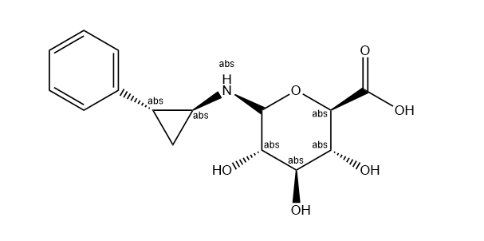 | 8 | cis-Zeatin (PDFC8)  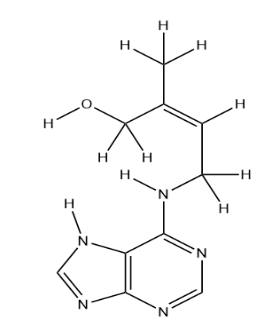 |
| 9 | Asn Tyr Thr (PDFC9)  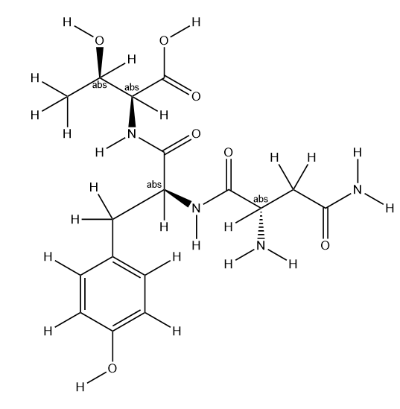 | 10 | Abscisate (PDFC10)  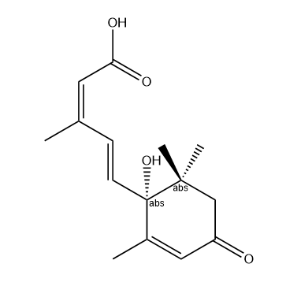 |
| 11 | 4-Methylesculetin (PDFC11)  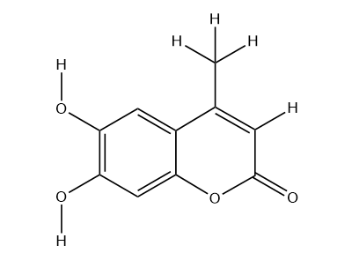 | 12 | Val-Val-OH (PDFC12)  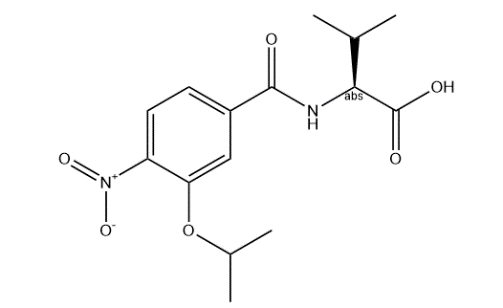 |
| 13 | 1-Methyl-4-nitro-5-(S-gluctathionyl) imidazole (PDFC13)  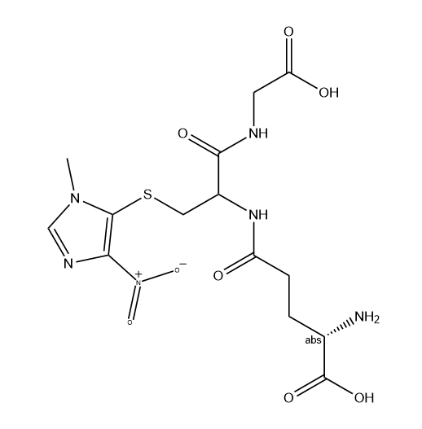 | 14 | 3,5,7,2',5'-Pentahydroxyflavone (PDFC14)  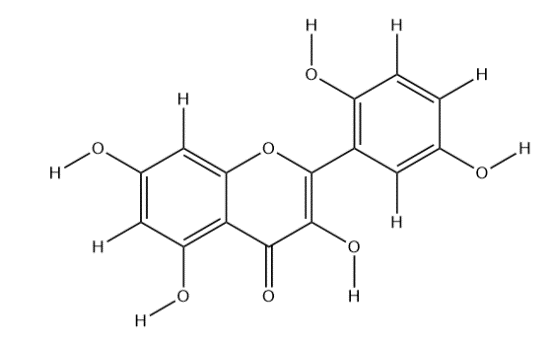 |
| 15 | 5,7,2'-Trihydroxy 7-glucoside (PDFC15)  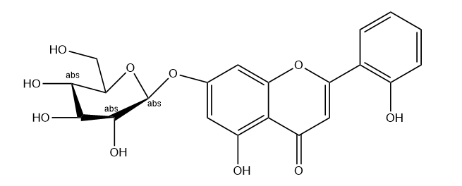 | 16 | Neovitexin (PDFC16)  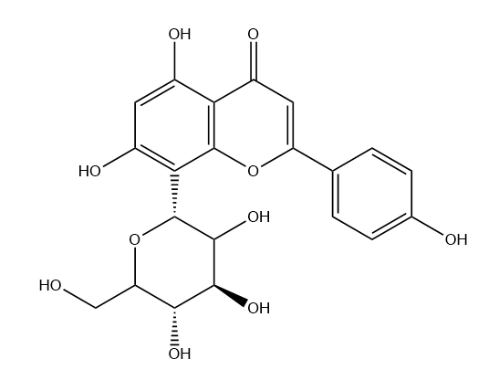 |
| 17 | Mefenamic acid metabolite (b-D-glucopyranuronic acid, 1-[2-[(2,3-dimethylphenyl)amino]benzoate]) (PDFC17)  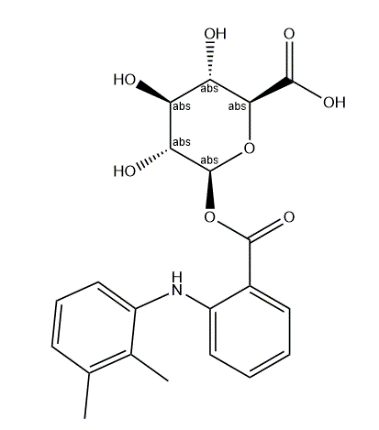 | 18 | Colnelenic acid (PDFC18)  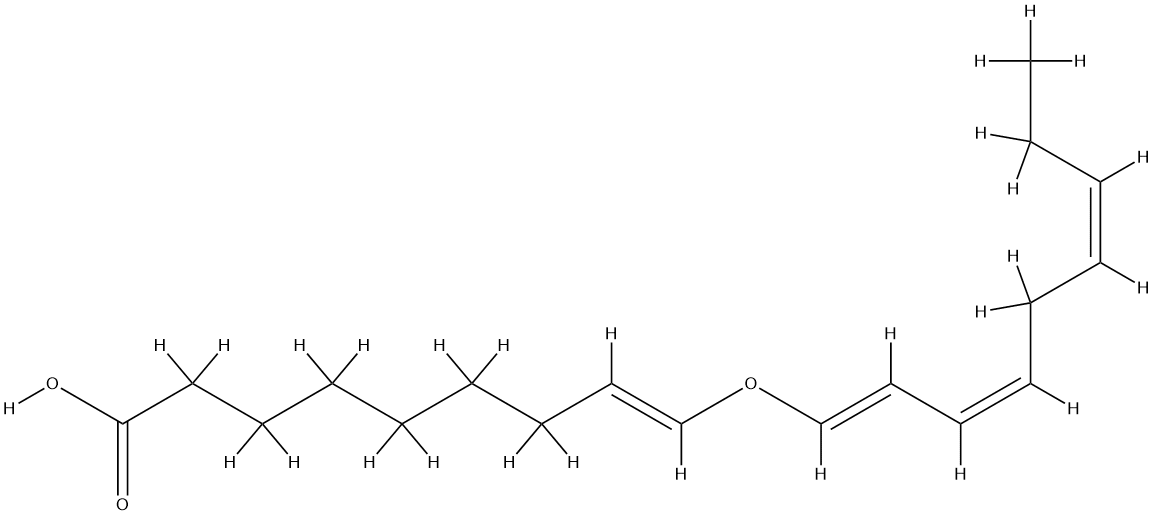 |
| 19 | 9-hydroperoxy-12,13-epoxy-10-octadecenoic acid (PDFC19)  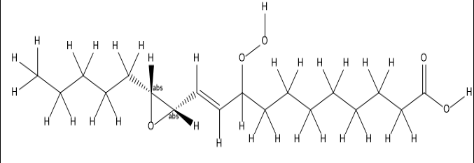 | 20 | Betaxolol (PDFC20)  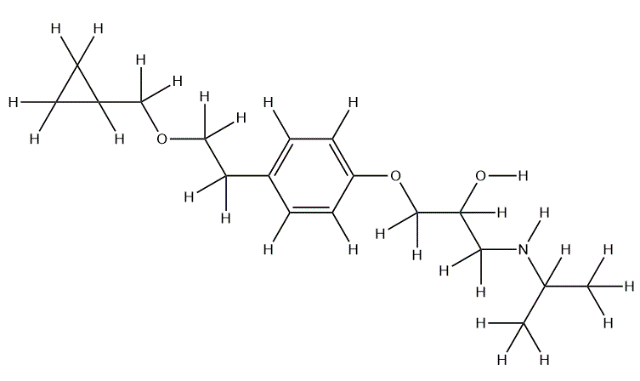 |
| 21 | 8,11-Octadecadiynoic acid (PDFC21)  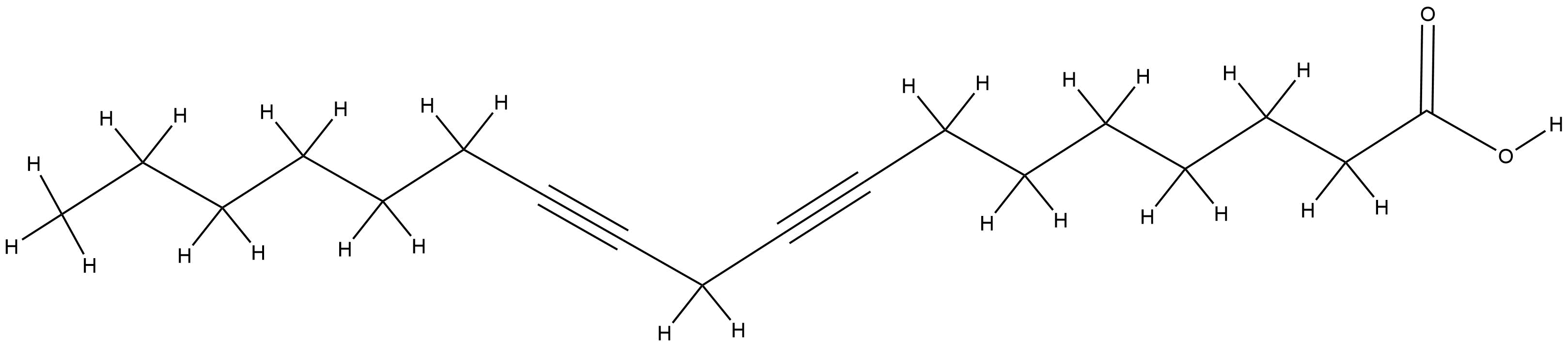 | 22 | Levuglandin E2 (PDFC22)  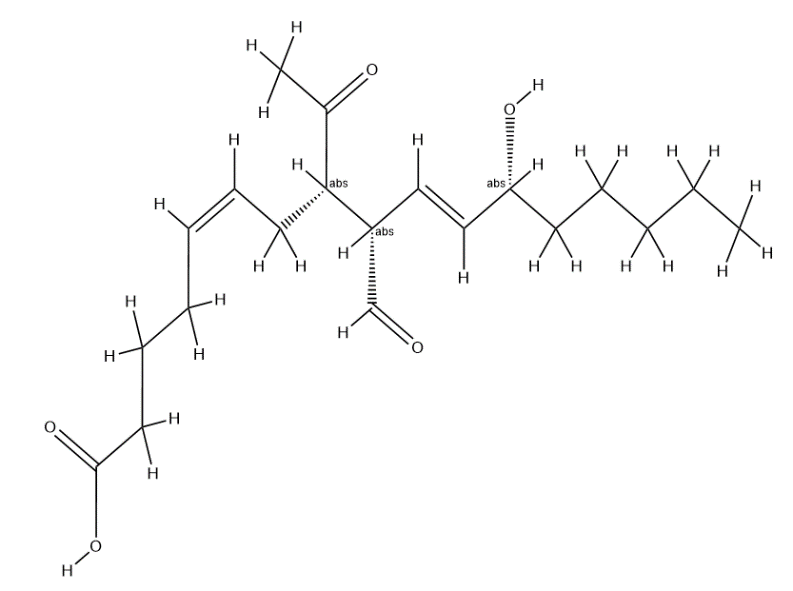 |
| 23 | 9S,10S,11R-trihydroxy-12Z-octadecenoic acid (PDFC23)  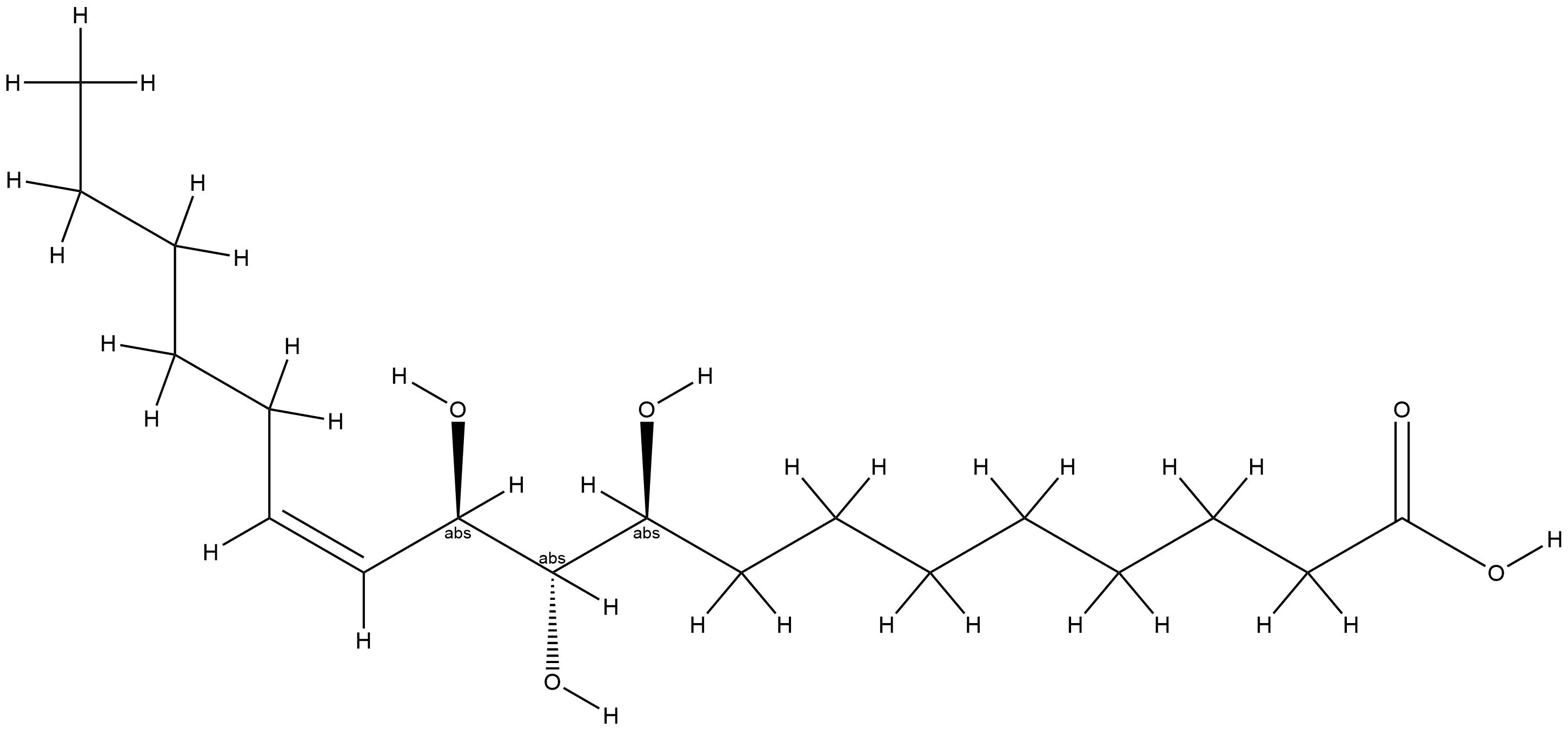 | 24 | 12,13S-epoxy-9Z,11-octadecadienoic acid (PDFC24)  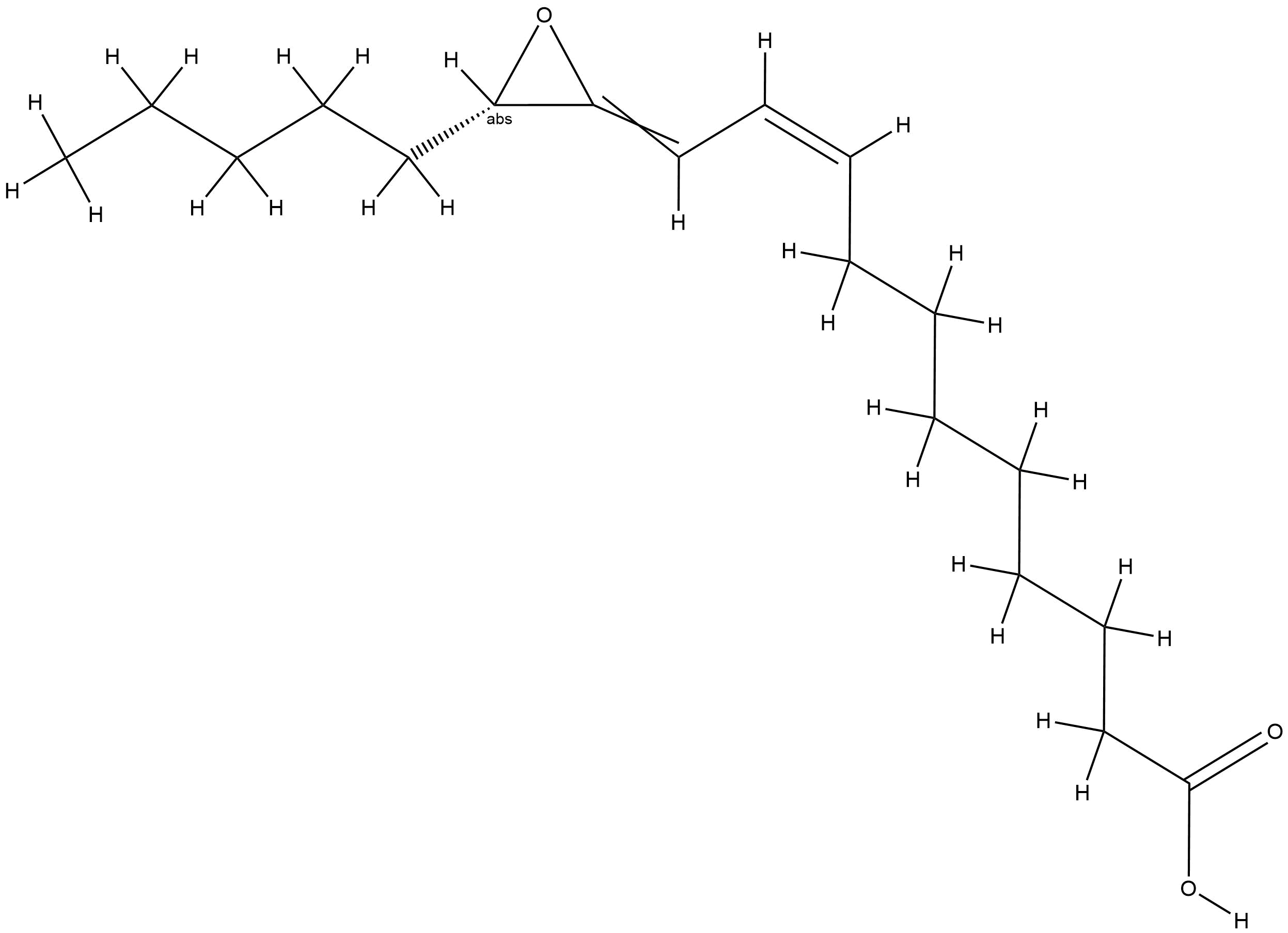 |
| 25 | Hinokiflavone (PDFC25)  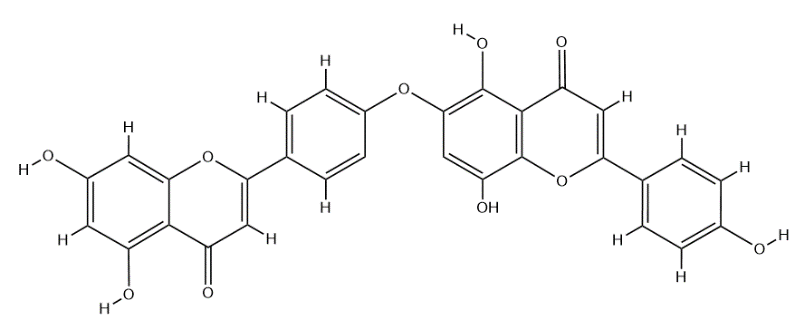 | 26 | 4-oxo-9Z,11Z,13E,15E-octadecatetraenoic acid (PDFC26)  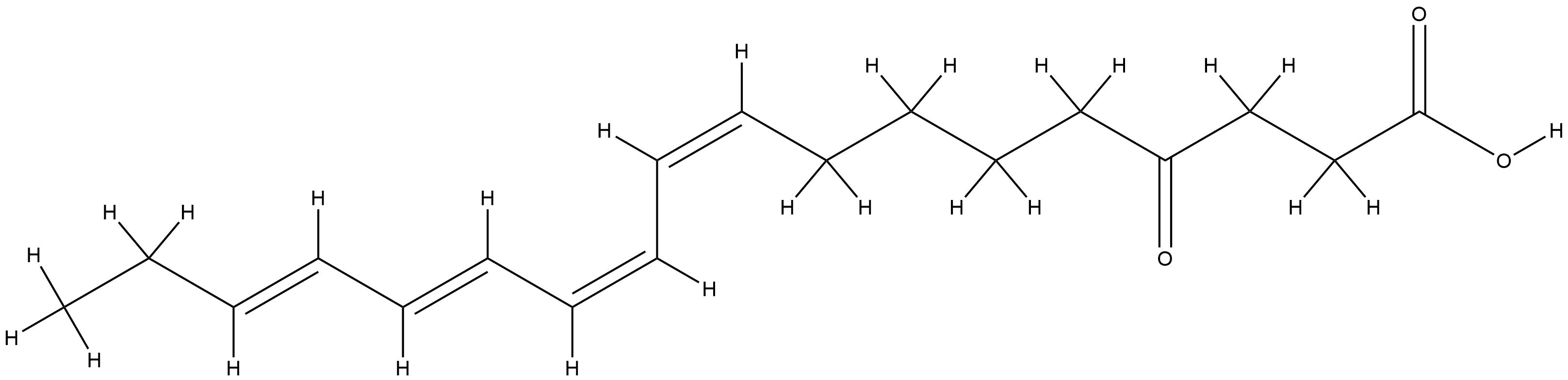 |
| 27 | 19-Norandrostenedione (PDFC27)  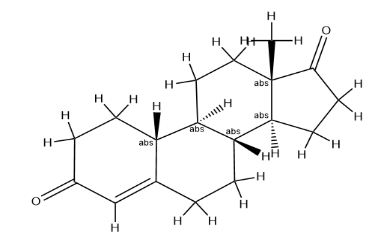 | 28 | trans-EKODE-(E)-Ib (PDFC28)  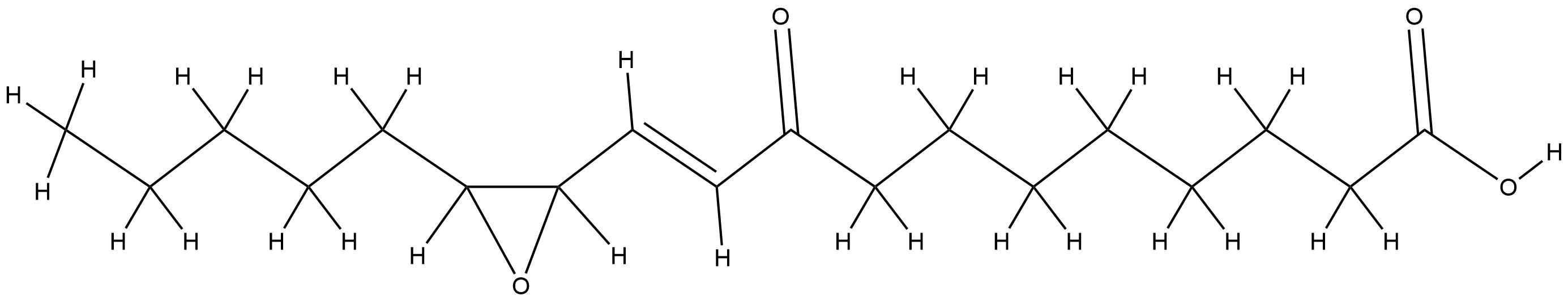 |
| 29 | 5-Oxo-ETE-d7 (PDFC29)  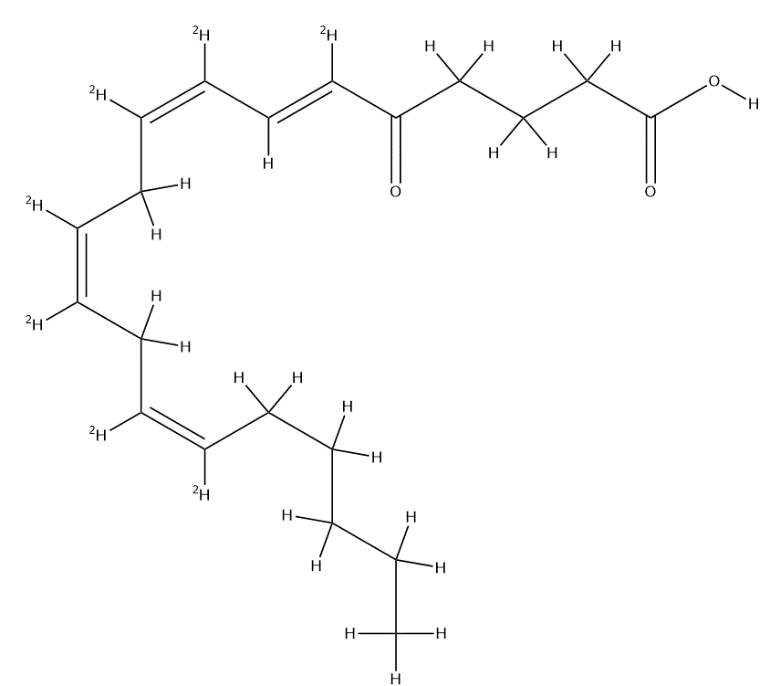 | 30 | Asn Asn Arg (PDFC30)  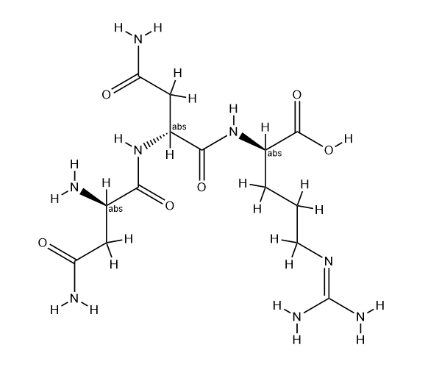 |
| 31 | Bavachromanol (PDFC31)  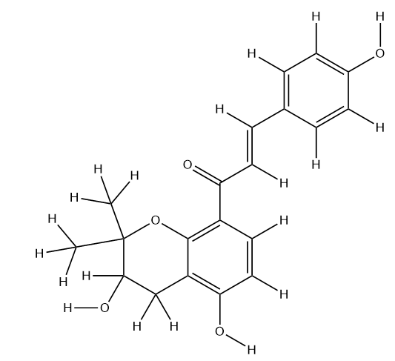 | 32 | Kanzonol B (PDFC32)  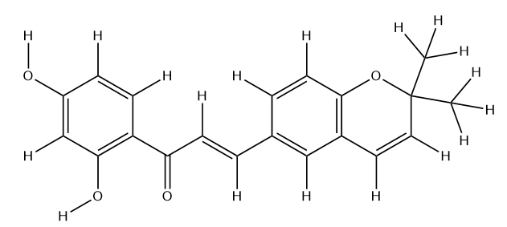 |
| 33 | 9E,12Z,15Z-octadecatrienoic acid (PDFC33)  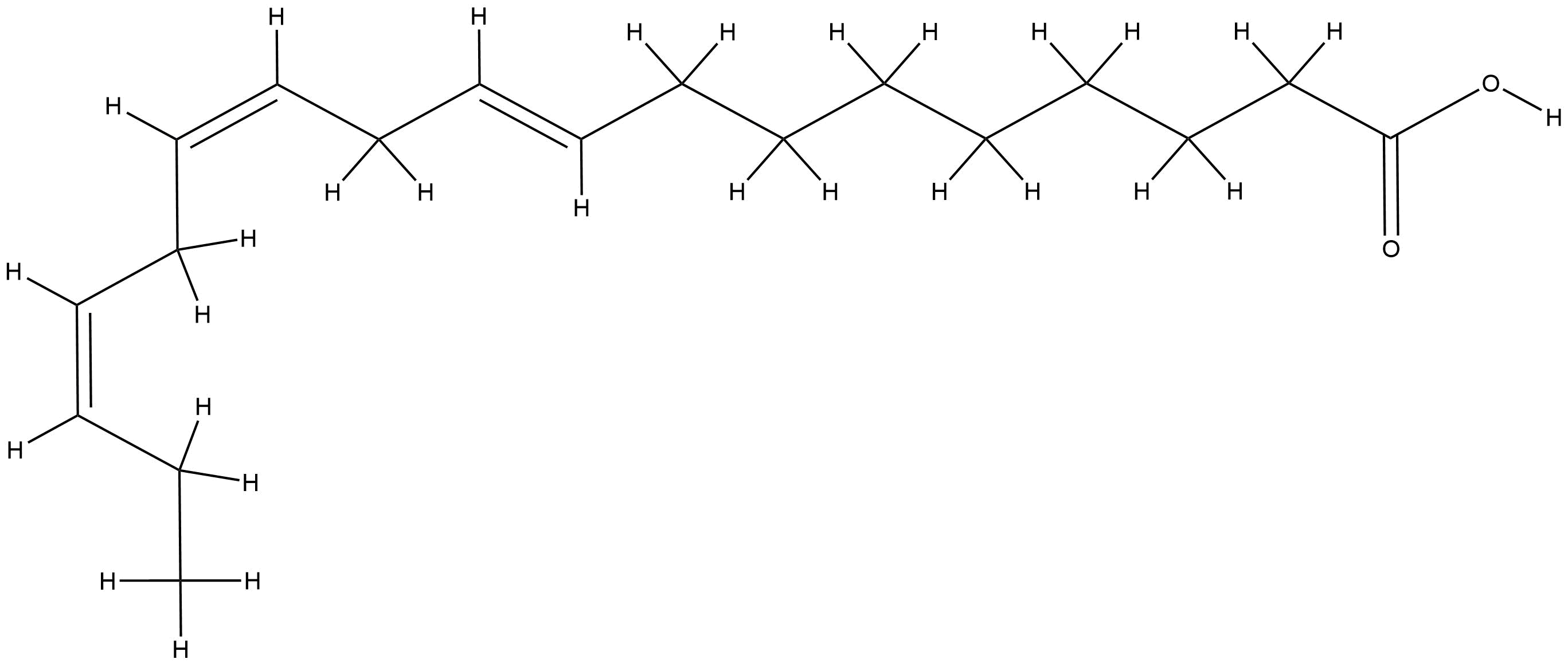 | 34 | Dihomo-PGI2 (PDFC34)  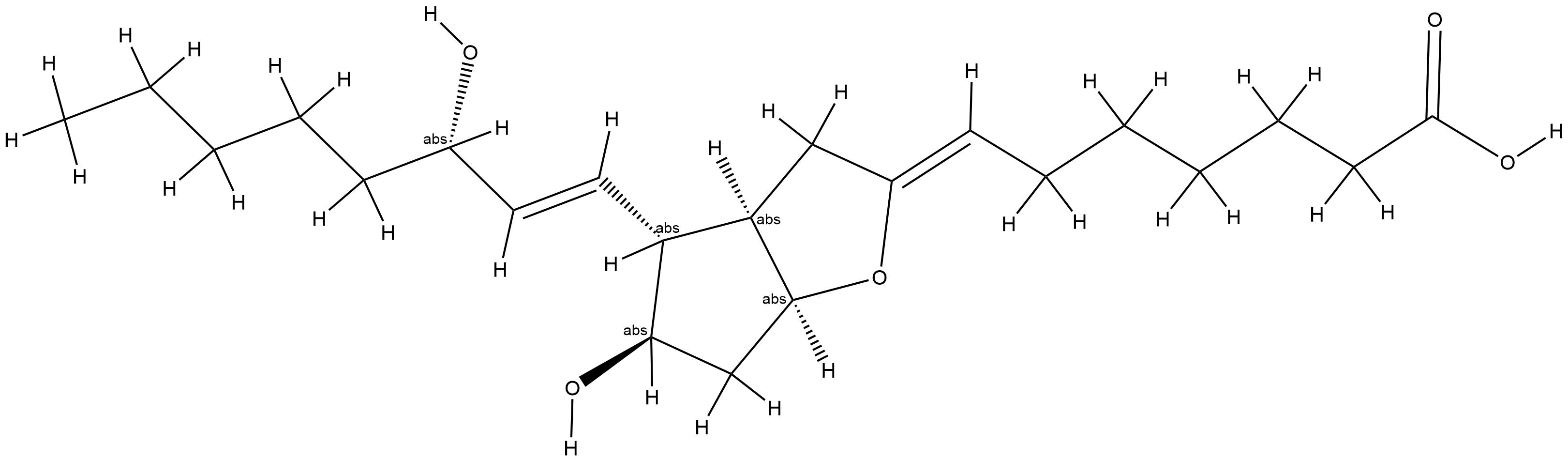 |
| 35 | 9,13-dihydroxy-10-ethoxy-11-octadecenoic acid (PDFC35)  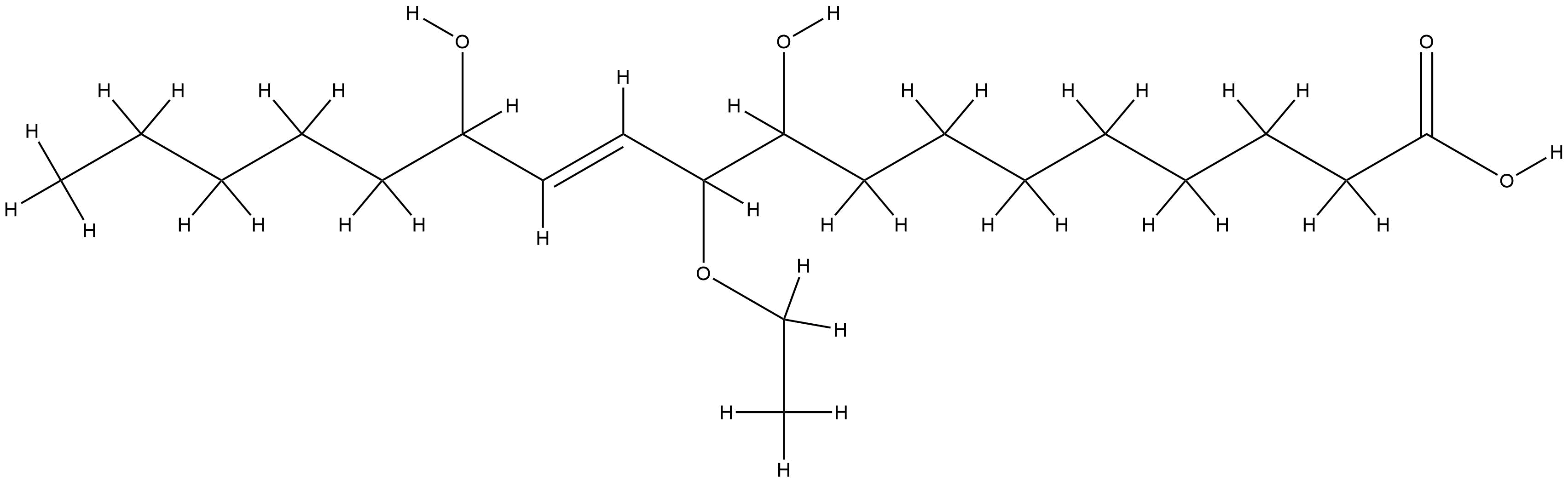 | 36 | 7-Methoxychromone (PDFC36)  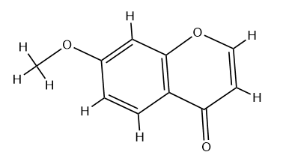 |
| 37 | Clobetasol propionate (PDFC37)  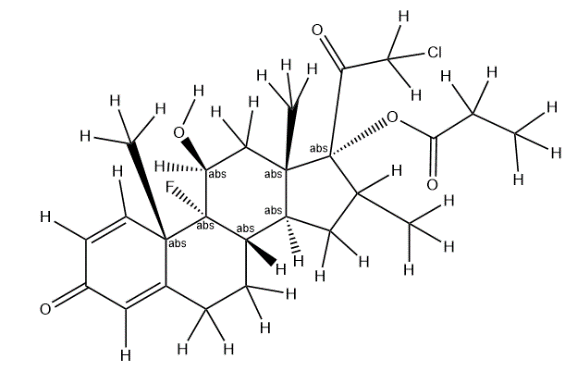 | 38 | 2-butyl-3-[[2′-(1H-tetrazol-5-yl)[1,1′-biphenyl]-4-yl]methyl]-1,3-diazaspiro[4.4]non-1-en-4-one (PDFC38)  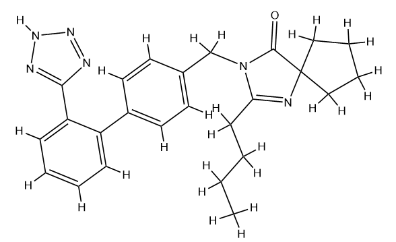 |
| 39 | Arg Thr Phe (PDFC39)  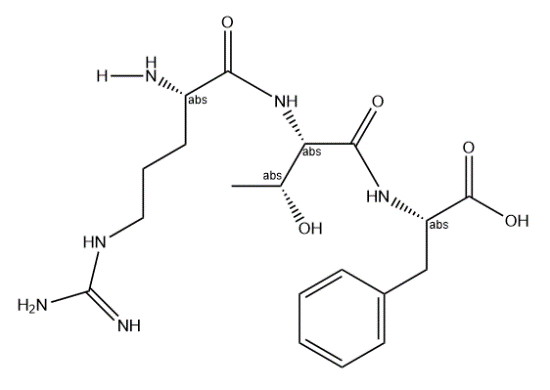 | 40 | 5-Hydroxyfluvastatin (PDFC40)  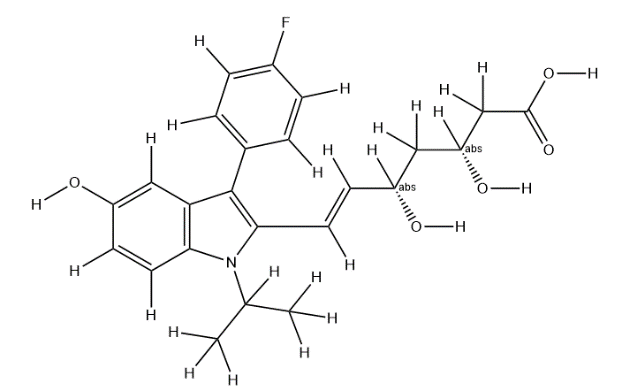 |
| 41 | 15-epi-15-A2t-IsoP (PDFC41)  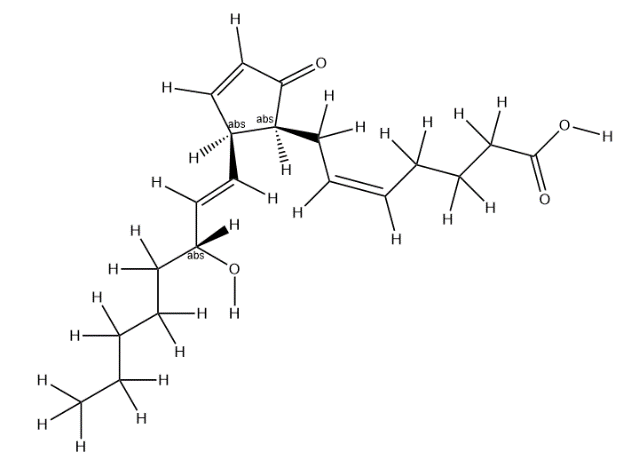 | 42 | L-Glutamic acid dibutyl ester (PDFC42)  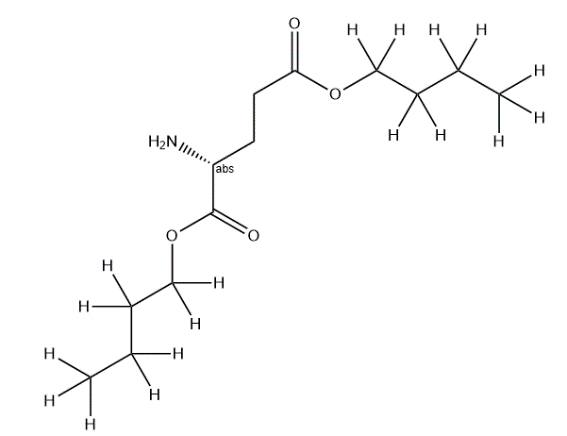 |
| 43 | Mayolene-18 (PDFC43)  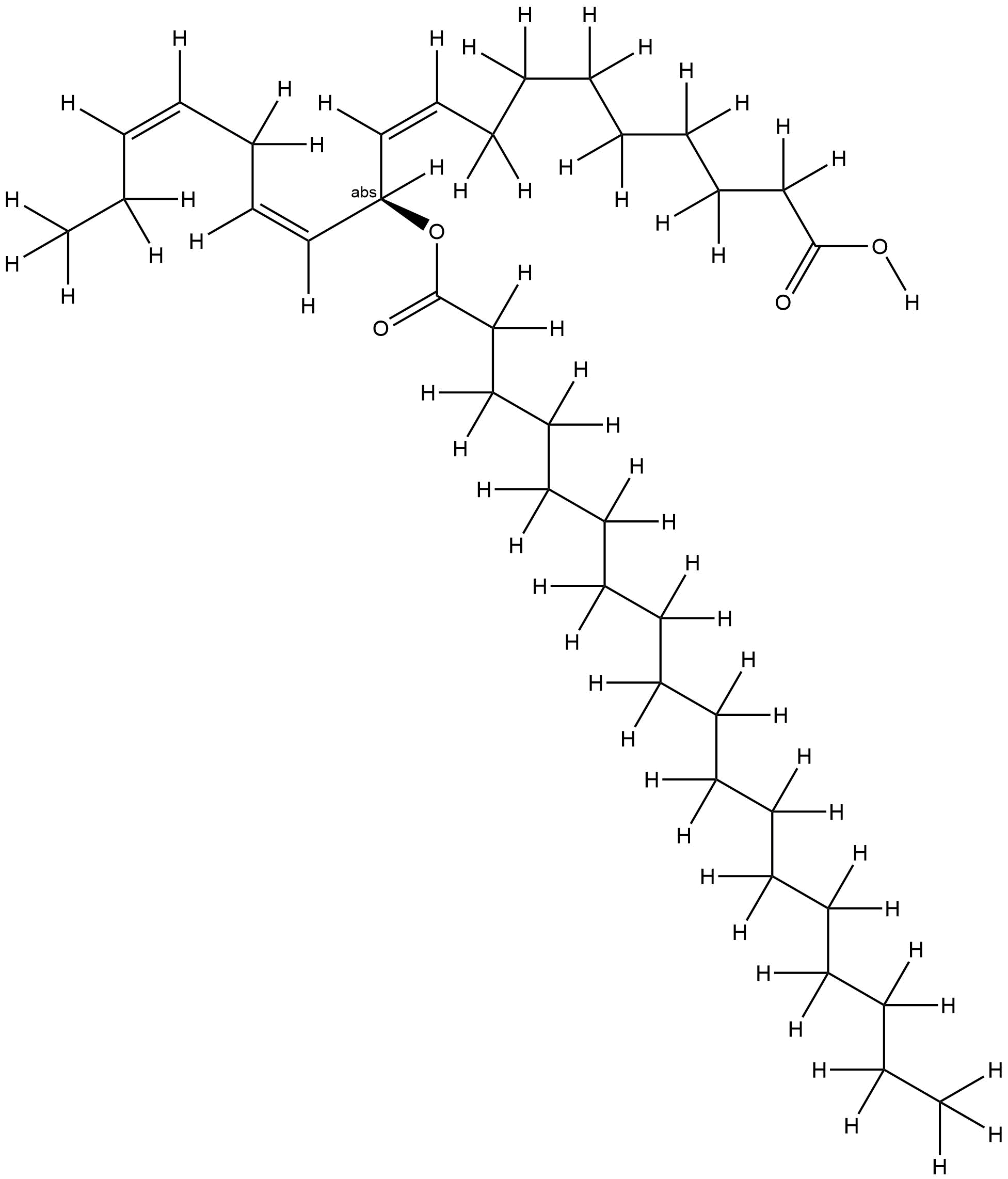 | 44 | 12-Hydroxy-10-octadecynoic acid (PDFC44)  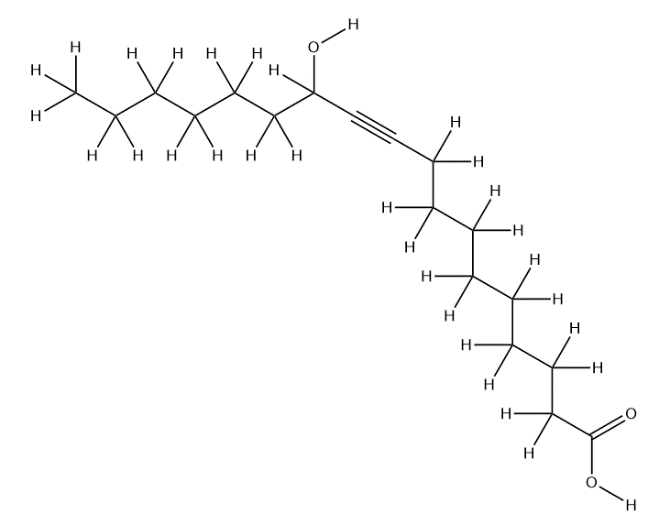 |
| 45 | 9E,12Z,15Z-Octadecatrienoic acid (PDFC45)  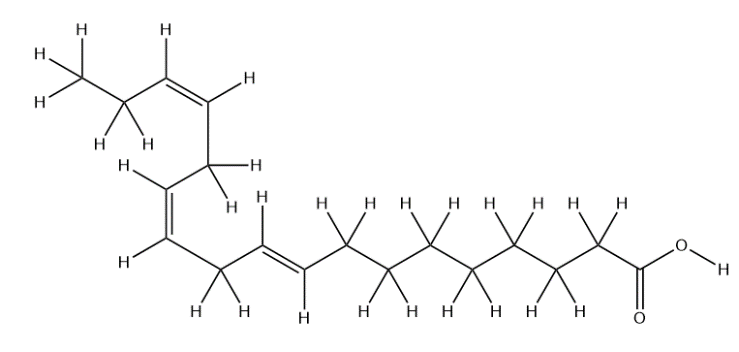 | 46 | N-Hexadecanoyl-L-homoserine lactone (PDFC46)  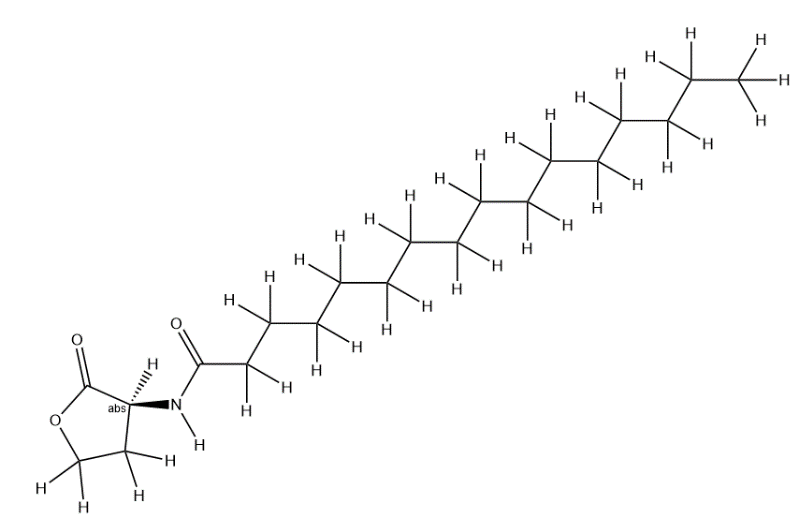 |
| 47 | 17,20-Dimethylprostaglandin F1α (PDFC47)  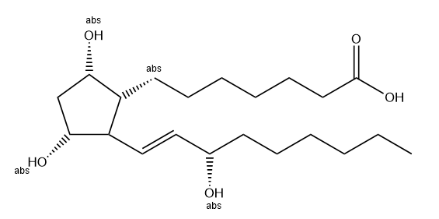 | 48 | 7,11,14-Eicosatrienoic acid  (PDFC48)  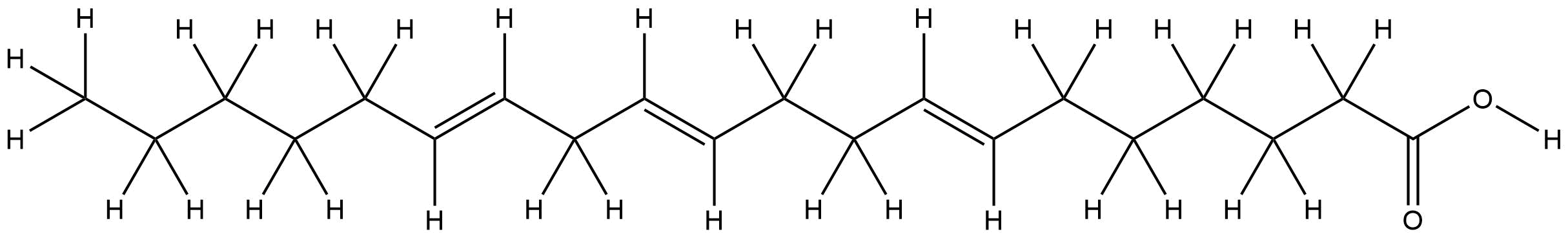 |
| 49 | 8(S)-HETrE (PDFC49)  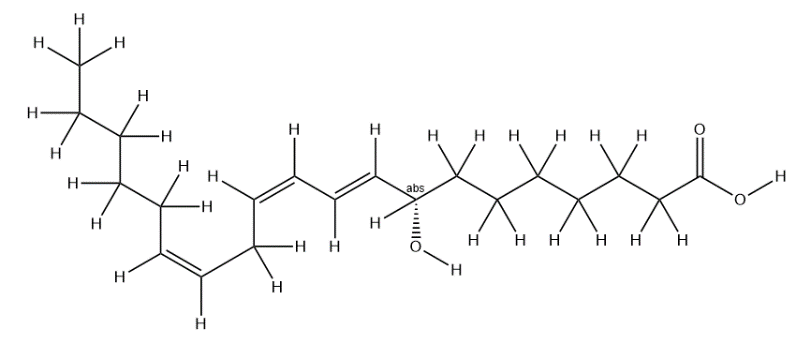 | 50 | Mitoxantrone (NDFC1)  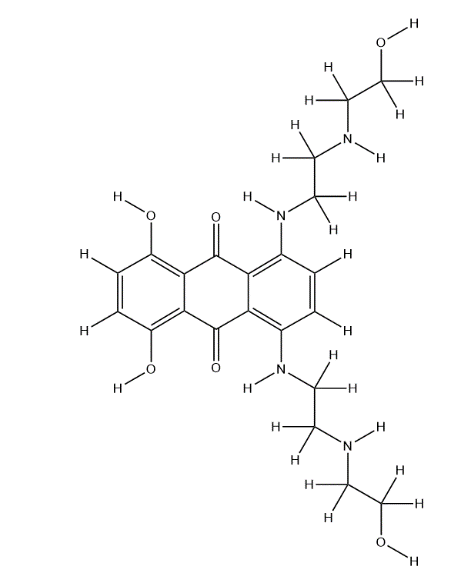 |
| 51 | Gln Ala Tyr (NDFC2)  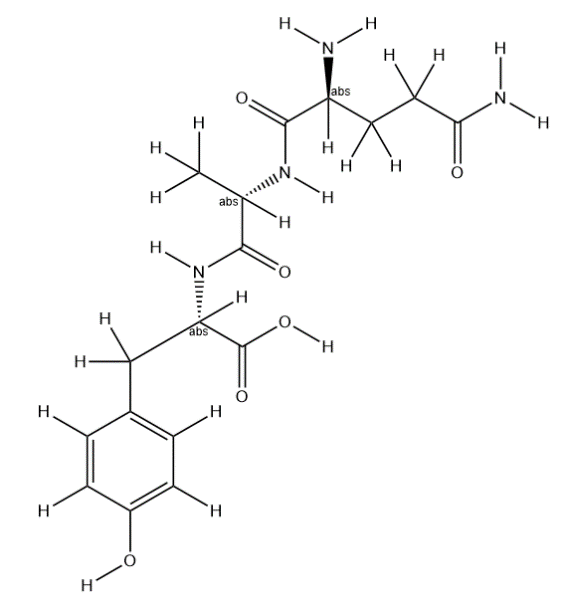 | 52 | Robustaflavone (NDFC3)  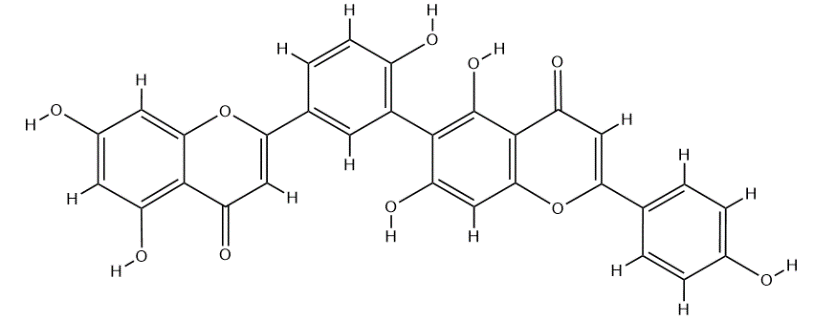 |
| 53 | 12-Octadecenoic acid, 9,10,18-trihydroxy-; 9,10,18 Trihydroxyoctadec-12-enoic acid (NDFC4)  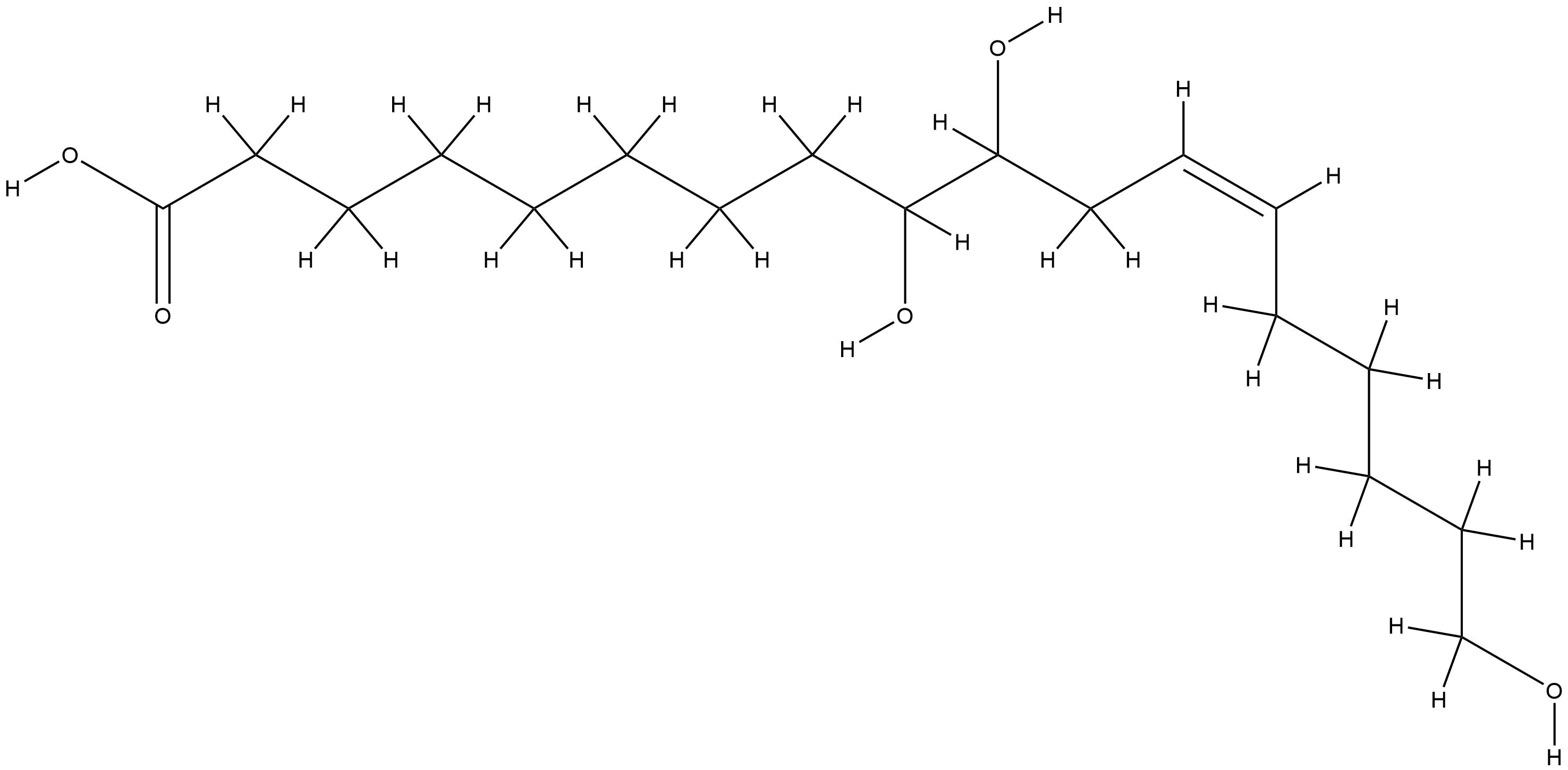 | 54 | 9-hydroperoxy-12,13-epoxy 10-octadecenoic acid (NDFC5)  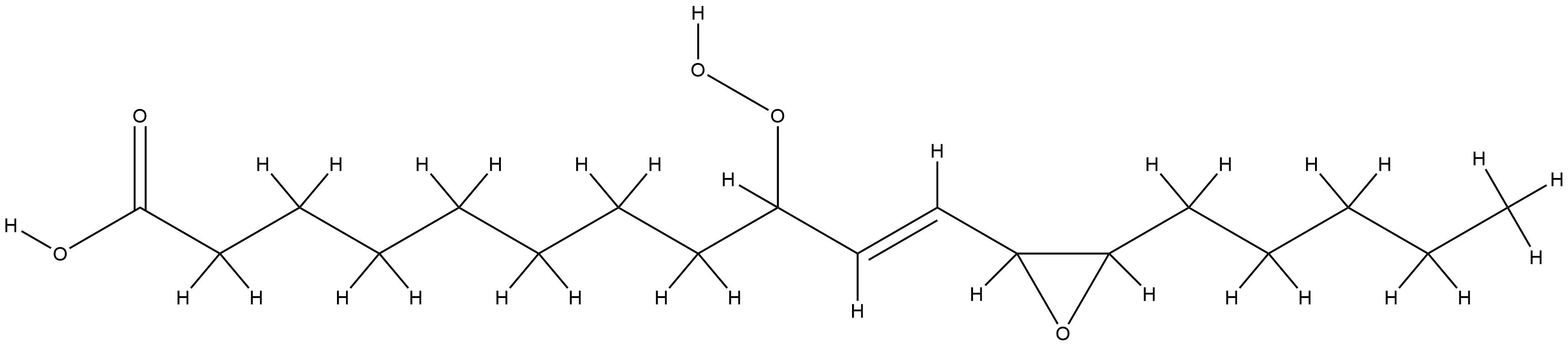 |
| 55 | 9,13-dihydroxy-10-ethoxy-11 octadecenoic acid (NDFC6)  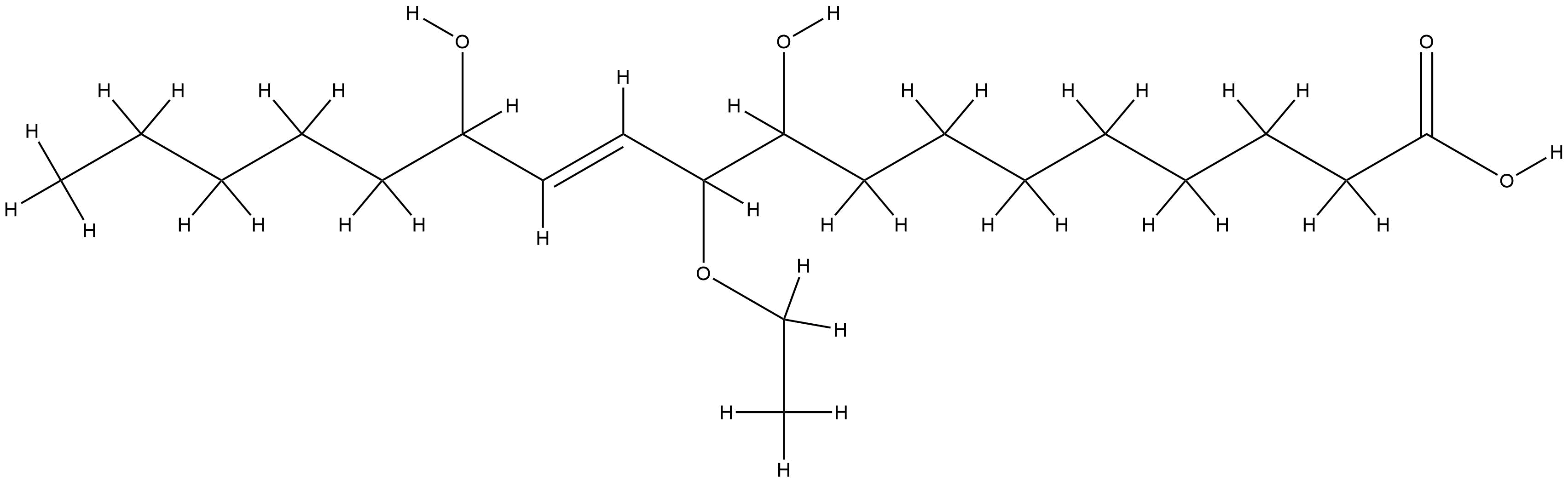 | 56 | 2-(cyclohexylcarbonyl)-1,2,3,6,7,11b-hexahydro-4H-pyrazino[2,1-a]isoquinolin-4-one (NDFC7)  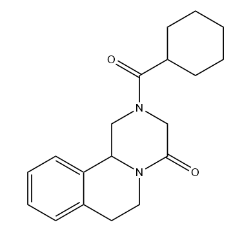 |
| 57 | Lamioside (NDFC8)  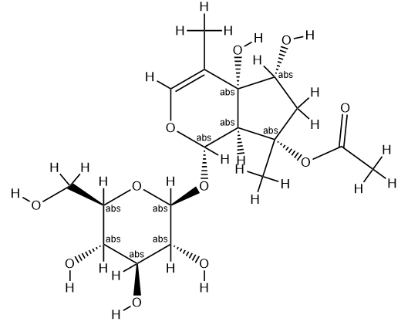 | 58 | Ajmaline (NDFC9)  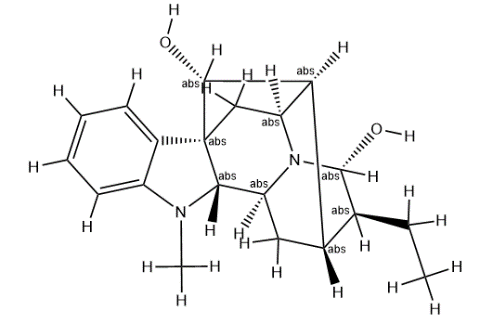 |
| 59 | 4,14-dihydroxy-octadecanoic acid (NDFC10)  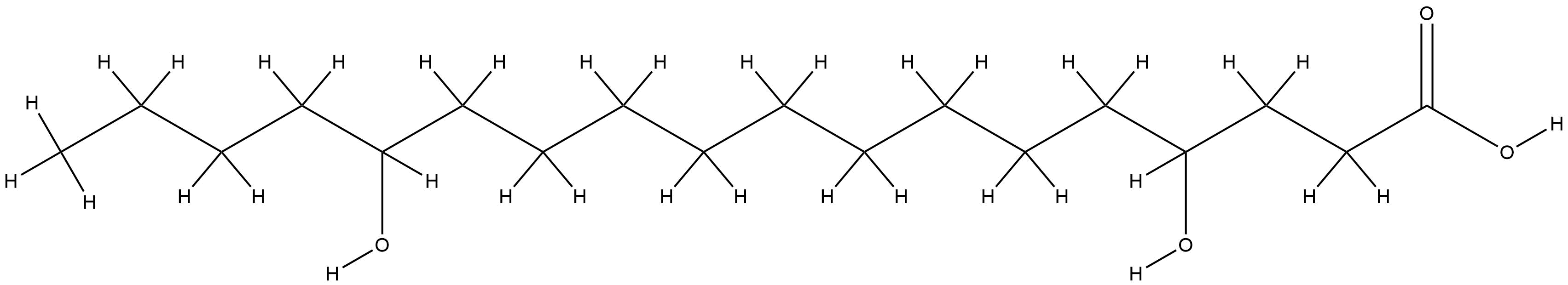 | 60 | 13(R)-HODE (NDFC11)  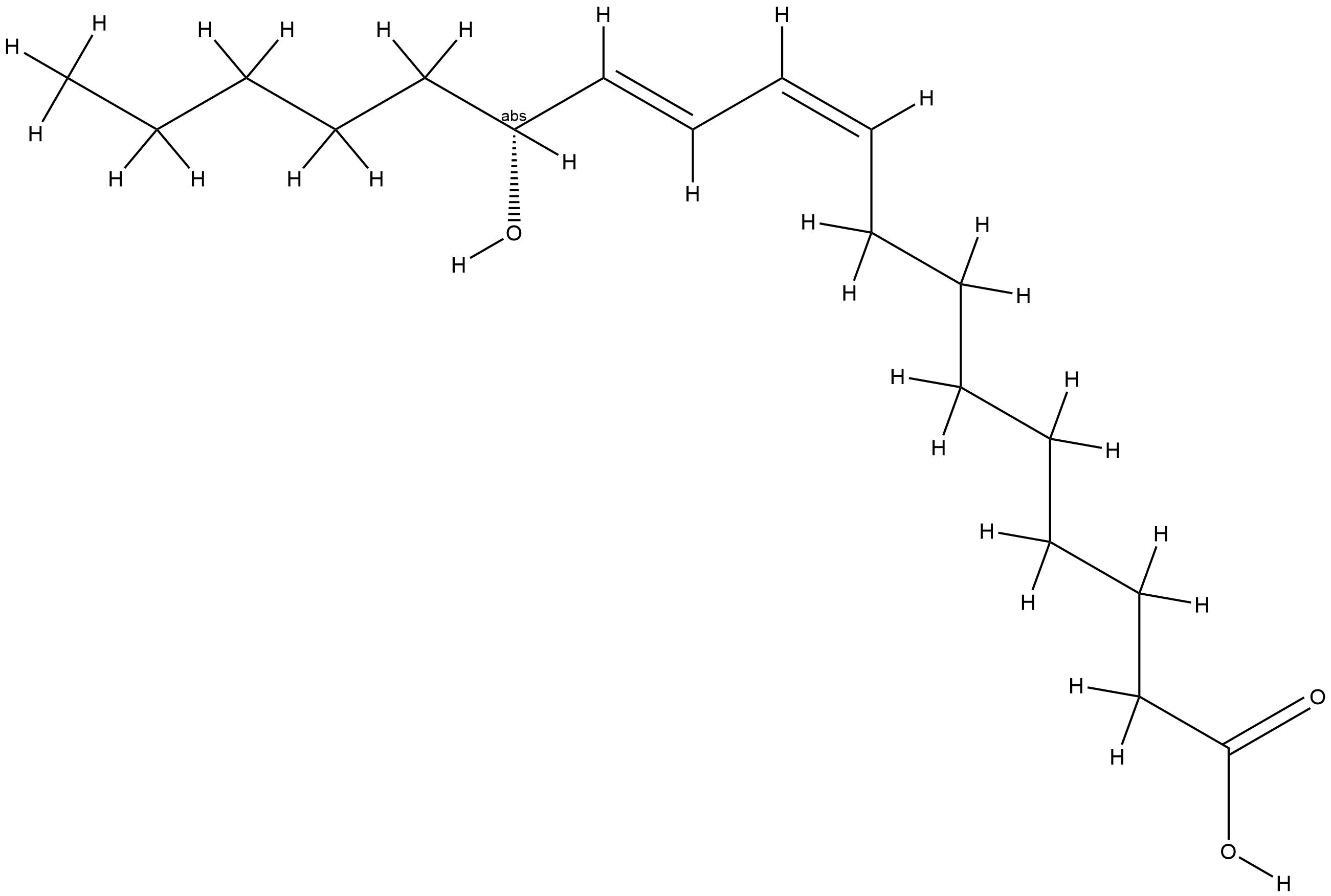 |
| 61 | 9S,10R-epoxy-stearic acid (NDFC12)  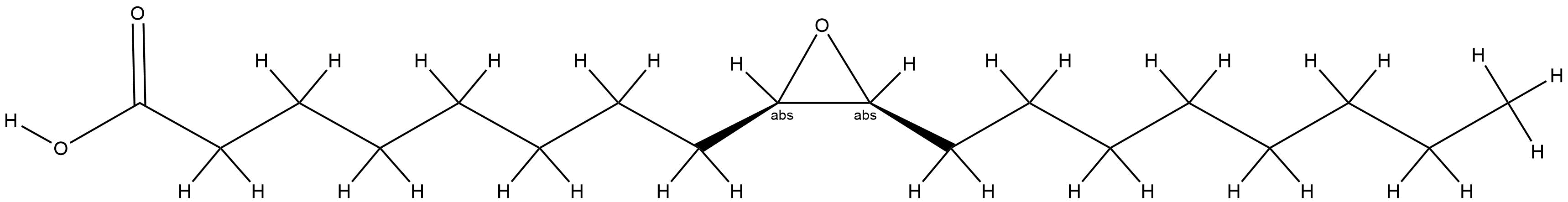 | 62 | (±)-N-{4-[2-hydroxy-3-(isopropylaminopropoxy]phenyl}acetamide (NDFC13)  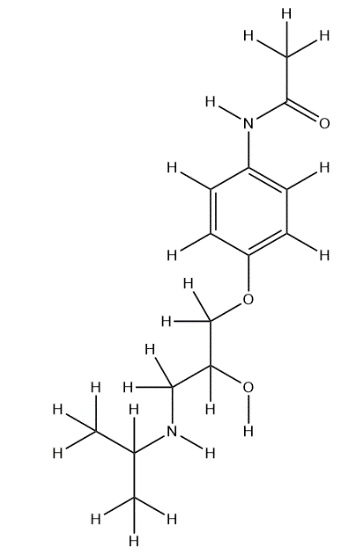 |
| 63 | 2-chloropalmitaldehyde (NDFC14)  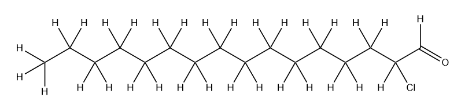 |  |  |

**Table S2**.

Phytoconstituents identified from the hydro-alcoholic extract of *C. odontophyllum* fruits by LC-MS/MS analysis.

| **Code** | **Exp m/z** | **Calc m/z** | **Ion mode** | **Diff (ppm)** | ***MS/MS*** |
| --- | --- | --- | --- | --- | --- |
| PDFC1 | 381.0806 | 381.0794 | (M+K)^+^ | -3.12 | 108, 153, 201, 219 |
| PDFC2 | 293.0638 | 293.0633 | (M+K)^+^ | -1.46 | 118, 175, 220, 234 |
| PDFC3 | 268.1014 | 268.1027 | (M+H)^+^ | 4.72 | 103, 115, 137, 267 |
| PDFC4 | 229.1560 | 229.1563 | (M+Na)^+^ | 1.17 | 114, 126, 151, 210 |
| PDFC5 | 294.1559 | 294.1561 | (M+NH_4_)^+^ | 0.52 | 115, 230, 258, 293 |
| PDFC6 | 229.1558 | 229.1547 | (M+H)^+^ | -4.95 | 107, 124, 168, 210 |
| PDFC7 | 310.1294 | 310.1285 | (M+H)^+^ | -2.88 | 120, 178, 244, 292 |
| PDFC8 | 220.1193 | 220.1193 | (M+H)^+^ | 0.12 | 103, 124, 166, 202 |
| PDFC9 | 435.1274 | 435.1277 | (M+K)^+^ | 0.53 | 127, 151, 333, 375 |
| PDFC10 | 265.1447 | 265.1434 | (M+H)^+^ | -4.61 | 135, 175, 229, 247 |
| PDFC11 | 193.0503 | 193.0495 | (M+H)^+^ | -4.07 | 107, 157, 175, 178 |
| PDFC12 | 325.1407 | 325.1394 | (M+H)^+^ | -3.94 | 116, 175, 192, 307 |
| PDFC13 | 433.1157 | 433.1136 | (M+H)^+^ | -4.78 | 283, 337, 397, 415 |
| PDFC14 | 303.0510 | 303.0499 | (M+H)^+^ | -3.59 | 130, 153, 178, 302 |
| PDFC15 | 433.1142 | 433.1129 | (M+H)^+^ | -2.85 | 145, 289, 397, 415 |
| PDFC16 | 433.1139 | 433.1129 | (M+H)^+^ | -2.21 | 127, 145, 289, 415 |
| PDFC17 | 435.1774 | 435.1762 | (M+NH_4_)^+^ | -2.70 | 132, 287, 361, 417 |
| PDFC18 | 293.2122 | 293.2111 | (M+H)^+^ | -3.76 | 123, 195, 239, 292 |
| PDFC19 | 346.2601 | 346.2588 | (M+NH_4_)^+^ | -3.64 | 137, 173, 214, 347 |
| PDFC20 | 308.2231 | 308.2220 | (M+H)^+^ | -3.36 | 109, 217, 245, 290 |
| PDFC21 | 277.2172 | 277.2162 | (M+H)^+^ | -3.76 | 135, 175, 259, 276 |
| PDFC22 | 353.2316 | 353.2323 | (M+H)^+^ | 1.78 | 137, 278, 281, 335 |
| PDFC23 | 353.2310 | 353.2298 | (M+Na)^+^ | -3.23 | 164, 292, 335, 351 |
| PDFC24 | 295.2279 | 295.2268 | (M+H)^+^ | -3.90 | 125, 249, 276, 294 |
| PDFC25 | 539.0984 | 539.0973 | (M+H)^+^ | -2.17 | 113, 277, 403, 537 |
| PDFC26 | 291.1968 | 291.1955 | (M+H)^+^ | -4.56 | 123, 147, 163, 290 |
| PDFC27 | 273.1862 | 273.1849 | (M+H)^+^ | -4.60 | 105, 137, 237, 255 |
| PDFC28 | 311.2228 | 311.2217 | (M+H)^+^ | -3.53 | 139, 195, 292, 310 |
| PDFC29 | 343.2974 | 343.2973 | (M+NH_4_)^+^ | -0.46 | 104, 171, 239, 241 |
| PDFC30 | 403.2034 | 403.2048 | (M+H)^+^ | 3.53 | 105,152, 252, 385 |
| PDFC31 | 341.1395 | 341.1384 | (M+H)^+^ | -3.33 | 133, 163, 263, 323 |
| PDFC32 | 323.1288 | 323.1278 | (M+H)^+^ | -3.01 | 107, 135, 165, 293 |
| PDFC33 | 279.2328 | 279.2319 | (M+H)^+^ | -3.45 | 109, 181, 223, 278 |
| PDFC34 | 381.2625 | 381.2636 | (M+H)^+^ | 2.75 | 115, 155, 317, 379 |
| PDFC35 | 381.2624 | 381.2611 | (M+Na)^+^ | -3.38 | 121, 195, 268, 363 |
| PDFC36 | 177.0555 | 177.0546 | (M+H)^+^ | -4.78 | 121, 149, 158, 177 |
| PDFC37 | 467.1988 | 467.1995 | (M+H)^+^ | 1.42 | 105, 224, 407, 467 |
| PDFC38 | 467.1970 | 467.1956 | (M+K)^+^ | -3.06 | 105, 170, 287, 385 |
| PDFC39 | 445.2164 | 445.2170 | (M+Na)^+^ | 1.31 | 105, 177, 224, 385 |
| PDFC40 | 445.2139 | 445.2133 | (M+NH_4_)^+^ | -1.19 | 105, 194, 295, 385 |
| PDFC41 | 335.2207 | 335.2217 | (M+H)^+^ | 2.80 | 111, 195, 289, 334 |
| PDFC42 | 277.2111 | 277.2122 | (M+NH_4_)^+^ | 3.85 | 107, 123, 207, 276 |
| PDFC43 | 561.4895 | 561.4877 | (M+H)^+^ | -3.22 | 111, 181, 321, 559 |
| PDFC44 | 297.2438 | 297.2424 | (M+H)^+^ | -4.74 | 109, 141, 278, 296 |
| PDFC45 | 279.2331 | 279.2319 | (M+H)^+^ | -4.55 | 109, 169, 243, 278 |
| PDFC46 | 340.2848 | 340.2846 | (M+H)^+^ | -0.60 | 137, 179, 280, 322 |
| PDFC47 | 385.2934 | 385.2949 | (M+H)^+^ | 3.84 | 123, 163, 222, 338 |
| PDFC48 | 307.2635 | 307.2632 | (M+H)^+^ | -1.20 | 109, 163, 223, 243 |
| PDFC49 | 323.2582 | 323.2581 | (M+H)^+^ | -0.32 | 135, 173, 277, 305 |
| NDFC1 | 443.1930 | 443.1936 | (M-H)^-^ | 1.33 | 101, 113, 161, 407 |
| NDFC2 | 379.1622 | 379.1623 | (M-H)^-^ | 0.28 | 100, 217, 317, 319 |
| NDFC3 | 537.0821 | 537.0827 | (M-H)^-^ | 1.20 | 117, 135, 203, 443 |
| NDFC4 | 329.2348 | 329.2333 | (M-H)^-^ | -4.28 | 100, 121, 171, 211 |
| NDFC5 | 327.2177 | 327.2177 | (M-H)^-^ | 0.12 | 129, 155, 227, 309 |
| NDFC6 | 357.2643 | 357.2646 | (M-H)^-^ | 0.90 | 171, 211, 293, 356 |
| NDFC7 | 311.1762 | 312.1843 | (M-H)^-^ | 3265.71 | 139, 171, 223, 267 |
| NDFC8 | 479.1764 | 479.1770 | (M+CH_3_COO)^-^ | 1.24 | 103, 255, 262, 478 |
| NDFC9 | 325.1925 | 326.2000 | (M-H)^-^ | 3124.14 | 119, 183, 279, 307 |
| NDFC10 | 315.2549 | 315.2541 | (M-H)- | -2.43 | 123, 139, 171, 201 |
| NDFC11 | 295.2276 | 295.2279 | (M-H)- | 0.99 | 113, 171, 277, 294 |
| NDFC12 | 297.2430 | 297.2435 | (M-H)- | 1.66 | 111, 127, 183, 233 |
| NDFC13 | 265.1562 | 266.1636 | (M-H)^-^ | 3824.53 | 109, 165, 167, 263 |
| NDFC14 | 309.1761 | 309.1757 | (M+Cl)^-^ | -1.00 | 138, 170, 183, 197 |

Deviation from the database (library) in ppm

**Table S3.**

Category of compounds detected in LC-MS/MS analysis

| **Category** | **Compound Name** |
| --- | --- |
| Flavonoids | 3,5,7,2',5'-Pentahydroxyflavone |
|  | Neovitexin |
|  | Hinokiflavone |
|  | Bavachromanol |
|  | Kanzonol B |
|  | Robustaflavone |
| Terpenoids | Abscisate |
|  | Lamioside |
| Phenolic | 3'-Hydroxytrimethoprim |
|  | Mitoxantrone |
| Alkaloids | Ajmaline |
| Carbohydrates | Nigerose (Sakebiose) |
|  | 2-(beta-D-Glucosyl)-sn-glycerol |
|  | Neuraminic acid |
|  | 5,7,2'-Trihydroxy 7-glucoside |
|  | Mefenamic acid Metabolite (b-D-Glucopyranuronic acid, 1-[2-[(2,3-dimethylphenyl)amino]benzoate]) |
| Lipids | 9Z,11E,13-Tetradecatrienal |
|  | Colnelenic acid |
|  | 9-hydroperoxy-12,13-epoxy-10-octadecenoic acid |
|  | 8,11-octadecadiynoic acid |
|  | Levuglandin E2 |
|  | 9S,10S,11R-trihydroxy-12Z-octadecenoic acid |
|  | 12,13S-epoxy-9Z,11-octadecadienoic acid |
|  | 4-oxo-9Z,11Z,13E,15E-octadecatetraenoic acid |
|  | 19-Norandrostenedione |
|  | trans-EKODE-(E)-Ib |
|  | 5-Oxo-ETE-d7 |
|  | 9E,12Z,15Z-octadecatrienoic acid |
|  | Dihomo-PGI2 |
|  | 9,13-dihydroxy-10-ethoxy-11-octadecenoic acid |
|  | 15-epi-15-A2t-IsoP |
|  | Mayolene-18 |
|  | 12-hydroxy-10-octadecynoic acid |
|  | 9E,12Z,15Z-octadecatrienoic acid |
|  | N-hexadecanoyl-L-Homoserine lactone |
|  | 7,11,14-Eicosatrienoic acid |
|  | 8(S)-HETrE |
|  | 12-Octadecenoic acid, 9,10,18-trihydroxy-; 9,10,18-Trihydroxyoctadec-12-enoic acid |
|  | 9-hydroperoxy-12,13-epoxy-10-octadecenoic acid |
|  | 9,13-dihydroxy-10-ethoxy-11-octadecenoic acid |
|  | 13(R)-HODE |
|  | 4,14-dihydroxy-octadecanoic acid |
|  | 9S,10R-epoxy-stearic acid |
|  | 2-chloropalmitaldehyde |
| Peptides | Leu Pro |
|  | Asn Tyr Thr |
|  | Val-Val-OH |
|  | Asn Asn Arg |
|  | Arg Thr Phe |
|  | Gln Ala Tyr |
| Other compounds | Tranylcypromine glucuronide |
|  | Betaxolol |
|  | Clobetasol propionate |
|  | 17,20-dimethyl Prostaglandin F1α |
|  | *N*-[4-[2-hydroxy-3-(propan-2-ylamino)propoxy]phenyl]acetamide |
|  | cis-Zeatin |
|  | 4-Methylesculetin |
|  | 1-Methyl-4-nitro-5-(S-Gluctathionyl) Imidazole |
|  | 7-Methoxychromone |
|  | 2-butyl-3-[[4-[2-(2*H*-tetrazol-5-yl)phenyl]phenyl]methyl]-1,3-diazaspiro[4.4]non-1-en-4-one |
|  | 5-hydroxyfluvastatin |
|  | L-Glutamic acid dibutyl ester |
|  | 2-(cyclohexanecarbonyl)-3,6,7,11*b*-tetrahydro-1*H*-pyrazino[2,1-a]isoquinolin-4-one |

**Table S4.**

Standard drugs used for each protein

| **Receptor/ Enzyme** | **PDB ID** | **Standard**  **drugs** |
| --- | --- | --- |
| Gamma-Aminobutyric acid GABA(A) receptor | 1gnu | Diazepam |
| Gamma-aminobutyrate aminotransferase (GABA-T) | 1sff | Carbamazepine |
| Human mitochondrial branched chain aminotransferase (BCATm) | 2a1h | Carbamazepine |
| Human Voltage-gated Sodium Channel, brain isoform (Nav1.2)  (HVGSC) | 2kav | Carbamazepine |
| α-amino-3-hydroxy-5-methyl-4-isoxazolepropionic acid (AMPA) receptor | 3dp4 | Carbamazepine |
| Human Cyclin Dependent Kinase 2 | 1pxo | Carbamazepine |
| Leucine-rich glioma inactivated 1 (LGI1) | 5y30 | Carbamazepine |
| Human arginase I | 3thj | Carbamazepine |

**Table S5.**

Results of virtual screening using PyRx

| **Receptor/ Enzyme** | **PDB ID** | **Virtual Docking Result** | | | | | |
| --- | --- | --- | --- | --- | --- | --- | --- |
|  |  | **Top 1** | | **Top 2** | | **Top 3** | |
|  |  | **PDB ID Score**  **(kcal/mol)** | | **PDB ID Score**  **(kcal/mol)** | | **PDB ID Score**  **(kcal/mol)** | |
| Gamma-aminobutyric acid (A) receptor | 1gnu | PDFC27 | -7.4 | NDFC7 | -6.87 | NDFC3 | -6.46 |
| Gamma-aminobutyrate aminotransferase | 1sff | PDFC32 | -8.99 | PDFC15 | -8.32 | PDFC25 | -8.07 |
| Human mitochondrial branched chain aminotransferase | 2a1h | PDFC10 | -8.68 | PDFC31 | -8.59 | PDFC32 | -8.49 |
| Human Voltage-gated Sodium Channel, brain isoform | 2kav | PDFC32 | -7.43 | NDFC9 | -7.18 | NDFC7 | -7.11 |
| α-amino-3-hydroxy-5-methyl-4-isoxazolepropionic acid receptor | 3dp4 | PDFC11  PDFC32 | -8.3 | PDFC14 | -7.6 | PDFC38 | -7.5 |
| Human cyclin dependent kinase 2 | 1pxo | PDFC25 | -9.85 | PDFC32 | -9.4 | PDFC16 | -9.21 |
| Leucine-rich glioma inactivated 1 | 5y30 | PDFC32 | -9.22 | NDFC3 | -8.97 | NDFC7 | -7.88 |
| Human arginase I | 3thj | PDFC39 | -7.22 | PDFC27 | -7.19 | PDFC5 | -6.66 |

**Table S6.**

Amino acid residues of proteins interacted with phytoconstituents of *C. odontophyllum* fruit and standard drugs

| **Receptor/ Enzyme PDB ID** | **Ligand** | **Bound crystal compound** | | | | |
| --- | --- | --- | --- | --- | --- | --- |
|  |  | **Hydrogen** | **Hydrophobic** | **van der Waals** | **Electrostatic** | **Others** |
| 1gnu | Standard  (Diazepam) | ARG65 | ARG65, ARG71, ARG71, ALA72, LYS66 | TYR61, PHE62, HIS69, LEU70 | - | - |
| 1gnu | Kanzonol B  (PDFC32) | LYS66, HIS69, HIS69 | ARG65, LYS66, ARG71, ARG71 | ALA72, PHE62 | ARG71 | - |
| 1sff | Crystallographic ligand  (4'-Deoxy-4'-Acetylamino-Pyridoxal-5'-Phosphate) | GLU211, GLY111, SER112, ARG141, ARG141, ASP239, TYR138, GLY111 | VAL241 | VAL115, HIS139, GLU206, GLN242, GLY210, ILE50, THR110, GLY140 | LYS268, LYS268 | - |
| 1sff | Standard  (Carbamazepine) | LYS268, GLU211 | VAL241, VAL241, VAL241, TYR138 | GLU206, ASP239, HIS139, GLY140, SER112, GLY111, THR110, ARG398, ILE50, GLN242 | - | - |
| 1sff | Kanzonol B  (PDFC32) | SER112, GLU211, ASP239, ASP239, GLY210 | VAL241, VAL241 | MET354, GLN209, GLN242, TYR138, HIS139, GLY140, GLY111, THR110, ARG141, LYS268, SER243, GLU240, PRO207. VAL208 | GLU206 | - |
| 2a1h | Crystallographic ligand  (Pyridoxal-5'-Phosphate) | ARG99, LYS202, VAL269, THR313, THR240, GLY268, SER311 | - | TYR207, GLU237, MET241, ASN242, LEU266, GLY312, VAL270 | ARG99, LYS202 | - |
| 2a1h | Standard  (Carbamazepine) | ARG99, ARG99, GLY77 | GLY312, GLY268, ALA314, VAL270 | LYS202, TYR141, GLU76, ASN96, VAL269, ARG271, ASN242, SER311, LEU266, MET241, THR240, THR313 | ARG99 | - |
| 2a1h | Kanzonol B  (PDFC32) | ARG99, SER311, GLY77 | GLY312, ALA314, MET241, VAL270 | THR313, LYS202, THR240, TYR173, GLN224, CYS315, GLY268, ASN242, ARG271, VAL269 | ARG99 | - |
| 2kav | Standard  (Carbamazepine) | SER1869, VAL1865, GLU1868 | GLU1788, PRO1789, LEU1866,  PRO1789,  VAL1865, LEU1866 | LEU1790, GLY1870, GLY1867 | - | - |
| 2kav | Kanzonol B  (PDFC32) | GLU1868, SER1869, SER1869, SER1869, VAL1865, GLU1868 | LEU1866, LEU1790, VAL1865 | PHE1795, THR1862, GLU1788, PRO1789, GLY1870 | - | - |
| 3dp4 | Crystallographic ligand  ((S)-Alpha-Amino-3-Hydroxy-5-Methyl-4-Isoxazolepropionic acid) | ARG112, GLU209, GLU209, THR107, ARG112, SER158, GLU209, PRO105, THR159, GLY157 | LEU154 | THR190, MET212, LEU208, LEU207, LYS160, TYR236, LEU106 | TYR77, GLU209 | - |
| 3dp4 | Standard  (Carbamazepine) | GLU209 | LEU154, LEU154, LEU208 | SER158, THR159, LEU207, PHE206, THR190, TYR236, THR211, MEY212, PRO105, TYR77 | GLU209 | - |
| 3dp4 | Kanzonol B  (PDFC32) | ARG96, ARG96, ARG64, ARG64 | ALA63, VAL95, VAL95 | GLU145 | - | - |
| 1pxo | Crystallographic ligand  ([4-(2-Amino-4-Methyl-Thiazol-5-YL)-Pyrimidin-2-YL]-(3-Nitro-Phenyl)-Amine) | GLU51 | PHE80, VAL18, ALA31, VAL64 | GLY147, ASP145, LEU55, ALA144, LEU134, LEU83, PHE82, GLU81, ILE10, LYS33 | - | - |
| 1pxo | Standard  (Carbamazepine) | ASP145 | VAL18, PHE80, PHE80, PHE80, VAL64, ALA144, LEU55, VAL64, ALA144, ALA31, LYS33 | PHE146, GLU51 | - | - |
| 1pxo | Kanzonol B  (PDFC32) | PHE146, LEU143, GLU51, ASP145 | LEU134, ALA144, PHE80, VAL18, ALA31, LEU55, VAL64 | GLN85, HIS84, ILE10, PHE82, LEU83, ILE63, LYS33 | - | - |
| 5y30 | Standard  (Carbamazepine) | - | ILE61, ILE61, ARG63 | ILE82, GLU81, PHE79, SER60, PRO62, PHE89, LEU88, GLY85, SER86, SER83 | - | - |
| 5y30 | Kanzonol B  (PDFC32) | ILE61, SER83, GLU81, SER83 | SER86 | THR80, SER60, PHE79, ARG63, GLY85, LEU88, GLU84. ILE82 | - | - |
| 3thj | Crystallographic ligand  (L-ornithine) | ASP128, ASP128, ASP128, ASP124, ASP234, ASP124, ASP128, ASP232, GLY142, ASP234 | - | SER137, ASN139, THR246, ASN130, ALA125 | GLU186, GLU277, HIS126, HIS141 | - |
| 3thj | Standard  (Carbamazepine) | SER137, ASN139, HIS141 | - | ASP181, HIS126, THR246, ARG21, GLN143, GLY142, ASN130, THR135, THR136 | ASP183, ASP183 | SER137 (Pi-Lone Pair) |
| 3thj | Kanzonol B  (PDFC32) | ASP128, SER137, ASN139, HIS141, GLU186, ASP183 | THR246 | PRO247, VAL248, ASP181, GLY142, THR127, ILE129, ASN130, HIS126 | - | - |

**Table S7.**

SMILES code for the compounds

| Compounds | SMILES Code |
| --- | --- |
| Diazepam | [H]C1=C([H])C([H])=C(C([H])=C1[H])C1=NC([H])([H])C(=O)N(C2=C1C([H])=C(Cl)C([H])=C2[H])C([H])([H])[H] |
| Carbamazepine | [H]N([H])C(=O)N1C2=C([H])C([H])=C([H])C([H])=C2C([H])=C([H])C2=C([H])C([H])=C([H])C([H])=C12 |
| Vigabatrin | [H]OC(=O)C([H])([H])C([H])([H])C([H])(N([H])[H])C([H])=C([H])[H] |
| PDFC1 | O[C@@H]1[C@@H](O)[C@H](O)[C@@H](CO)O[C@@H]1O[C@H]([C@@H](O)C=O)[C@H](O)[C@H](O)CO |
| PDFC2 | O[C@H]1[C@H](CO)O[C@H](OC(CO)CO)[C@@H](O)[C@@H]1O |
| PDFC3 | O[C@H]([C@@H]([C@@H](CO)O)O)[C@@H]([C@@H](CC(C(O)=O)=O)O)N |
| PDFC4 | O=CCCCCCCC/C=C\C=C\C=C |
| PDFC5 | OC1=CC(CC2=CN=C(N)N=C2N)=CC(OC)=C1OC |
| PDFC6 | OC([C@H]1N(C([C@@H](N)CC(C)C)=O)CCC1)=O |
| PDFC7 | O[C@H]1[C@H](C(O)=O)O[C@@H](N[C@@H]2[C@H](C2)C3=CC=CC=C3)[C@@H](O)[C@@H]1O |
| PDFC8 | OC/C(C)=C\CNC1=NC=NC2=C1NC=N2 |
| PDFC9 | O[C@@H](C)[C@@H](C(O)=O)NC([C@H](CC(C=C1)=CC=C1O)NC([C@H](CC(N)=O)N)=O)=O |
| PDFC10 | CC1(C)CC(C=C(C)[C@]1(/C=C/C(C)=C\C(O)=O)O)=O |
| PDFC11 | OC(C=C1C(C)=CC(OC1=C2)=O)=C2O |
| PDFC12 | CC(C)OC1=C([N+]([O-])=O)C=CC(C(N[C@@H](C(C)C)C(O)=O)=O)=C1 |
| PDFC13 | CN1C(SC[C@H](C(NCC(O)=O)=O)NC(CC[C@H](N)C(O)=O)=O)=C(N(=O)=O)N=C1 |
| PDFC14 | OC1=C(C2=CC(O)=CC=C2O)OC3=CC(O)=CC(O)=C3C1=O |
| PDFC15 | OC1=CC=CC=C1C2=CC(C3=C(O)C=C(O[C@@H]4O[C@H](CO)[C@@H](O)[C@H](O)[C@H]4O)C=C3O2)=O |
| PDFC16 | OC(C(C(C(OC(C(C=C1)=CC=C1O)=CC2=O)=C2C(O)=C3)=C3O)OC(CO)C4O)C4O |
| PDFC17 | O[C@@H]1[C@@H](O)[C@H](OC(C2=C(NC3=CC=CC(C)=C3C)C=CC=C2)=O)O[C@H](C(O)=O)[C@H]1O |
| PDFC18 | OC(CCCCCC/C=C/O/C=C/C=C\C/C=C\CC)=O |
| PDFC19 | OO[C@H](CCCCCCCC(O)=O)/C=C/[C@@H]1O[C@@H]1CCCCC |
| PDFC20 | O[C@@H](COC1=CC=C(C=C1)CCOCC2CC2)CNC(C)C |
| PDFC21 | OC(CCCCCCC#CCC#CCCCCCC)=O |
| PDFC22 | OC(/C=C/C(C=O)C(C(C)=O)C/C=C\CCCC(O)=O)CCCCC |
| PDFC23 | O[C@H]([C@@H]([C@@H](/C=C\CCCCC)O)O)CCCCCCCC(O)=O |
| PDFC24 | OC(CCCCCCC/C=C\C=C1O[C@@H]/1CCCCC)=O |
| PDFC25 | OC1=C2C(OC(C(C=C3)=CC=C3O)=CC2=O)=C(O)C=C1OC4=CC=C(C=C4)C(OC5=CC(O)=CC(O)=C56)=CC6=O |
| PDFC26 | O=C(CCC(O)=O)CCCC/C=C\C=C/C=C/C=C/CC |
| PDFC27 | [H]C1=C2C([H])([H])C([H])([H])[C@@]3([H])[C@]4([H])C([H])([H])C([H])([H])C(=O)[C@@]4(C([H])([H])[H])C([H])([H])C([H])([H])[C@]3([H])[C@@]2([H])C([H])([H])C([H])([H])C1=O |
| PDFC28 | O=C(CCCCCCCC(O)=O)/C=C/[C@@H]1O[C@@H]1CCCCC |
| PDFC29 | O=C(/C=C/C=C\C/C=C\C/C=C\CCCCC)CCCC(O)=O |
| PDFC30 | O=C(C(CC(N)=O)NC(C(CC(N)=O)N)=O)NC(C(O)=O)CCCN=C(N)N |
| PDFC31 | OC1C(C)(C)OC2=C(C(/C=C/C(C=C3)=CC=C3O)=O)C=CC(O)=C2C1 |
| PDFC32 | [H]OC1=C([H])C(O[H])=C(C([H])=C1[H])C(=O)C(\[H])=C(/[H])C1=C([H])C2=C(OC(C([H])=C2[H])(C([H])([H])[H])C([H])([H])[H])C([H])=C1[H] |
| PDFC33 | OC(CCCCCCC/C=C/C/C=C\C/C=C\CC)=O |
| PDFC34 | OC1C(/C=C/C(CCCCC)O)C(C/2)C(C1)OC2=C/CCCCCC(O)=O |
| PDFC35 | O[C@@H]([C@@H](/C=C/[C@@H](CCCCC)O)OCC)CCCCCCCC(O)=O |
| PDFC36 | O=C1C(C=C2)=C(C=C2OC)OC=C1 |
| PDFC37 | ClCC([C@@]1([C@](C[C@@H]2O)(C)[C@@H](C[C@H]1C)[C@H](CC3)[C@]2([C@@](C)(C=C4)C3=CC4=O)F)OC(CC)=O)=O |
| PDFC38 | O=C1N(CC2=CC=C(C3=CC=CC=C3C4=NNN=N4)C=C2)C(CCCC)=NC51CCCC5 |
| PDFC39 | OC([C@@H](NC([C@@H](NC([C@@H](N)CCCNC(N)=N)=O)[C@H](O)C)=O)CC1=CC=CC=C1)=O |
| PDFC40 | FC1=CC=C(C=C1)C2=C(/C=C/[C@@H](C[C@@H](CC(O)=O)O)O)N(C(C=C3)=C2C=C3O)C(C)C |
| PDFC41 | O=C1[C@@H](C/C=C\CCCC(O)=O)[C@@H](/C=C/[C@@H](CCCCC)O)C=C1 |
| PDFC42 | O=C(CC[C@@H](N)C(OCCCC)=O)OCCCC |
| PDFC43 | O=C(CCCCCCCCCCCCCCCCC)OC(/C=C\C/C=C\CC)/C=C\CCCCCCCC(O)=O |
| PDFC44 | O[C@H](C#CCCCCCCCCC(O)=O)CCCCCC |
| PDFC45 | OC(CCCCCCC/C=C/C/C=C\C/C=C\CC)=O |
| PDFC46 | O=C(CCCCCCCCCCCCCCC)N[C@H]1CCOC1=O |
| PDFC47 | O[C@H](C1)[C@@H](/C=C/[C@H](CCCCCC)O)[C@@H](CCCCCCC(O)=O)[C@H]1O |
| PDFC48 | OC(CCCCC/C=C/CC/C=C/C/C=C/CCCCC)=O |
| NDFC1 | O=C(C(C(O)=CC=C1O)=C1C2=O)C(C2=C3NCCNCCO)=C(C=C3)NCCNCCO |
| NDFC 2 | O=C([C@H](C)NC([C@@H](CCC(N)=O)N)=O)N[C@H](C(O)=O)CC(C=C1)=CC=C1O |
| NDFC3 | OC1=C2C(OC(C(C=C3)=CC=C3O)=CC2=O)=CC(O)=[C@]1[C@](C=C(C=C4)C(OC5=CC(O)=CC(O)=C56)=CC6=O)=C4O |
| NDFC4 | O[C@H]([C@@H](C/C=C\CCCCCO)O)CCCCCCCC(O)=O |
| NDFC5 | OO[C@H](CCCCCCCC(O)=O)/C=C/[C@H]1O[C@@H]1CCCCC |
| NDFC6 | O[C@@H]([C@@H](/C=C/[C@@H](CCCCC)O)OCC)CCCCCCCC(O)=O |
| NDFC7 | O=C(C1CCCCC1)N2CC(N3CCC4=CC=CC=C4[C@H]3C2)=O |
| NDFC8 | O[C@]1([C@@H]2O)[C@H]([C@H](O[C@@H]([C@@H]([C@H]3O)O)O[C@H](CO)[C@H]3O)OC=C1C)[C@@](C2)(C)OC(C)=O |
| NDFC9 | O[C@H]([C@@H]1[C@H]2[N@]3[C@H]4C[C@H]1[C@H](CC)[C@H]3O)[C@@]5(C2)[C@H]4N(C)C6=CC=CC=C56 |
| NDFC10 | O[C@@H](/C=C/C=C\CCCCCCCC(O)=O)CCCCC |
| NDFC11 | O[C@H](CCCC)CCCCCCCCC[C@@H](CCC(O)=O)O |
| NDFC12 | OC(CCCCCCC[C@H]1O[C@@H]1CCCCCCCC)=O |
| NDFC13 | O[C@@H](COC(C=C1)=CC=C1NC(C)=O)CNC(C)C |
| NDFC14 | Cl[C@H](C=O)CCCCCCCCCCCCCC |

**Table S8.**

Drug Likeness of phytochemicals from dabai fruits

| **Compounds** | **Molecular Weight** | **iLOGP** | **Hydrogen bond acceptor** | **Hydrogen bond donor** | **Rotatable bond** | **Topological Polar Surface Area** | **Lipinski** | **Veber** |
| --- | --- | --- | --- | --- | --- | --- | --- | --- |
| PDFC1 | 342.30 | 0.17 | 11 | 8 | 8 | 197.37 | 2 violations (N or O>10, NH or OH>5) | 1 violation (TPSA>140) |
| PDFC2 | 254.23 | 0.96 | 8 | 6 | 5 | 139.84 | 1 violation (NH or OH>5) | 0 violation |
| PDFC3 | 267.23 | -1.33 | 9 | 7 | 8 | 181.54 | 1 violation (NH or OH>5) | 1 violation (TPSA>140) |
| PDFC4 | 206.32 | 3.34 | 1 | 0 | 10 | 17.07 | 0 violation | 0 violation |
| PDFC5 | 276.29 | 1.82 | 5 | 3 | 4 | 116.51 | 0 violation | 0 violation |
| PDFC6 | 228.29 | 1.49 | 4 | 2 | 5 | 83.63 | 0 violation | 0 violation |
| PDFC7 | 309.31 | 1.49 | 7 | 5 | 4 | 119.25 | 0 violation | 0 violation |
| PDFC8 | 219.24 | 0.98 | 4 | 3 | 4 | 86.72 | 0 violation | 0 violation |
| PDFC9 | 396.40 | 0.03 | 8 | 7 | 12 | 205.07 | 2 violations (N or O>10, NH or OH>5) | 2 violations (Rotors>10, TPSA>140) |
| PDFC10 | 264.32 | 2.06 | 4 | 2 | 3 | 74.6 | 0 violation | 0 violation |
| PDFC11 | 192.17 | 1.52 | 4 | 2 | 0 | 70.67 | 0 violation | 0 violation |
| PDFC12 | 324.33 | 2.01 | 6 | 2 | 8 | 121.45 | 0 violation | 0 violation |
| PDFC13 | 432.41 | 0.94 | 10 | 5 | 14 | 247.76 | 1 violation (N or O>10) | 2 violations (Rotors>10, TPSA>140) |
| PDFC14 | 302.24 | 1.53 | 7 | 5 | 1 | 131.36 | 0 violation | 0 violation |
| PDFC15 | 432.38 | 1.54 | 10 | 6 | 4 | 170.05 | 1 violation (NH or OH>5) | 1 violation (TPSA>140) |
| PDFC16 | 432.38 | 1.38 | 10 | 7 | 3 | 181.05 | 1 violation (NH or OH>5) | 1 violation (TPSA>140) |
| PDFC17 | 417.41 | 2.17 | 8 | 5 | 6 | 145.55 | 0 violation | 1 violation (TPSA>140) |
| PDFC18 | 292.41 | 3.67 | 3 | 1 | 13 | 46.53 | 0 violation | 1 violation (Rotors>10) |
| PDFC19 | 328.44 | 3.75 | 5 | 2 | 15 | 79.29 | 0 violation | 1 violation (Rotors>10) |
| PDFC20 | 307.43 | 3.85 | 4 | 2 | 11 | 50.72 | 0 violation | 1 violation (Rotors>10) |
| PDFC21 | 276.41 | 4.06 | 2 | 1 | 10 | 37.30 | 1 violation (MLOGP>4.15) | 0 violation |
| PDFC22 | 352.47 | 2.25 | 5 | 2 | 15 | 91.67 | 0 violation | 1 violation (Rotors>10) |
| PDFC23 | 330.46 | 3.69 | 5 | 4 | 15 | 97.99 | 0 violation | 1 violation (Rotors>10) |
| PDFC24 | 294.43 | 3.84 | 3 | 1 | 13 | 49.83 | 0 violation | 1 violation (Rotors>10) |
| PDFC25 | 538.46 | 3.44 | 10 | 5 | 4 | 170.8 | 1 violation  (MW>500) | 1 violation  (TPSA>140) |
| PDFC26 | 290.40 | 3.27 | 3 | 1 | 12 | 54.37 | 0 violation | 1 violation (Rotors>10) |
| PDFC27 | 272.38 | 2.6 | 2 | 0 | 0 | 34.14 | 0 violation | 0 violation |
| PDFC28 | 310.43 | 3.25 | 4 | 1 | 14 | 66.90 | 0 violation | 1 violation (Rotors>10) |
| PDFC29 | 318.45 | 3.33 | 3 | 1 | 14 | 54.37 | 0 violation | 1 violation (Rotors>10) |
| PDFC30 | 402.41 | -0.71 | 8 | 8 | 15 | 272.10 | 2 violations (N or O>10, NH or OH>5) | 2 violations (Rotors>10, TPSA>140) |
| PDFC31 | 340.37 | 2.29 | 5 | 3 | 3 | 86.99 | 0 violation | 0 violation |
| PDFC32 | 322.35 | 3.1 | 4 | 2 | 3 | 66.76 | 0 violation | 0 violation |
| PDFC33 | 278.43 | 3.36 | 2 | 1 | 13 | 37.3 | 1 violation (MLOGP>4.15) | 1 violation (Rotors>10) |
| PDFC34 | 380.52 | 3.85 | 5 | 3 | 12 | 86.99 | 0 violation | 1 violation (Rotors>10) |
| PDFC35 | 358.51 | 3.96 | 5 | 3 | 17 | 86.99 | 0 violation | 1 violation (Rotors>10) |
| PDFC36 | 176.17 | 2.07 | 3 | 0 | 1 | 39.44 | 0 violation | 0 violation |
| PDFC37 | 466.97 | 3.10 | 6 | 1 | 5 | 80.67 | 0 violation | 0 violation |
| PDFC38 | 428.53 | 3.48 | 5 | 1 | 7 | 87.13 | 1 violation (MLOGP>4.15) | 0 violation |
| PDFC39 | 422.28 | 0.93 | 7 | 8 | 15 | 203.65 | 2 violations (N or O>10, NH or OH>5) | 2 violations (Rotors>10, TPSA>140) |
| PDFC40 | 427.47 | 2.59 | 6 | 4 | 8 | 102.92 | 0 violation | 0 violation |
| PDFC41 | 334.45 | 3.38 | 4 | 2 | 12 | 74.60 | 0 violation | 1 violation (Rotors>10) |
| PDFC42 | 259.34 | 2.16 | 5 | 1 | 12 | 78.62 | 0 violation | 1 violation (Rotors>10) |
| PDFC43 | 560.89 | 7.84 | 4 | 1 | 31 | 63.6 | 2 violations (MW>50, MLOGP>4.15) | 1 violation (Rotors>10) |
| PDFC44 | 296.44 | 4.00 | 3 | 2 | 13 | 57.53 | 0 violation | 1 violation (Rotors>10) |
| PDFC45 | 278.43 | 3.36 | 2 | 1 | 13 | 37.3 | 1 violation (MLOGP>4.15) | 1 violation (Rotors>10) |
| PDFC46 | 339.51 | 4.16 | 3 | 1 | 16 | 55.40 | 0 violation | 1 violation (Rotors>10) |
| PDFC47 | 370.52 | 3.41 | 5 | 4 | 14 | 97.99 | 0 violation | 1 violation (Rotors>10) |
| PDFC48 | 306.48 | 4.61 | 2 | 1 | 15 | 37.30 | 1 violation (MLOGP>4.15) | 1 violation (Rotors>10) |
| PDFC49 | 322.48 | 4.30 | 3 | 2 | 15 | 57.53 | 0 violation | 1 violation (Rotors>10) |
| NDFC1 | 444.48 | 3.03 | 8 | 8 | 12 | 163.18 | 1 violation (NH or OH>5) | 2 violations (Rotors>10, TPSA>140) |
| NDFC2 | 380.40 | 0.41 | 7 | 6 | 12 | 184.84 | 1 violation (NH or OH>5) | 2 violations (Rotors>10, TPSA>140) |
| NDFC3 | 538.46 | 3.06 | 10 | 6 | 3 | 181.8 | 2 violations (MW>500, NH or OH>5) | 1 violation  (TPSA>140) |
| NDFC4 | 330.46 | 3.24 | 5 | 4 | 16 | 97.99 | 0 violation | 1 violation (Rotors>10) |
| NDFC5 | 328.44 | 3.57 | 5 | 2 | 15 | 79.29 | 0 violation | 1 violation (Rotors>10) |
| NDFC6 | 358.51 | 3.96 | 5 | 3 | 17 | 86.99 | 0 violation | 1 violation (Rotors>10) |
| NDFC7 | 312.41 | 2.95 | 2 | 0 | 2 | 40.62 | 0 violation | 0 violation |
| NDFC8 | 420.41 | 2.36 | 11 | 6 | 5 | 175.37 | 2 violations (N or O>10, NH or OH>5) | 1 violation (TPSA>140) |
| NDFC9 | 326.43 | 0.77 | 3 | 2 | 1 | 46.94 | 0 violation | 0 violation |
| NDFC10 | 296.44 | 3.88 | 3 | 2 | 14 | 57.53 | 0 violation | 1 violation (Rotors>10) |
| NDFC11 | 316.48 | 3.96 | 4 | 3 | 16 | 77.76 | 0 violation | 1 violation (Rotors>10) |
| NDFC12 | 298.46 | 4.09 | 3 | 1 | 15 | 49.83 | 0 violation | 1 violation (Rotors>10) |
| NDFC13 | 266.34 | 2.50 | 4 | 3 | 8 | 70.59 | 0 violation | 0 violation |
| NDFC14 | 274.87 | 4.08 | 1 | 0 | 14 | 84.02 | 1 violation (MLOGP>4.15) | 1 violation (Rotors>10) |

All data obtained from SwissADME

**Table S9.**

Pharmacokinetic properties of phytochemicals of dabai fruits

| **Compounds** | **GI absorption*** | **BBB permeability**  **(log BB)**** | **CNS permeability**  **(log PS)**** | **BA Scores*** | **Metabolism*** | | | | |
| --- | --- | --- | --- | --- | --- | --- | --- | --- | --- |
|  |  |  |  |  | **CYP1A2 inhibitor** | **CYP2C19 inhibitor** | **CYP2C9 inhibitor** | **CYP2D6 inhibitor** | **CYP3A4 inhibitor** |
| PDFC1 | Low | -1.761 | -5.87 | 0.17 | No | No | No | No | No |
| PDFC2 | Low | -0.954 | -4.205 | 0.55 | No | No | No | No | No |
| PDFC3 | Low | -1.373 | -4.715 | 0.55 | No | No | No | No | No |
| PDFC4 | High | 0.771 | -1.567 | 0.55 | Yes | No | No | No | No |
| PDFC5 | High | -1.105 | -2.992 | 0.55 | No | No | No | No | No |
| PDFC6 | High | -0.807 | -3.091 | 0.55 | No | No | No | No | No |
| PDFC7 | High | -1.299 | -3.829 | 0.55 | No | No | No | No | No |
| PDFC8 | High | -1.249 | -3.552 | 0.55 | No | No | No | No | No |
| PDFC9 | Low | -1.447 | -4.55 | 0.17 | No | No | No | No | No |
| PDFC10 | High | -0.151 | -3.294 | 0.85 | No | No | No | No | No |
| PDFC11 | High | -0.053 | -2.194 | 0.55 | Yes | No | No | No | No |
| PDFC12 | High | -0.937 | -2.85 | 0.56 | No | Yes | No | No | No |
| PDFC13 | Low | -2.111 | -4.32 | 0.11 | No | No | No | No | No |
| PDFC14 | High | -1.463 | -3.344 | 0.55 | Yes | No | No | Yes | Yes |
| PDFC15 | Low | -1.549 | -4.105 | 0.55 | No | No | No | No | No |
| PDFC16 | Low | -1.723 | -4.023 | 0.55 | No | No | No | No | No |
| PDFC17 | Low | -1.271 | -3.682 | 0.56 | No | No | No | No | No |
| PDFC18 | High | -0.253 | -1.91 | 0.85 | No | No | Yes | Yes | No |
| PDFC19 | High | -0.736 | -3.033 | 0.56 | No | No | Yes | Yes | No |
| PDFC20 | High | -0.257 | -2.975 | 0.55 | No | No | No | Yes | No |
| PDFC21 | High | -0.204 | -1.403 | 0.85 | Yes | No | Yes | No | No |
| PDFC22 | High | -0.713 | -3.143 | 0.56 | No | No | No | Yes | Yes |
| PDFC23 | High | -1.144 | -3.488 | 0.56 | No | No | No | Yes | No |
| PDFC24 | High | -0.473 | -2.611 | 0.85 | Yes | Yes | Yes | Yes | No |
| PDFC25 | Low | -1.916 | -3.228 | 0.55 | No | No | Yes | No | No |
| PDFC26 | High | -0.275 | -2.529 | 0.85 | No | No | Yes | Yes | No |
| PDFC27 | High | 0.171 | -2.147 | 0.55 | No | No | No | No | No |
| PDFC28 | High | -0.409 | -2.959 | 0.85 | No | No | Yes | Yes | No |
| PDFC29 | High | -0.358 | -2.657 | 0.85 | No | No | Yes | No | No |
| PDFC30 | Low | -1.518 | -4.97 | 0.17 | No | No | No | No | No |
| PDFC31 | High | -0.79 | -2.283 | 0.55 | No | No | No | Yes | No |
| PDFC32 | High | -0.219 | -1.879 | 0.55 | Yes | Yes | Yes | No | Yes |
| PDFC33 | High | -0.282 | -1.563 | 0.85 | Yes | No | Yes | No | No |
| PDFC34 | High | -1.049 | -2.937 | 0.56 | No | No | Yes | Yes | No |
| PDFC35 | High | -1.262 | -3.436 | 0.56 | No | No | No | Yes | No |
| PDFC36 | High | 0.381 | -2.146 | 0.55 | Yes | No | No | No | No |
| PDFC37 | High | -0.61 | -2.924 | 0.55 | No | No | No | No | Yes |
| PDFC38 | High | -1.533 | -2.483 | 0.55 | Yes | Yes | Yes | Yes | Yes |
| PDFC39 | Low | -2.156 | -4.683 | 0.17 | No | No | No | No | No |
| PDFC40 | High | -1.33 | -3.146 | 0.56 | No | No | No | Yes | No |
| PDFC41 | High | -0.386 | -2.871 | 0.85 | No | No | Yes | Yes | No |
| PDFC42 | High | -0.462 | -3.016 | 0.55 | No | No | No | No | No |
| PDFC43 | Low | -1.146 | -2.918 | 0.85 | No | No | No | No | No |
| PDFC44 | High | -0.345 | -2.783 | 0.85 | Yes | No | Yes | Yes | No |
| PDFC45 | High | -0.282 | -1.563 | 0.85 | Yes | No | Yes | No | No |
| PDFC46 | High | -0.337 | -2.92 | 0.55 | Yes | Yes | Yes | No | No |
| PDFC47 | High | -1.053 | -3.345 | 0.56 | No | No | No | Yes | No |
| PDFC48 | High | -0.366 | -1.453 | 0.85 | Yes | No | Yes | No | No |
| NDFC1 | Low | -1.756 | -4.245 | 0.55 | No | No | No | No | No |
| NDFC2 | Low | -1.154 | -4.193 | 0.55 | No | No | No | No | No |
| NDFC3 | Low | -2.177 | -3.571 | 0.17 | No | No | No | No | No |
| NDFC4 | High | -1.231 | -3.729 | 0.56 | No | No | No | Yes | No |
| NDFC5 | High | -0.736 | -3.033 | 0.56 | No | No | Yes | Yes | No |
| NDFC6 | High | -1.262 | -3.436 | 0.56 | No | No | Yes | No | No |
| NDFC7 | High | 0.316 | -1.415 | 0.55 | No | Yes | No | Yes | Yes |
| NDFC8 | Low | -1.801 | -4.862 | 0.17 | No | No | No | No | No |
| NDFC9 | High | 0.123 | -1.879 | 0.55 | No | No | No | Yes | No |
| NDFC10 | High | -0.371 | -2.814 | 0.85 | Yes | No | Yes | Yes | No |
| NDFC11 | High | -1.129 | -3.327 | 0.56 | No | No | No | Yes | No |
| NDFC12 | High | -0.392 | -2.758 | 0.85 | Yes | No | Yes | Yes | No |
| NDFC13 | High | -0.603 | -2.919 | 0.55 | No | No | No | No | No |
| NDFC14 | High | 0.817 | -1.657 | 0.55 | Yes | No | Yes | No | No |
| Carbamazepine | High | 0.162 | -1.709 | 0.55 | Yes | Yes | No | No | No |
| Diazepam | High | 0.275 | -1.389 | 0.55 | Yes | Yes | Yes | Yes | Yes |
| Vigabatrin | High | -0.327 | -3.014 | 0.55 | No | No | No | No | No |

* Data obtained from SwissADME

** Data obtained from pkCSM

**Table S10.**

Toxicity profile of phytoconstituents from dabai fruits

| **Compounds** | **Toxicity** | | | | | | | | | | |  |
| --- | --- | --- | --- | --- | --- | --- | --- | --- | --- | --- | --- | --- |
|  | **LD_50_ Score (mg/kg)** | **Neuro-Toxicity** | **Hepato-Toxicity** | **Cardio-Toxicity** | **Nephro-Toxicity** | **Carcino-Genecity** | **Immuno-Toxicity** | **Muta-Genicity** | **Cyto-Toxicity** | **Clinical Toxicity** | **Toxicity Class** |  |
| PDFC1 | 18160 | Inactive | Inactive | Active | Active | Inactive | Inactive | Inactive | Inactive | Active | 6 |  |
| PDFC2 | 23000 | Inactive | Inactive | Active | Active | Inactive | Inactive | Inactive | Inactive | Inactive | 6 |  |
| PDFC3 | 9800 | Inactive | Inactive | Active | Active | Inactive | Inactive | Inactive | Inactive | Inactive | 6 |  |
| PDFC4 | 7563 | Active | Inactive | Inactive | Inactive | Inactive | Inactive | Inactive | Inactive | Inactive | 6 |  |
| PDFC5 | 3500 | Inactive | Inactive | Inactive | Active | Active | Active | Inactive | Inactive | Active | 5 |  |
| PDFC6 | 3000 | Inactive | Inactive | Active | Active | Inactive | Inactive | Inactive | Inactive | Active | 5 |  |
| PDFC7 | 245 | Inactive | Inactive | Inactive | Active | Inactive | Inactive | Inactive | Inactive | Active | 3 |  |
| PDFC8 | 160 | Active | Inactive | Inactive | Inactive | Inactive | Inactive | Inactive | Inactive | Active | 3 |  |
| PDFC9 | 1000 | Inactive | Inactive | Active | Active | Inactive | Inactive | Inactive | Inactive | Active | 4 |  |
| PDFC10 | 5000 | Inactive | Inactive | Inactive | Inactive | Inactive | Inactive | Inactive | Inactive | Inactive | 5 |  |
| PDFC11 | 3200 | Inactive | Inactive | Inactive | Active | Active | Inactive | Inactive | Inactive | Active | 5 |  |
| PDFC12 | 1500 | Inactive | Active | Inactive | Active | Inactive | Inactive | Active | Inactive | Inactive | 4 |  |
| PDFC13 | 5000 | Inactive | Inactive | Inactive | Active | Active | Inactive | Active | Inactive | Inactive | 5 |  |
| PDFC14 | 3919 | Inactive | Inactive | Inactive | Active | Inactive | Active | Inactive | Inactive | Inactive | 5 |  |
| PDFC15 | 5000 | Inactive | Inactive | Inactive | Active | Inactive | Active | Inactive | Inactive | Active | 5 |  |
| PDFC16 | 832 | Inactive | Inactive | Inactive | Active | Inactive | Inactive | Active | Inactive | Active | 4 |  |
| PDFC17 | 1000 | Inactive | Active | Active | Active | Inactive | Inactive | Inactive | Inactive | Active | 4 |  |
| PDFC18 | 20000 | Inactive | Inactive | Inactive | Active | Inactive | Inactive | Inactive | Inactive | Inactive | 6 |  |
| PDFC19 | 12760 | Inactive | Inactive | Inactive | Active | Inactive | Inactive | Inactive | Inactive | Inactive | 6 |  |
| PDFC20 | 48 | Inactive | Inactive | Inactive | Inactive | Inactive | Inactive | Inactive | Inactive | Active | 2 |  |
| PDFC21 | 1000 | Inactive | Inactive | Inactive | Inactive | Inactive | Inactive | Inactive | Inactive | Inactive | 4 |  |
| PDFC22 | 500 | Inactive | Inactive | Inactive | Inactive | Inactive | Inactive | Inactive | Inactive | Inactive | 4 |  |
| PDFC23 | 665 | Inactive | Inactive | Inactive | Inactive | Inactive | Inactive | Inactive | Inactive | Inactive | 4 |  |
| PDFC24 | 5000 | Inactive | Inactive | Inactive | Active | Inactive | Inactive | Inactive | Inactive | Inactive | 5 |  |
| PDFC25 | 3919 | Inactive | Inactive | Inactive | Active | Inactive | Inactive | Inactive | Inactive | Active | 5 |  |
| PDFC26 | 3200 | Inactive | Inactive | Inactive | Inactive | Inactive | Inactive | Inactive | Inactive | Inactive | 5 |  |
| PDFC27 | 2300 | Active | Inactive | Inactive | Inactive | Active | Active | Inactive | Inactive | Inactive | 5 |  |
| PDFC28 | 1890 | Inactive | Inactive | Inactive | Active | Inactive | Inactive | Inactive | Inactive | Active | 4 |  |
| PDFC29 | 10000 | Inactive | Inactive | Inactive | Inactive | Inactive | Inactive | Inactive | Inactive | Inactive | 6 |  |
| PDFC30 | 2000 | Inactive | Inactive | Active | Active | Inactive | Inactive | Inactive | Inactive | Inactive | 4 |  |
| PDFC31 | 3800 | Inactive | Inactive | Inactive | Inactive | Inactive | Active | Active | Inactive | Active | 5 |  |
| PDFC32 | 3800 | Inactive | Inactive | Inactive | Inactive | Inactive | Active | Inactive | Inactive | Active | 5 |  |
| PDFC33 | 10000 | Inactive | Inactive | Inactive | Inactive | Inactive | Inactive | Inactive | Inactive | Inactive | 6 |  |
| PDFC34 | 1100 | Inactive | Inactive | Inactive | Active | Inactive | Active | Inactive | Inactive | Active | 4 |  |
| PDFC35 | 6000 | Inactive | Inactive | Inactive | Active | Inactive | Inactive | Inactive | Inactive | Inactive | 6 |  |
| PDFC36 | 4000 | Inactive | Inactive | Inactive | Active | Active | Active | Active | Inactive | Inactive | 5 |  |
| PDFC37 | 3000 | Inactive | Inactive | Active | Inactive | Active | Active | Inactive | Inactive | Inactive | 5 |  |
| PDFC38 | 200 | Active | Inactive | Inactive | Active | Active | Inactive | Active | Inactive | Inactive | 3 |  |
| PDFC39 | 2400 | Inactive | Inactive | Active | Active | Inactive | Inactive | Inactive | Inactive | Active | 5 |  |
| PDFC40 | 416 | Active | Active | Inactive | Active | Inactive | Active | Inactive | Inactive | Active | 4 |  |
| PDFC41 | 400 | Inactive | Inactive | Inactive | Inactive | Inactive | Active | Inactive | Inactive | Active | 4 |  |
| PDFC42 | 6500 | Inactive | Inactive | Active | Active | Active | Inactive | Inactive | Inactive | Inactive | 6 |  |
| PDFC43 | 8000 | Inactive | Inactive | Inactive | Active | Inactive | Inactive | Inactive | Inactive | Inactive | 6 |  |
| PDFC44 | 1000 | Inactive | Inactive | Inactive | Inactive | Inactive | Inactive | Inactive | Inactive | Inactive | 4 |  |
| PDFC45 | 10000 | Inactive | Inactive | Inactive | Inactive | Inactive | Inactive | Inactive | Inactive | Inactive | 6 |  |
| PDFC46 | 1600 | Inactive | Inactive | Inactive | Active | Inactive | Inactive | Inactive | Inactive | Active | 4 |  |
| PDFC47 | 665 | Inactive | Inactive | Inactive | Inactive | Inactive | Inactive | Inactive | Inactive | Active | 4 |  |
| PDFC48 | 20000 | Inactive | Inactive | Inactive | Inactive | Inactive | Inactive | Inactive | Inactive | Inactive | 6 |  |
| NDFC1 | | 502 | Inactive | Inactive | Inactive | Active | Inactive | Active | Active | Active | Active | 4 |
| NDFC2 | | 2400 | Inactive | Inactive | Active | Inactive | Inactive | Inactive | Inactive | Inactive | Active | 5 |
| NDFC3 | | 3919 | Inactive | Inactive | Inactive | Active | Inactive | Active | Inactive | Inactive | Active | 5 |
| NDFC4 | | 11800 | Inactive | Inactive | Inactive | Active | Inactive | Inactive | Inactive | Inactive | Inactive | 2 |
| NDFC5 | | 12760 | Inactive | Inactive | Inactive | Active | Inactive | Inactive | Inactive | Inactive | Inactive | 6 |
| NDFC6 | | 6000 | Inactive | Inactive | Inactive | Active | Inactive | Inactive | Inactive | Inactive | Inactive | 6 |
| NDFC7 | | 2454 | Inactive | Inactive | Inactive | Inactive | Inactive | Inactive | Inactive | Inactive | Active | 5 |
| NDFC8 | | 2000 | Inactive | Inactive | Active | Active | Inactive | Active | Inactive | Inactive | Inactive | 4 |
| NDFC9 | | 34 | Active | Inactive | Inactive | Inactive | Inactive | Inactive | Inactive | Inactive | Active | 2 |
| NDFC10 | | 3200 | Inactive | Inactive | Inactive | Inactive | Inactive | Inactive | Inactive | Inactive | Inactive | 5 |
| NDFC11 | | 20 | Inactive | Inactive | Inactive | Inactive | Inactive | Inactive | Inactive | Inactive | Inactive | 2 |
| NDFC12 | | 16000 | Inactive | Inactive | Inactive | Active | Inactive | Inactive | Inactive | Inactive | Inactive | 6 |
| NDFC13 | | 3458 | Inactive | Inactive | Inactive | Active | Inactive | Inactive | Inactive | Inactive | Active | 5 |
| NDFC14 | | 5000 | Inactive | Inactive | Inactive | Inactive | Active | Inactive | Active | Inactive | Inactive | 5 |
| Carbamazepine | 529 | Active | Active | Inactive | Inactive | Inactive | Inactive | Inactive | Inactive | Inactive | 4 |  |
| Diazepam | 48 | Active | Inactive | Inactive | Inactive | Inactive | Inactive | Inactive | Active | Active | 2 |  |
| Vigabatrin | 3000 | Active | Inactive | Active | Inactive | Inactive | Inactive | Inactive | Inactive | Inactive | 5 |  |

All data obtained from ProTox
